# Vps1 drives membrane constriction and fission necessary for endosomal protein sorting

**DOI:** 10.1038/s44318-026-00855-4

**Published:** 2026-07-20

**Authors:** Shilpa Gopan, Uma Swaminathan, Gurmail Singh, Thomas J Pucadyil

**Affiliations:** 1 Indian Institute of Science Education and Research, Dr. Homi Bhabha Road, Pashan, Pune, 411008 Maharashtra India; 2 Biotechnology Research Innovation Council - National Centre for Cell Science, Savitribai Phule Pune University Campus, Ganeshkhind Road, Pune, 411007 Maharashtra India

**Keywords:** Membranes & Trafficking

## Abstract

The trafficking of cargo between endosomes and the Golgi apparatus uses both retromer-dependent and retromer-independent routes. Disruptions to these routes lead to the mis-sorting of lysosomal cargo, and associated metabolic and neurological disorders. The yeast dynamin Vps1 is essential for these trafficking pathways; however, it is not clear whether it directly causes membrane fission. Using cell-free reconstitution and live-cell assays, here we demonstrate that Vps1 assembles into scaffolds on membrane tubules, and uses GTP hydrolysis to force tubule constriction and fission. Vps1 mutants that are unable to assemble or to hydrolyze GTP fail to achieve fission in vitro and cause cargo mis-sorting in vivo. Furthermore, we identify two essential motifs, a lysine-rich phosphoinositide-binding motif and a phenylalanine-rich self-assembly motif, which, when mutated, render Vps1 dysfunctional. Finally, quantitative proteomics revealed a broad range of Golgi and plasma membrane proteins that mis-sort to the vacuole without Vps1. These findings define the Vps1-dependent retrograde pathway’s cargo repertoire and confirm Vps1’s mechanochemical role in membrane fission.

## Synopsis

Repeated cycles of membrane constriction and relaxation are thought to generate the mechanical stress required for membrane fission. This study shows that the yeast dynamin Vps1 coordinates these cycles by coupling GTP hydrolysis to scaffold remodeling during retrograde trafficking.

- On endosome tubules below a radius of 30 nm, Vps1 forms scaffolds that actively constrict them further.
- Vps1 scaffolds hydrolyse GTP, powering the thinning-relaxation cycles that lead to fission.
- This function requires a lysine-rich phosphoinositide-binding motif and a phenylalanine-rich self-assembly motif.
- Disruption of this Vps1 function causes Golgi and plasma membrane proteins to mis-sort to the vacuole.

## Abstract (web summary)

Vps1 is a constricting scaffold that powers endosomal membrane fission.

## Graphical abstract

**Figure:**
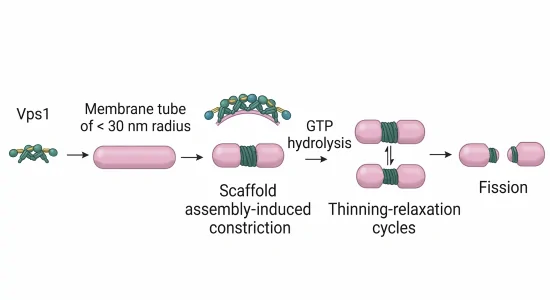

**Figure:**
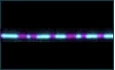

## Introduction

The homeostatic maintenance of organelle identity and function relies on intricate, finely controlled protein sorting and trafficking pathways across the endolysosomal system (Cullen, 2008). The foundational design principles of these pathways are evolutionarily conserved (Wendland et al, 1998; Burd and Cullen, 2014), and their disruption is linked to diverse pathophysiologies (Lane et al, 2012; Sebastian et al, 2012; Small and Petsko, 2015; Vagnozzi and Praticò, 2019; Cullen et al, 2024). Intracellular trafficking requires the concerted action of adapters, coats, and fission proteins to release cargo-laden transport carriers from a donor compartment, which subsequently fuse with an acceptor compartment to deliver their contents. In yeast, the vacuolar protein sorting (VPS) screen identified numerous proteins critical for cargo trafficking between the trans-Golgi network (TGN) and endosomes or pre-vacuolar compartments (PVC) (Bankaitis et al, 1986; Rothman and Stevens, 1986). Vacuolar protein sorting-associated protein 1 (Vps1) was discovered through this screen (Rothman and Stevens, 1986; Robinson et al, 1988). Since its discovery, Vps1 has been implicated in regulating vacuole and peroxisome morphology (Raymond et al, 1992; Peters et al, 2004; Kuravi et al, 2006; Vizeacoumar et al, 2006; Röthlisberger et al, 2009; Ekal et al, 2023), endocytosis (Nannapaneni et al, 2010; Rooij et al, 2010; Hayden et al, 2013; Palmer et al, 2015; Smaczynska-de Rooij et al, 2016, 2019), and autophagy (Arlt et al, 2023; Hu and Reggiori, 2023).

Vps1 coordinates with the retromer complex to transport specific cargo, such as the carboxypeptidase Y (CPY) receptor Vps10, from endosomes back to the TGN. Disruptions in this pathway cause the mis-sorting of Vps10 to the vacuole and the subsequent aberrant secretion of CPY—the foundational phenotype that enabled the original VPS screen (Marcusson et al, 1994; Nothwehr et al, 1995; Cooper and Stevens, 1996; Arlt et al, 2014; Chi et al, 2014). Mechanistically, the retromer complex retrieves cargo from endosomes (Horazdovsky et al, 1997; Seaman et al, 1998; Burda et al, 2002; Chi et al, 2014; Day et al, 2018; Ma et al, 2017a). This complex consists of a Vps26/Vps29/Vps35 core that manages cargo sorting, which associates with Vps5/Vps17 SNX-BAR proteins to tubulate the membrane; their coordinated activities generate cargo-laden membrane tubules. Although the exact mechanism of Vps1 recruitment to the retromer complex remains unclear, Vps1 localizes to SNX-BAR tubules emerging from endosomes undergoing fission. Furthermore, cells lacking Vps1 exhibit hyper-elongated SNX-BAR-coated tubules (Arlt et al, 2014; Chi et al, 2014; Ma et al, 2017a; Suzuki et al, 2021). While these observations strongly support a model where Vps1 mediates membrane fission, such a distinct mechanical role has not yet been directly validated.

Vps1 belongs to the dynamin superfamily. Classic dynamin self-assembles into helical scaffolds on membranes, a process that stimulates their basal GTPase activity (Ford and Chappie, 2019). However, structural studies reveal that helical scaffolds of Vps1 adopt an overall architecture that is more open than that of endocytic dynamins (Varlakhanova et al, 2018; Ford and Chappie, 2019). Crucially, these Vps1 scaffolds lack the critical interdomain contacts necessary for the assembly-stimulated GTPase activity observed in classic dynamins. Consequently, Vps1 exhibits no significant stimulation of GTPase activity upon membrane binding (Varlakhanova et al, 2018), which challenges prevailing models since assembly-stimulated GTPase activity is considered a fundamental requirement for dynamin-mediated membrane fission (Antonny et al, 2016; Jimah and Hinshaw, 2019).

Here, we interrogate Vps1 function using cell-free reconstitution assays on membrane templates that mimic tubulated endosomes, correlating these mechanical insights with endosomal protein sorting in vivo. Furthermore, we perform quantitative proteomic analysis of vacuoles isolated from wild-type and vps1Δ cells to globally map the repertoire of cargo proteins that rely on Vps1 for their intracellular trafficking.

## Results

### Vps1 catalyzes the fission of tubular membrane templates

**Figure 1:**
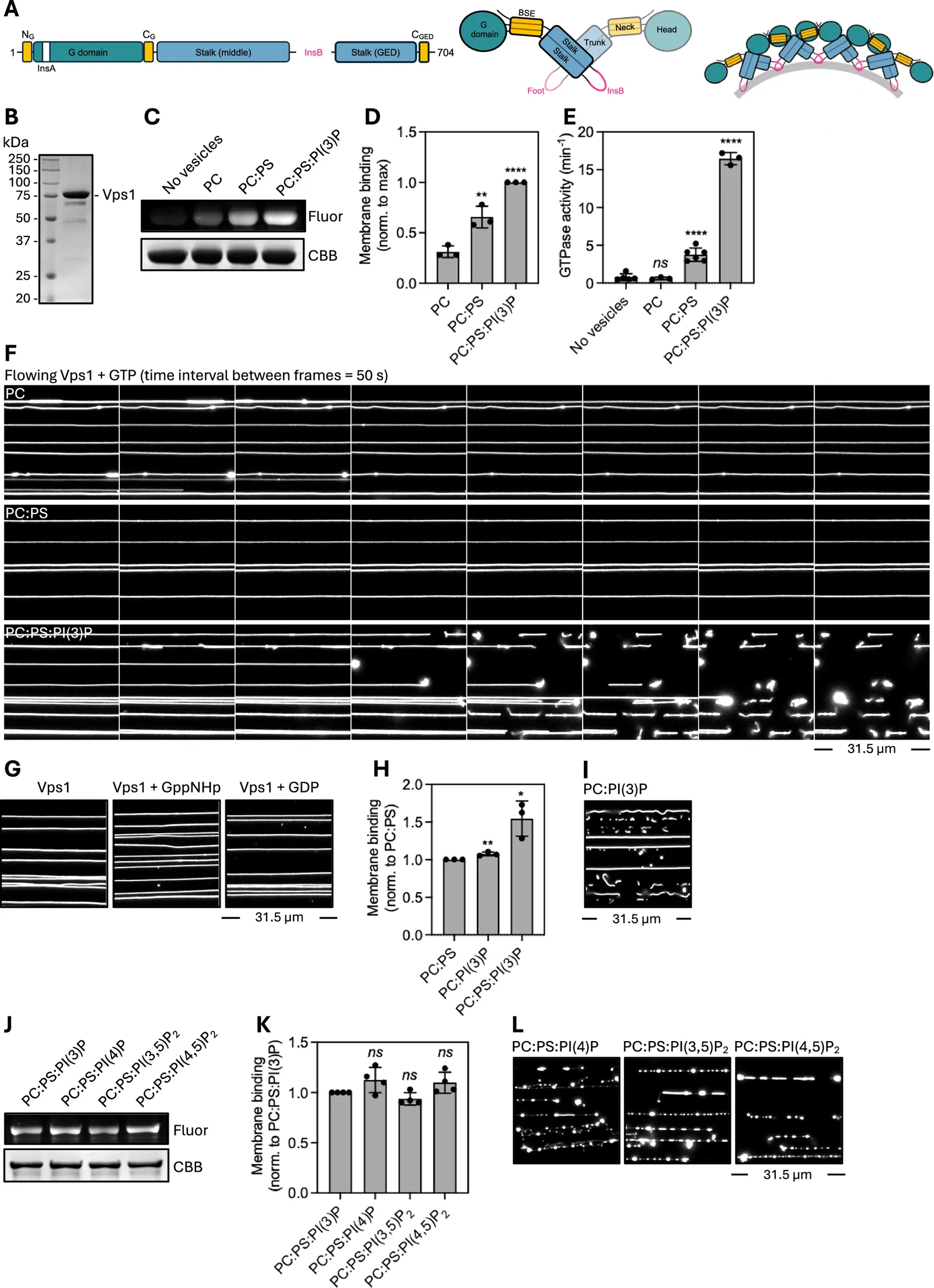
Vps1 catalyzes the fission of tubular membrane templates. (**A**) Schematic of Vps1 domain organization and self-assembly architecture. (**B**) Coomassie Brilliant Blue (CBB)-stained gel showing purified Vps1. (**C**) PLiMAP gel showing Vps1 fluorescence (fluor) as an indicator of membrane binding to vesicles, with CBB showing protein loading. (**D**) Normalized Vps1 membrane binding relative to PC:PS:PI(3)P. Data were mean ± SD (*N* = 3 technical replicates), with statistical significance compared against PC using an unpaired t-test (**p = 0.0083, ***p < 0.0001). (**E**) Vps1 GTPase activity with or without vesicles of the indicated compositions. Data were mean ± SD (*N* ≥ 3 technical replicates), with statistical significance compared against no vesicles using an unpaired *t*-test (ns not significant, ***p < 0.0001). (**F**) Time-lapse frames after flowing Vps1 with GTP onto SMrTs of the indicated lipid composition. (**G**) SMrT morphology after 10 min of exposure to Vps1 with different nucleotides. (**H**) Normalized Vps1 membrane binding relative to PC:PS. Data were mean ± SD (*N* = 3 technical replicates), and statistical significance was compared against PC:PS using an unpaired *t*-test (**p = 0.0069, ***p = 0.0157). (**I**) SMrT morphology of the indicated lipid composition after 10 min of exposure to Vps1 with GTP. (**J**) PLiMAP gel showing binding of Vps1 to vesicles of the indicated composition. (**K**) Normalized Vps1 membrane binding relative to PC:PS:PI(3)P. Data were mean ± SD (*N* = 4 technical replicates), with statistical significance compared against PC:PS:PI(3)P using an unpaired *t*-test (ns not significant). (**L**) SMrT morphology after 10 min of exposure to Vps1 with GTP. Source data are available online for this figure.

Vps1 is structurally characterized by a GTP-binding G domain and a stalk comprising a middle domain and a GTPase effector domain (GED) (Fig. 1A) (Varlakhanova et al, 2018; Ford and Chappie, 2019; Jimah and Hinshaw, 2019). The G domain is coupled to the stalk via the bundle signaling element (BSE), which is formed by the N- and C-termini of the G domain and the C-terminus of the GED. Situated between the middle domain and the GED is Insert B (InsB), the primary region responsible for membrane interactions. Stalk-mediated interactions promote Vps1 self-assembly into soluble oligomers and membrane-bound scaffolds. Within these scaffolds, subsequent inter-rung G domain interactions stimulate GTP hydrolysis. To determine the intrinsic biochemical and mechanical properties of Vps1, we purified a bacterially expressed recombinant construct and evaluated its behavior on various membrane templates (Fig. 1B). Endolysosomal membranes are enriched in phosphatidylserine (PS) and a diverse cohort of phosphoinositides—including PI(3)P, PI(4)P, PI(3,5)P₂, and PI(4,5)P₂—which dynamically interconvert during endosome maturation (Yeung et al, 2008; Marat and Haucke, 2016; Posor et al, 2022). While prior studies evaluated Vps1 membrane binding using vesicle sedimentation assays (Smaczynska-de Rooij et al, 2019), these assays suffer from low sensitivity and necessitate unphysiologically high concentrations (~20 mol%) of phosphoinositides. To overcome this limitation, we utilized the highly sensitive proximity-based labeling of membrane-associated proteins (PLiMAP) assay to profile the lipid-binding preferences of Vps1 (Jose et al, 2020). PLiMAP exploits a photoactivatable fluorescent lipid (TDPE) that covalently crosslinks with membrane-proximal proteins upon UV irradiation; crosslinked samples are subsequently resolved by SDS-PAGE and quantified via TDPE fluorescence. We assessed Vps1 binding to membrane vesicles containing phosphatidylcholine (PC), PS, and a representative endosomal phosphoinositide, PI(3)P. PLiMAP revealed minimal baseline binding of Vps1 to pure PC vesicles. However, the sequential inclusion of 15 mol% PS and 5 mol% PI(3)P progressively and robustly enhanced membrane recruitment (Fig. 1C,D). Because classic dynamins exhibit assembly-stimulated GTPase activity upon membrane binding (Ford and Chappie, 2019), we next evaluated whether this holds true for Vps1. In the absence of membranes, recombinant Vps1 displayed a basal GTPase turnover rate of ~0.6 min⁻¹ (Fig. 1E), consistent with previous reports (Varlakhanova et al, 2018). While PC vesicles had no effect on this baseline, PC:PS vesicles induced an approximately ~6-fold stimulation, and PC:PS:PI(3)P vesicles elicited a ~30-fold stimulation of the basal GTPase rate (Fig. 1E). These results confirm that our recombinant Vps1 preparation is enzymatically active and responsive to specific lipid cues. Given that Vps1 localizes to tubular endosomes undergoing severing in vivo (Arlt et al, 2014; Chi et al, 2014; Suzuki et al, 2021), we leveraged Supported Membrane Templates (SMrTs) to monitor fission on an array of PEG-cushioned membrane tubules (Dar et al, 2017; Swaminathan and Pucadyil, 2024). Perfusion of Vps1 with GTP had no effect on PC or PC:PS tubules. In stark contrast, templates containing PC:PS:PI(3)P underwent rapid and widespread severing (Fig. 1F; Movie EV1), demonstrating that Vps1 is sufficient to catalyze membrane fission. To determine the nucleotide requirements of this process, we performed reactions with either GDP, the non-hydrolyzable GTP analog GppNHp, or in the total absence of nucleotide; none of these conditions supported tubule fission (Fig. 1G). These data establish that active GTP hydrolysis is an absolute requirement for Vps1-mediated membrane fission.

To dissect the cooperative contribution of PS and phosphoinositides, we analyzed Vps1 membrane binding and fission on PI(3)P-containing membranes lacking PS. Vps1 bound to PC:PI(3)P vesicles at levels comparable to PC:PS vesicles (Fig. 1H). Notably, however, tubule fission occurred exclusively on templates containing phosphoinositides (Fig. 1I), highlighting their unique role in mechanically activating Vps1. The addition of PS to PI(3)P-bearing membranes further optimized recruitment (Fig. 1H), suggesting that phosphoinositides presented within a PS-rich membrane environment provide the ideal platform for Vps1 binding and mechanical activation. Finally, because maturing endosomes also harbor other phosphoinositides such as PI(4)P, PI(3,5)P₂, and PI(4,5)P₂, we tested Vps1 functionality on vesicles containing 15 mol% PS supplemented with 5 mol% of each respective phosphoinositide species. PLiMAP tracking revealed uniform binding across all tested phosphoinositide-bearing vesicles, each of which supported robust tubule fission (Fig. 1J–L). Taken together, these results indicate that while phosphoinositides are strictly required to license Vps1-mediated fission, the enzyme exhibits broad promiscuity toward specific phosphoinositide headgroups.

### Vps1-catalyzed membrane fission is acutely sensitive to membrane curvature

**Figure 2:**
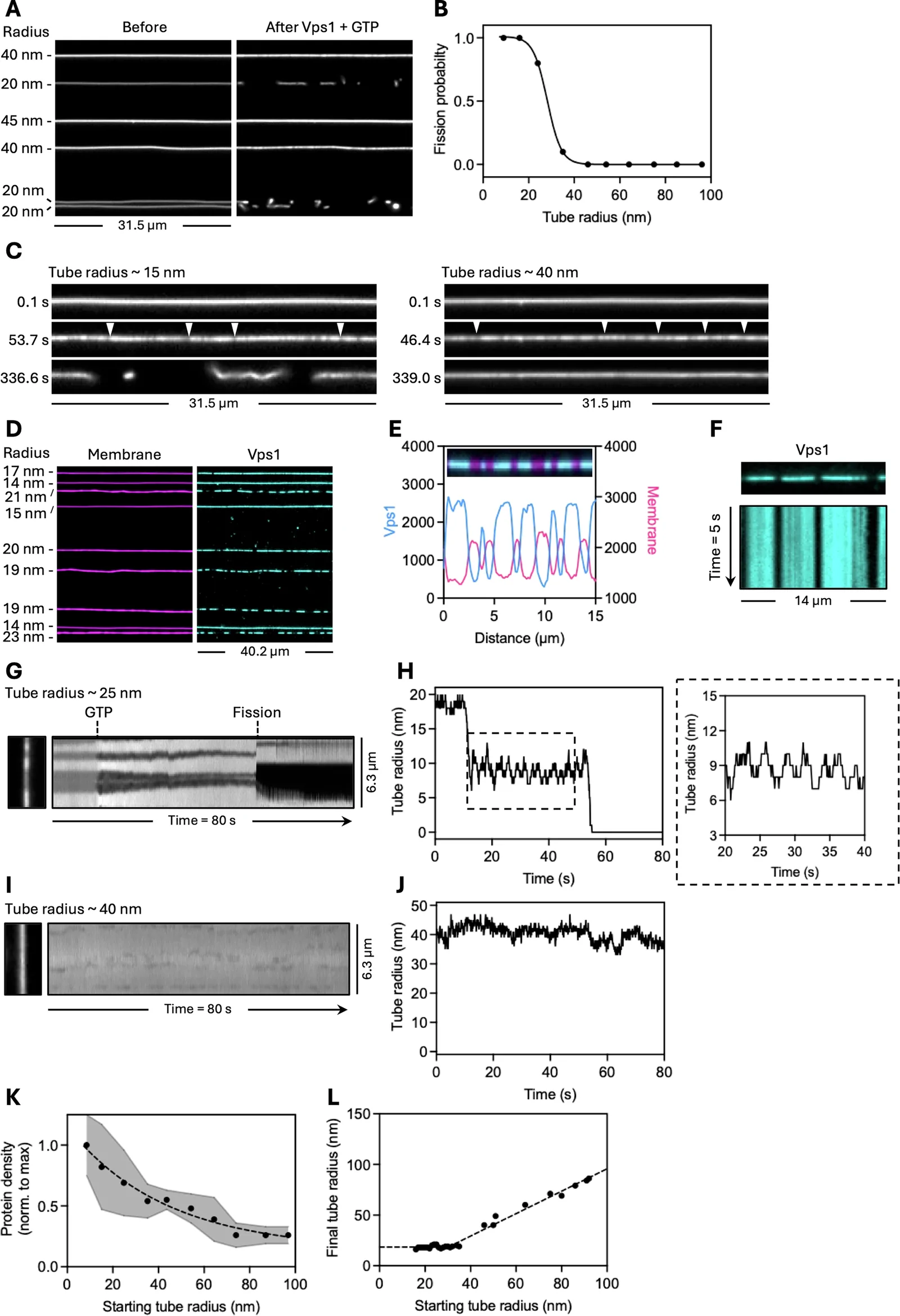
Mechanism of Vps1-catalyzed membrane fission. (**A**) Representative images of the same SMrT field acquired before and after the addition of Vps1 with GTP. (**B**) Fission probability plotted as a function of starting tube radius. Fission probability is defined as the fraction of tubes displaying at least one cut. Data from two technical replicates were parsed into 10-nm bins, and the probability is plotted against the mean tube size within each bin (*n* = 6 tubes for <10 nm, 69 for 10–20 nm, 33 for 20–30 nm, 19 for 30–40 nm, 22 for 40–50 nm, 13 for 50–60 nm, 10 for 60–70 nm, 4 for 70–80 nm, 7 for 80–90 nm, and 2 for 90–100 nm). (**C**) Time-lapse frames after flowing Vps1 with GTP on a thin (~15 nm radius) and thick (~40 nm radius) membrane tube. White arrowheads highlight sites of constriction. (**D**) Distribution of Ax488-labeled Vps1 on membrane tubes in the presence of GppNHp. (**E**) Ax488-labeled Vps1 with GppNHp localized on a tube alongside the corresponding fluorescence line profile. (**F**) Representative kymograph from a time-lapse movie of a membrane tube coated with Ax488-labeled Vps1 in the presence of GppNHp. (**G**) Kymograph showing GTP addition to a tube with a ~25 nm mean starting radius preincubated with Vps1 and GppNHp. (**H**) Quantification of tube radius under the Vps1 scaffold following GTP addition. The inset highlights the cycles of thinning and relaxation of the super-constricted intermediate. (**I**) Kymograph showing GTP addition to a tube with a ~40 nm mean starting radius preincubated with Vps1 and GppNHp. (**J**) Plot showing the corresponding tube radius over time after GTP addition. (**K**) Fluorescence intensity of Ax488-labeled Vps1 on membrane tubes plotted against the initial tube radius. Data from a single experiment were parsed into 10-nm bins, and the mean fluorescence is plotted against the mean tube size within each bin (*n* = 5 tubes for <10 nm, 13 for 10–20 nm, 6 for 20–30 nm, 9 for 30–40 nm, 11 for 40–50 nm, 9 for 50–60 nm, 9 for 60–70 nm, 5 for 70–80 nm, 3 for 80–90 nm, and 3 for 90–100 nm). The shaded gray region represents the SD. (**L**) Plot comparing the radius of the same tube before and after incubation with Vps1 and GppNHp from a single experiment, fitted to a segmental linear regression. Source data are available online for this figure.

**Figure EV1:**
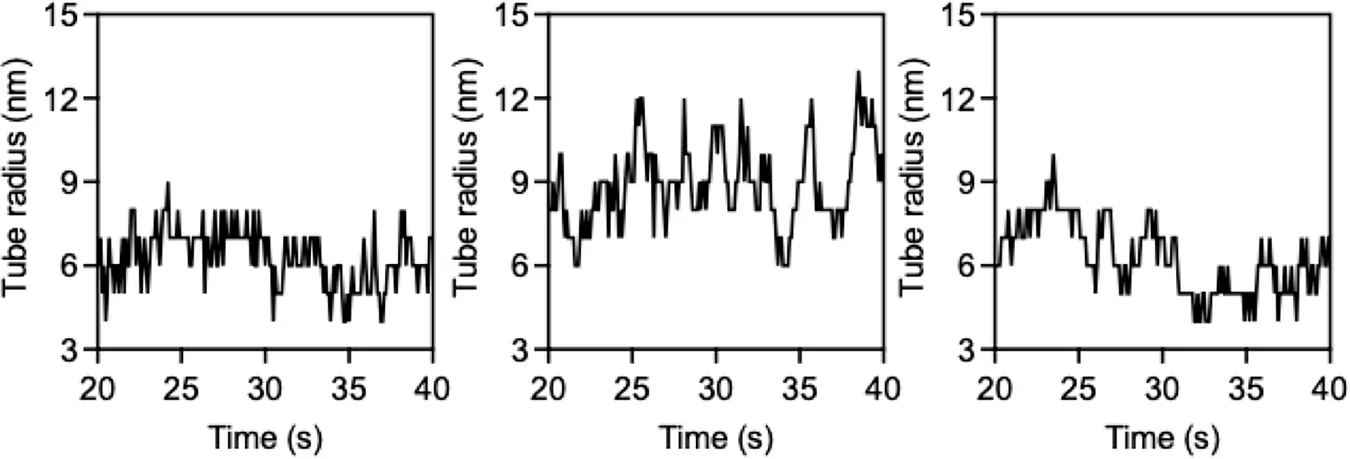
Pathway to Vps1-catalyzed membrane fission. Plots showing cycles of thinning and relaxation of the super-constricted intermediate on three tubes. Source data are available online for this figure.

Fission dynamin self-assembles into helical scaffolds around tubular membranes, meaning that the underlying topology and curvature of the membrane tube are expected to fundamentally dictate their mechanical functions (Kamerkar et al, 2018; Ford and Chappie, 2019; Jimah and Hinshaw, 2019; Khurana et al, 2023; Sarkar and Pucadyil, 2025). Within the retromer pathway, SNX-BAR proteins tubulate membranes; their capacity to define membrane curvature could regulate the binding and self-assembly of Vps1, which in turn would determine the outcome of fission reactions. We therefore systematically analyzed how Vps1 function is modulated by membrane curvature. Imaging a representative field of SMrTs before and after the perfusion of Vps1 and GTP revealed that tubules with a radius of ~20 nm were successfully severed, whereas wider tubules with radii of ~40 nm and above remained intact (Fig. 2A). Tubule radii were calculated using a fluorescence intensity-based calibration procedure detailed in the Methods section. To quantify these observations, we plotted fission probability (defined as the fraction of tubules exhibiting at least one scission event) against the initial tubule radius. Tubules thinner than ~30 nm in radius displayed a high fission probability, which dropped precipitously as tubule radius increased (Fig. 2B). Time-lapse imaging of a narrow tubule (~15 nm radius) versus a wide tubule (~40 nm radius) upon Vps1 and GTP perfusion revealed localized dimming of the membrane fluorescence (Fig. 2C, white arrowheads). Because these membrane tubules are diffraction-limited structures, local dimming of fluorescence signifies physical thinning or constriction. These constrictions appeared on both thin and thick tubules at early time points, reflecting the intrinsic propensity of Vps1 to scaffold the membrane. On the narrow tubule, these constrictions persisted, ultimately culminating in tubule scission (Fig. 2C; Movie EV2). Conversely, constrictions on the wide tubule spontaneously relaxed over time, allowing the tubule to revert to its initial morphology (Fig. 2C; Movie EV2). Because the constrictions captured during ongoing fission reactions were transient and difficult to characterize, we reconstituted the reaction in a stage-wise manner. We first allowed Vps1 to bind the membrane templates in the presence of GppNHp. Parallel experiments performed with Alexa Fluor 488-labeled Vps1 (Ax488-Vps1) revealed that the protein distributed uniformly along some tubules but coalesced into discrete foci on others (Fig. 2D). This heterogeneity was heavily dependent on initial tubule size, with discrete focal assemblies favored on tubules wider than ~15 nm in radius (Fig. 2D). On tubules displaying discrete Vps1 foci, the underlying membrane fluorescence beneath the assembly was notably dimmer than adjacent, Vps1-devoid regions (Fig. 2E), confirming active constriction. Thus, the transient constrictions observed previously (Fig. 2C) reflect the physical assembly of a stable Vps1 scaffold, which kymographs confirmed remained stationary over time (Fig. 2F). We next introduced GTP to these pre-assembled, stable scaffolds. On narrow, scaffolded tubules, the arrival of GTP induced sudden, severe constriction, as captured by kymography and corresponding line profiles (Fig. 2G,H; Movie EV3). Because the longitudinal length of the scaffolded region in these experiments exceeded the diffraction limit, we could accurately track the radius of the underlying membrane. The pre-scaffolded tubule possessed a radius of ~20 nm, which underwent rapid thinning to a super-constricted intermediate state upon GTP hydrolysis (Fig. 2G,H). High-temporal analysis revealed distinct cycles of thinning and relaxation within this super-constricted intermediate (Fig. 2H), a behavior consistently recapitulated across multiple independent tubules (Fig. EV1). During these structural oscillations, the tubule lumen repeatedly reached highly constricted, narrow dimensions before eventually snapping. This behavior indicates that stable self-assembly into a scaffold, coupled with assembly-stimulated GTPase activity, drives the membrane into a highly unstable, super-constricted state. The repeating cycles of thinning and relaxation may reflect a steady state, where active GTPase cycles force mechanical constriction while partial, localized disassembly permits transient relaxation. In contrast, wide tubules subjected to the same sequential treatment exhibited only shallow, highly transient constrictions (Fig. 2I,J). To determine the biophysical basis for why Vps1-mediated fission is tightly regulated by starting tubule size, we quantified both the membrane-binding and the scaffolding capacity of Vps1 across a range of tubule diameters. Utilizing the GppNHp-bound Ax488-Vps1 imaging datasets (Fig. 2D), we calculated the membrane density of Vps1 (the ratio of Vps1 fluorescence to membrane fluorescence) and plotted it as a function of the initial tubule radius. This analysis revealed a shallow, inverse relationship between Vps1 density and tubule size (Fig. 2K), indicating that Vps1 binding possesses modest curvature sensitivity. However, membrane constriction serves as a truer functional reporter of the protein’s capacity to productively self-assemble into a structural scaffold. We therefore mapped the radius of tubules before and after incubation with Vps1 and GppNHp. Remarkably, tubules with an initial radius below ~30 nm were uniformly constricted down to a fixed radius of ~20 nm, whereas tubules wider than ~30 nm strictly resisted constriction (Fig. 2L). Notably, while these wider tubules retained robust protein density (Fig. 2K), they completely failed to constrict the membrane (Fig. 2L). This demonstrates that while baseline membrane binding is only weakly curvature-dependent, the higher-order self-assembly required for scaffold constriction is acutely sensitive to a curvature threshold. Vps1-mediated fission is thus strictly restricted to narrow tubules because the enzyme cannot overcome the mechanical resistance required to assemble a constricting scaffold on tubules wider than a critical radius of approximately 30 nm. Intriguingly, once the super-constricted intermediate was achieved on narrow tubules, it persisted for an extended duration before scission finally occurred (Movie EV3). This stands in stark contrast to mammalian endocytic and mitochondrial dynamins, where GTP hydrolysis-induced constriction triggers near-instantaneous fission (Dar and Pucadyil, 2017; Pemberton et al, 2025). Instead, this prolonged intermediate phenotype closely resembles a previously characterized mammalian endocytic dynamin mutant in which the structured, membrane-inserting Pleckstrin homology domain (PHD) was replaced with a flexible, unstructured lipid-binding linker (Dar and Pucadyil, 2017). While specific hydrophobic residues within the classic dynamin PHD actively insert into the lipid bilayer to destabilize the membrane and accelerate fission (Baratam et al, 2021; Khurana et al, 2023), Vps1 naturally lacks a PHD, possessing an unstructured Insert B region instead. Consequently, the slower tubule-snapping kinetics observed here likely reflect the unique, non-inserting membrane-binding modality governed by the residues within Insert B.

### Vps1-catalyzed fission is required for Vps10 trafficking

**Figure 3:**
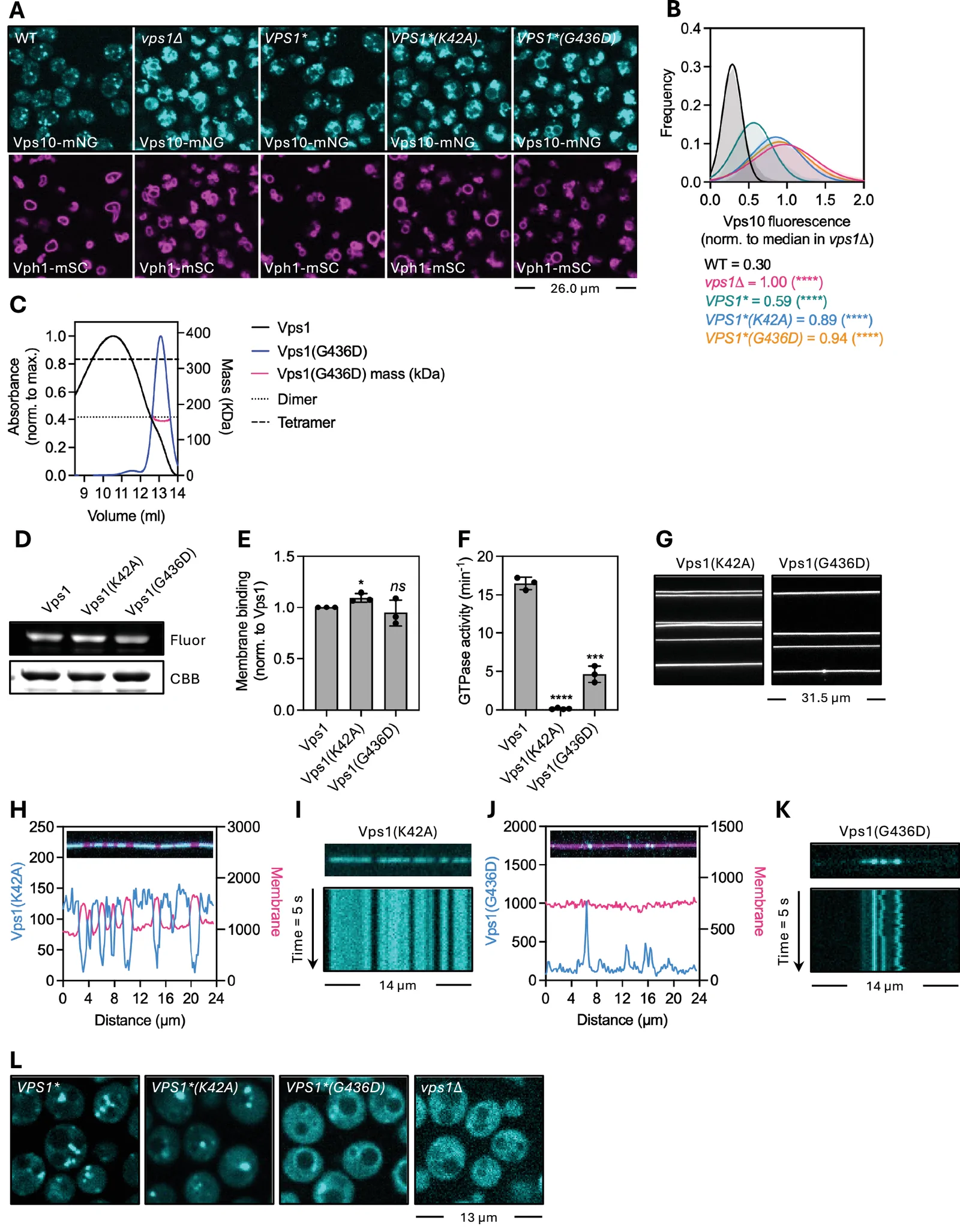
Vps1-catalyzed fission is required for Vps10 trafficking. (**A**) Representative fluorescence images displaying Vps10-mNG (cyan) and Vph1-mSc (magenta) in wild-type (WT), *vps1Δ*, VPS1*, VPS1*(K42A), and VPS1*(G436D) strains. (**B**) Quantitative distribution of vacuolar Vps10 in the indicated strains, shown as frequency distributions (shaded regions) fitted to Gaussian curves (solid lines). Data were normalized to Vps10-mNG levels in *vps1Δ* cells, with numbers representing median values. Data for each strain are derived from more than 10,000 Vph1-mSc-positive particles (*N* = 3 biological replicates), and statistical significance was determined against WT using an unpaired Welch’s *t*-test (****p < 0.0001). (**C**) SEC-MALS trace for the Vps1(G436D) mutant. (**D**) PLiMAP gel depicting the binding of Vps1 and its mutants to PC:PS:PI(3)P vesicles. (**E**) Normalized membrane binding of Vps1 and its mutants relative to Vps1 alone. Data were mean ± SD (*N* = 3 technical replicates), with statistical significance determined against Vps1 using an unpaired *t*-test (*p = 0.0016; ns not significant). (**F**) GTPase activity of Vps1 mutants in the presence of PC:PS:PI(3)P vesicles. Data were mean ± SD (*N* = 3 technical replicates), with statistical significance determined against Vps1 using an unpaired *t*-test (***p = 0.0001, ****p < 0.0001). (**G**) Representative images of SMrT tubes after a 10-minute exposure to Vps1 mutants in the presence of GTP. (**H**) Ax488-labeled Vps1(K42A) on a single tube in the presence of GppNHp, accompanied by the corresponding fluorescence line profile. (**I**) Representative kymograph from a time-lapse movie depicting Ax488-labeled Vps1(K42A) on a single tube in the presence of GppNHp. (**J**) Ax488-labeled Vps1(G436D) in the presence of GppNHp and its associated fluorescence line profile. (**K**) Representative kymograph from a time-lapse movie of Ax488-labeled Vps1(G436D) on a single tube in the presence of GppNHp. (**L**) Cellular distribution of ALFA-Nanobody-mNG in yeast strains expressing the indicated ALFA-tagged VPS1 constructs. Source data are available online for this figure.

Vps10 continuously cycles between endosomes and the trans-Golgi network (TGN) via a retrieval pathway that strictly depends on both Vps1 and the retromer complex. Consequently, the genetic ablation of VPS1 causes the mis-sorting of Vps10 to the vacuole, providing a robust readout to assay endosomal retrieval efficiency (Marcusson et al, 1994; Nothwehr et al, 1995; Cooper and Stevens, 1996; Arlt et al, 2014; Chi et al, 2014). To track this pathway, we endogenously tagged Vps10 with mNeonGreen (Vps10-mNG) and the a-subunit of the vacuolar V-ATPase V0 domain, Vph1, with mScarlet (Vph1-mSc). Confocal spinning-disk microscopy of wild-type (WT) cells revealed that Vps10-mNG localized to discrete puncta, reflecting its steady-state distribution at the TGN and endosomes. While these puncta moved dynamically throughout the cytoplasm, a distinct subset remained tethered to the vacuolar boundary marked by Vph1-mSc (Fig. 3A; Movie EV4). In contrast, *vps1Δ* cells exhibited a striking redistribution of Vps10-mNG to the vacuole, confirming severe cargo mis-sorting (Fig. 3A; Movie EV5). To analyze Vps1 functional domains, we implemented a genetic rescue strategy by integrating a modified VPS1* transgene at its native locus in the *vps1Δ* background. This transgene carries the same affinity tags utilized in our cell-free reconstitution assays, along with a 13-residue C-terminal ALFA tag to track protein distribution. In VPS1* cells, Vps10-mNG distribution reverted to a punctate, non-vacuolar pattern reminiscent of WT cells, demonstrating functional complementation of the retrieval defect (Fig. 3A; Movie EV6). To quantify this rescue, we developed an automated image analysis pipeline that measures the intensity of the Vps10-mNG signal within a mask defined by the Vph1-mSc vacuolar boundary. Population-level quantification confirmed significantly elevated vacuolar accumulation of Vps10-mNG in *vps1Δ* cells compared to WT cells. In VPS1* cells, vacuolar Vps10-mNG levels were markedly reduced and approached WT baselines (Fig. 3B). The slight deficit in complete rescue is likely an artifact of the bulky C-terminal ALFA tag on the VPS1* construct. Together, these results validate a robust, quantitative vacuolar localization assay to evaluate Vps1 mutants in vivo.

**Figure EV2:**
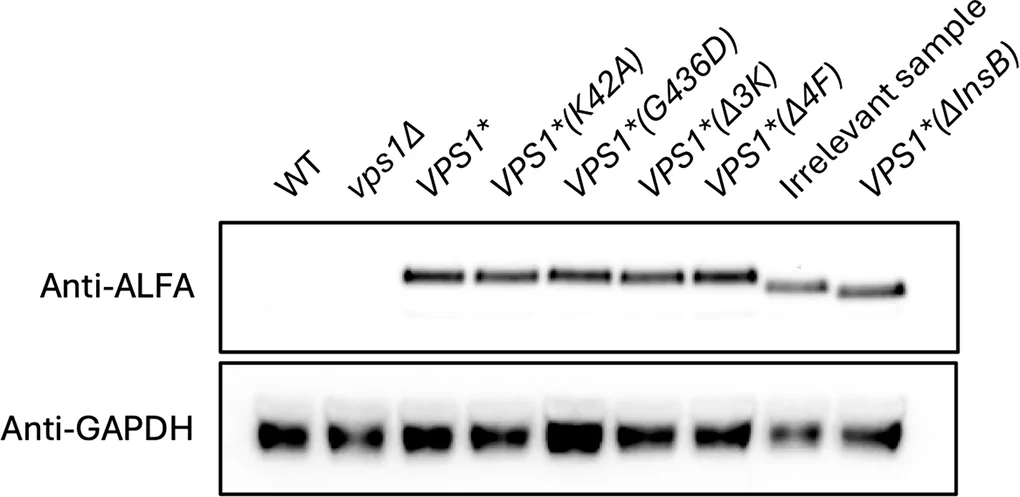
Western blots of Vps1 and its mutants. Lysates from the indicated strains were blotted with the anti-ALFA antibody. GAPDH served as the loading control. Source data are available online for this figure.

We next selected two well-characterized Vps1 functional mutants: K42A, which blocks GTP hydrolysis, and G436D, which impairs self-assembly (Varlakhanova et al, 2018). Western blot analysis confirmed that both mutant proteins were expressed at levels comparable to the Vps1* control (Fig. EV2). Strikingly, neither VPS1*(K42A) nor VPS1*(G436D) rescued the retrieval defect, as Vps10-mNG remained stuck in the vacuole both qualitatively (Fig. 3A; Movies EV7 and EV8) and quantitatively (Fig. 3B). To map these cellular phenotypes directly to their biochemistry, we subjected the mutant proteins to in vitro analysis. Size-exclusion chromatography coupled with multi-angle light scattering (SEC-MALS) confirmed that Vps1(G436D) exists primarily as a stable dimer (Fig. 3C), aligning with previous structural predictions (Varlakhanova et al, 2018). Conversely, wild-type Vps1 yielded a broad, unresolved high-molecular-weight peak, reflecting its inherent propensity to self-assemble into higher-order oligomers in solution. PLiMAP assays revealed that both Vps1(K42A) and Vps1(G436D) retained membrane-binding affinities for PC:PS:PI(3)P vesicles that were indistinguishable from wild-type Vps1 (Fig. 3D,E). However, their enzymatic activities differed drastically in presence of vesicles; Vps1(K42A) displayed negligible stimulated GTPase activity, whereas Vps1(G436D) exhibited a turnover rate of 4.5 min^−^¹— a 3.5-fold reduction compared to WT (Fig. 3F). Crucially, both mutants were completely inactive in SMrT tubule fission assays (Fig. 3G), proving that baseline membrane binding is insufficient for mechanical work without self-assembly and accelerated GTP hydrolysis. When perfused onto SMrT templates, Vps1(K42A) organized into long, highly stable structural scaffolds that constricted the underlying lipid tubules (Fig. 3H,I), like WT (Fig. 2E). In sharp contrast, Vps1(G436D) formed small, disorganized foci that drifted laterally along the tubule surface and failed to induce any membrane constriction (Fig. 3J,K). This behavior highlights a significant technical insight: while Vps1(G436D) appeared normal in PLiMAP vesicle binding assays, it exhibited severe defects on SMrT templates. This discrepancy stems from the distinct sensitivities of the two methods. Unlike bulk equilibrium assays like PLiMAP, imaging-based SMrT assays are highly sensitive to avidity arising from cooperative binding. Because scaffolded polymers rely on multivalent interactions, self-assembly defects severely diminish this avidity. Under flow conditions where total lipid surface area is restricted, and continuous washing removes weakly associated species, assembly defects become readily apparent. Utilizing both approaches in tandem thus provides a powerful diagnostic framework to distinguish primary lipid-binding defects from secondary self-assembly defects.

### Distribution and dynamics of Vps1 in cells

**Figure EV3:**
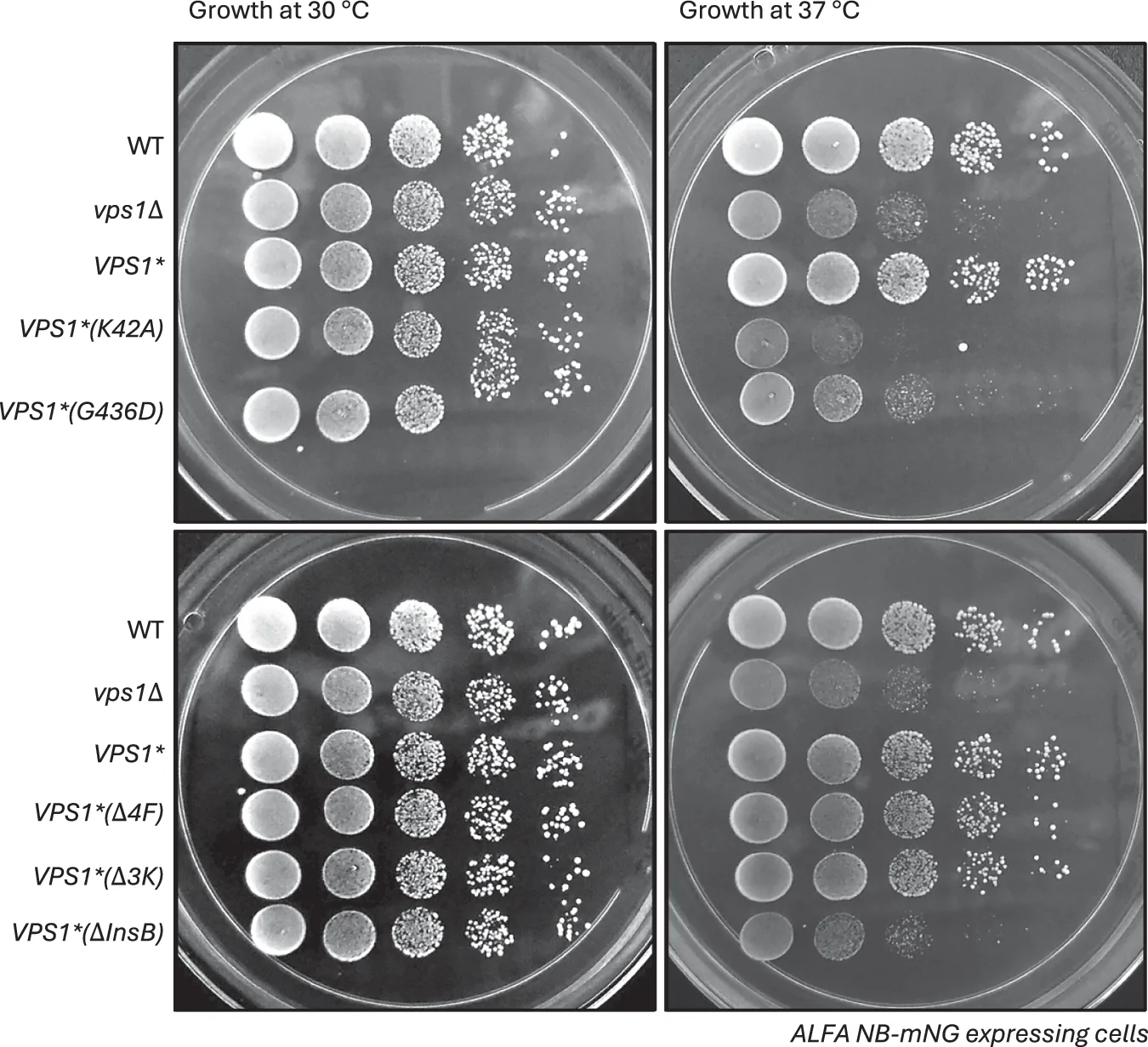
Growth characteristics of VPS1* strains. Spot assay comparing the growth of the indicated strains expressing the ALFA-NB-mNG at 30 and 37 °C.

To visualize Vps1 localization and dynamics in vivo without compromising its function, we employed the ALIBY (ALFA-tagged protein imaging by an anti-ALFA-nanobody) strategy, co-expressing the C-terminally ALFA-tagged Vps1 construct with an ALFA-nanobody fused to mNeonGreen (ALFA-NB-mNG) (Götzke et al, 2019; Akhuli et al, 2022). This indirect labeling approach was chosen because conventional, direct C-terminal tagging of Vps1 with green fluorescent protein (GFP) variants has been documented to disrupt its native functions (Suzuki et al, 2021; Arlt et al, 2023). While *vps1Δ* cells exhibit characteristic temperature-sensitive growth defects at elevated temperatures (Rothman et al, 1990), VPS1* cells co-expressing the ALFA-NB-mNG grew normally. This confirms that the binding of the nanobody to the tag does not further compromise Vps1 function (Fig. EV3). In contrast, both the VPS1*(K42A) and VPS1*(G436D) strains displayed severe growth defects at high temperatures, mirroring the *vps1Δ* phenotype (Fig. EV3). Confocal spinning-disk microscopy revealed that the ALFA-NB-mNG signal marked numerous highly dynamic puncta throughout the cytoplasm of VPS1* cells (Fig. 3L; Movie EV9). Conversely, in VPS1*(K42A) cells, the signal coalesced into a small number of abnormally large, stable clusters (Fig. 3L; Movie EV10). In VPS1*(G436D) cells, the fluorescent signal was uniformly diffuse throughout the cytoplasm (Fig. 3L; Movie EV11), closely resembling the background pattern observed in control *vps1Δ* cells lacking an ALFA-tagged bait protein (Fig. 3L; Movie EV12). Mechanistically, the large, static foci observed with Vps1(K42A) likely represent hyper-stabilized, higher-order self-assemblies that are trapped in a constitutive GTP-bound state due to a failure in hydrolysis. In sharp contrast, the diffuse cytoplasmic distribution of Vps1(G436D) confirms that structural dimeric mutants fail to undergo self-assembly in vivo. Taken together, these cellular data demonstrate that the small, transient puncta observed under wild-type conditions capture the normal physiological steady state of continuous Vps1 self-assembly and rapid, GTPase-driven disassembly.

### Functional analysis of Insert B in Vps1

Classic endocytic dynamins engage with membrane phosphoinositides through a structured pleckstrin homology domain (PHD), which serves as a membrane-binding foot (Khurana and Pucadyil, 2023). However, several members of the dynamin superfamily naturally lack a PHD, relying instead on alternative, specialized regions for membrane interaction. These include the Paddle Domain (PD) in BDLP and Opa1, the variable domain (VD) in Drp1, the L4 loop in MxA, and Insert B (InsB) in Vps1 (Jimah and Hinshaw, 2019). Although InsB has been reported to interact broadly with lipids (Smaczynska-de Rooij et al, 2019), a comprehensive mechanochemical understanding of how this region orchestrates Vps1 function remains elusive. We therefore systematically analyzed Vps1 constructs bearing mutations in distinct regions of InsB using our integrated suite of in vitro and in vivo assays.

**Figure 4:**
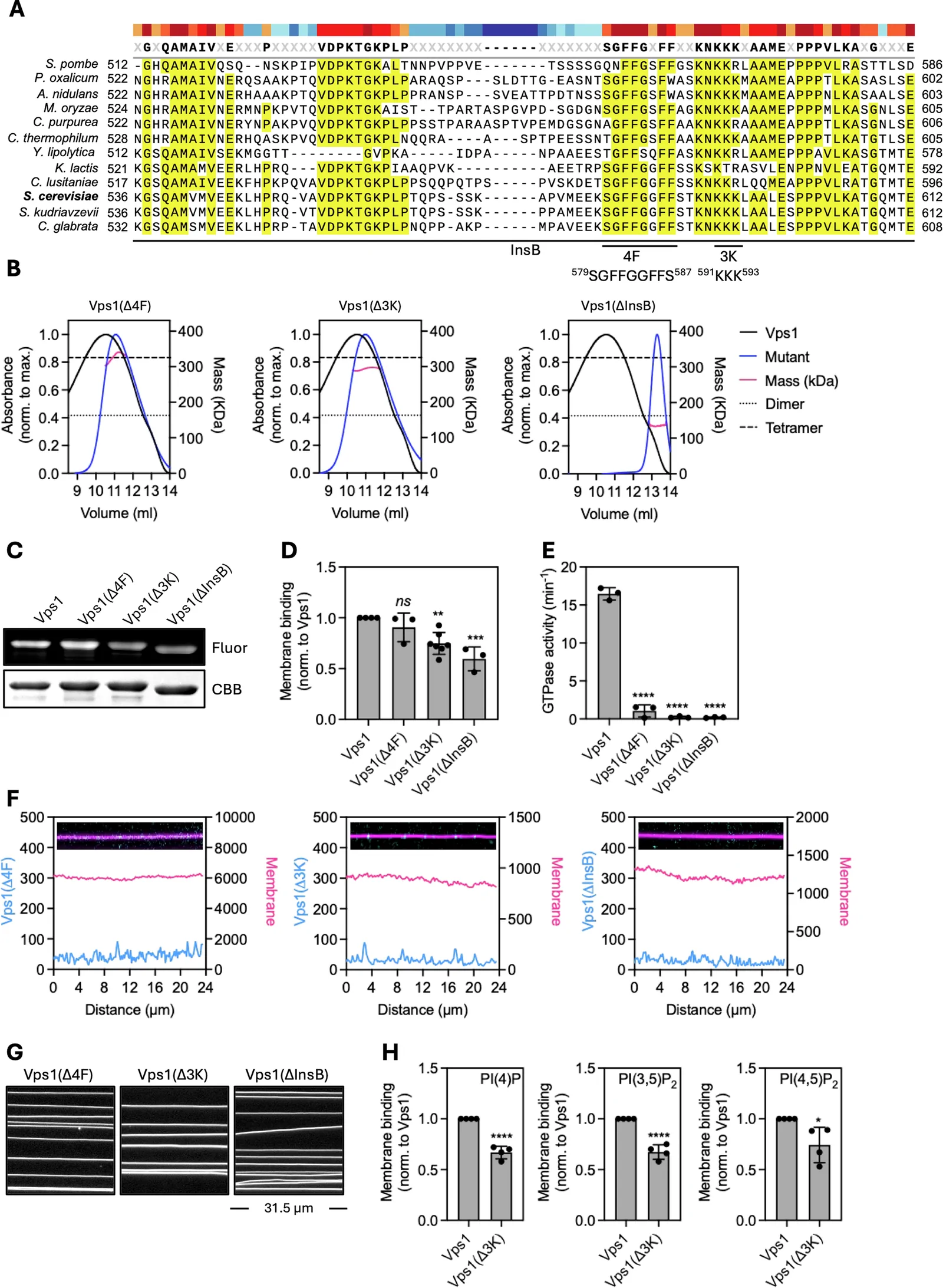
Insert B is required for Vps1-catalyzed membrane fission. (**A**) Sequence alignment of InsB from various fungi, highlighting the amino acid sequences of the 4F and 3K motifs in *S. cerevisiae*. (**B**) SEC-MALS trace for Vps1 mutants. (**C**) PLiMAP gel depicting the binding of InsB mutants to PC:PS:PI(3)P vesicles. (**D**) Normalized membrane binding of Vps1 and its mutants relative to WT. Data were mean ± SD (*N* ≥ 3 technical replicates), with statistical significance determined against Vps1 using an unpaired *t*-test (ns not significant; **p = 0.0013; ***p = 0.0008). (**E**) GTPase activity of Vps1 mutants in the presence of PC:PS:PI(3)P vesicles. Data are mean ± SD (*N* = 3 technical replicates), with statistical significance determined against Vps1 using an unpaired *t*-test (****p < 0.0001). (**F**) Ax488-labeled Vps1 mutants in the presence of GppNHp with corresponding fluorescence line profiles. (**G**) Representative images of SMrTs after a 10-min exposure to the indicated Vps1 mutants in the presence of GTP. (**H**) Membrane binding of Vps1 and Vps1(Δ3K) normalized to the signal obtained with Vps1 on PC:PS vesicles containing 5 mol% of the indicated phosphoinositide. Data represent the mean ± SD (*N* ≥ 4 technical replicates), with statistical significance determined against Vps1 using an unpaired *t*-test (*p = 0.0254, ****p < 0.0001). Source data are available online for this figure.

InsB encompasses residues 536–612 in *S. cerevisiae* Vps1, and sequence alignment across fungal orthologs reveals several highly conserved stretches (Fig. 4A). Notably, the C-terminal region features a phenylalanine-rich “4F” motif (residues 579–587) and a lysine-rich “3K” motif (residues 591–593). Given that aromatic and positively charged residues frequently coordinate to drive peripheral membrane binding, we generated targeted deletion constructs lacking the 4F motif (Δ4F) or the 3K motif (Δ3K), alongside a complete deletion of the entire InsB region replaced by a short, flexible linker (ΔInsB). SEC-MALS analysis revealed that both Vps1(Δ4F) and Vps1(Δ3K) exist predominantly as tetramers in solution (Fig. 4B). However, both mutants eluted slightly later and displayed narrower profile peaks than WT. This indicates that WT Vps1 exists in a rapidly interconverting equilibrium between tetramers and higher-order oligomers, a process to which the 4F and 3K motifs partially contribute. In contrast, Vps1(ΔInsB) migrated strictly as a stable dimer (Fig. 4B). This structural shift indicates that regions flanking the 4F and 3K motifs within InsB facilitate crucial inter-subunit interactions required to maintain the baseline tetrameric state of Vps1. Interestingly, deletion of the structurally equivalent variable domain (VD) similarly converts the mitochondrial dynamin Drp1 into a dimeric species (Clinton et al, 2016), highlighting an evolutionarily conserved role for these unstructured inserts in stabilizing the quaternary architecture of dynamins.

PLiMAP tracking demonstrated that Vps1(Δ4F) retained membrane-binding characteristics on bulk PC:PS:PI(3)P vesicles that were indistinguishable from WT (Fig. 4C,D). However, this mutant exhibited a severe defect in assembly-stimulated GTPase activity (Fig. 4E). When visualized on SMrT membrane tubules, fluorescently labeled Vps1(Δ4 F) displayed drastically lower membrane density (Fig. 4F), mimicking the phenotype observed with the self-assembly-deficient dimeric mutant Vps1(G436D) (Fig. 3J,K). Intriguingly, the magnitude of this structural defect on tubules was considerably more pronounced in Vps1(Δ4F) than in Vps1(G436D), even though Δ4F remains a tetramer in bulk solution, whereas G436D is a stable dimer (Figs. 3C and 4B). These findings suggest that the 4F motif acts as a potent, membrane-dependent catalyst for higher-order helical polymerization. Conversely, the Vps1(Δ3K) mutant exhibited a significant reduction in membrane binding (Fig. 4C,D), confirming that the 3K cluster contributes directly to phosphoinositide binding. Consequently, Vps1(Δ3K) was severely compromised in assembly-stimulated GTPase activity on PC:PS:PI(3)P vesicles (Fig. 4E) and failed to assemble on SMrT tubules (Fig. 4F). The complete removal of the insert in Vps1(ΔInsB) caused a further drop in binding compared to the Δ3K baseline (Fig. 4D), indicating the presence of auxiliary, lower-affinity lipid-binding sites throughout InsB. Lacking both the 4F and 3K motifs and constrained by its dimeric nature, Vps1(ΔInsB) was incapable of membrane binding (Fig. 4F) and displayed a near-complete loss of stimulated GTPase activity (Fig. 4E). Owing to these profound biochemical defects, Δ4F, Δ3K, and ΔInsB were entirely devoid of mechanical tubule fission activity (Fig. 4G). Previous studies using bulk vesicle sedimentation assays reported that the 3K motif is essential for binding anionic lipids and certain phosphoinositides (Smaczynska-de Rooij et al, 2019). Specifically, replacing the 3K cluster with alanines (3A) impaired binding to general anionic lipids and PI(4,5)P₂ without affecting PI(4)P binding, yet unexpectedly appeared to enhance binding to PI(3)P relative to WT Vps1. In contrast, our sensitive PLiMAP assays demonstrate that loss of the 3K motif directly reduces PI(3)P association (Fig. 4D). Given that Vps1 displays broad promiscuity across various phosphoinositide species (Fig. 1K), we tested whether the 3K defect was unique to endosomal lipids. Vps1(Δ3K) proved uniformly defective in binding to all other phosphoinositides tested (Fig. 4H), confirming that the 3K cluster represents a generic, non-specific phosphoinositide-binding motif. Finally, we evaluated these mutants for their cellular distribution and performance in our in vivo vacuolar trafficking assay. Western blotting confirmed that all three mutant proteins were expressed at physiological levels equivalent to the VPS1* control (Fig. EV2). Unexpectedly, VPS1*(Δ4F) and VPS1*(Δ3K) cells—but not VPS1*(ΔInsB) cells—grew robustly at elevated temperatures, indicating that the individual Δ4F and Δ3K mutants retain partial physiological activity (Fig. EV3).

**Figure 5:**
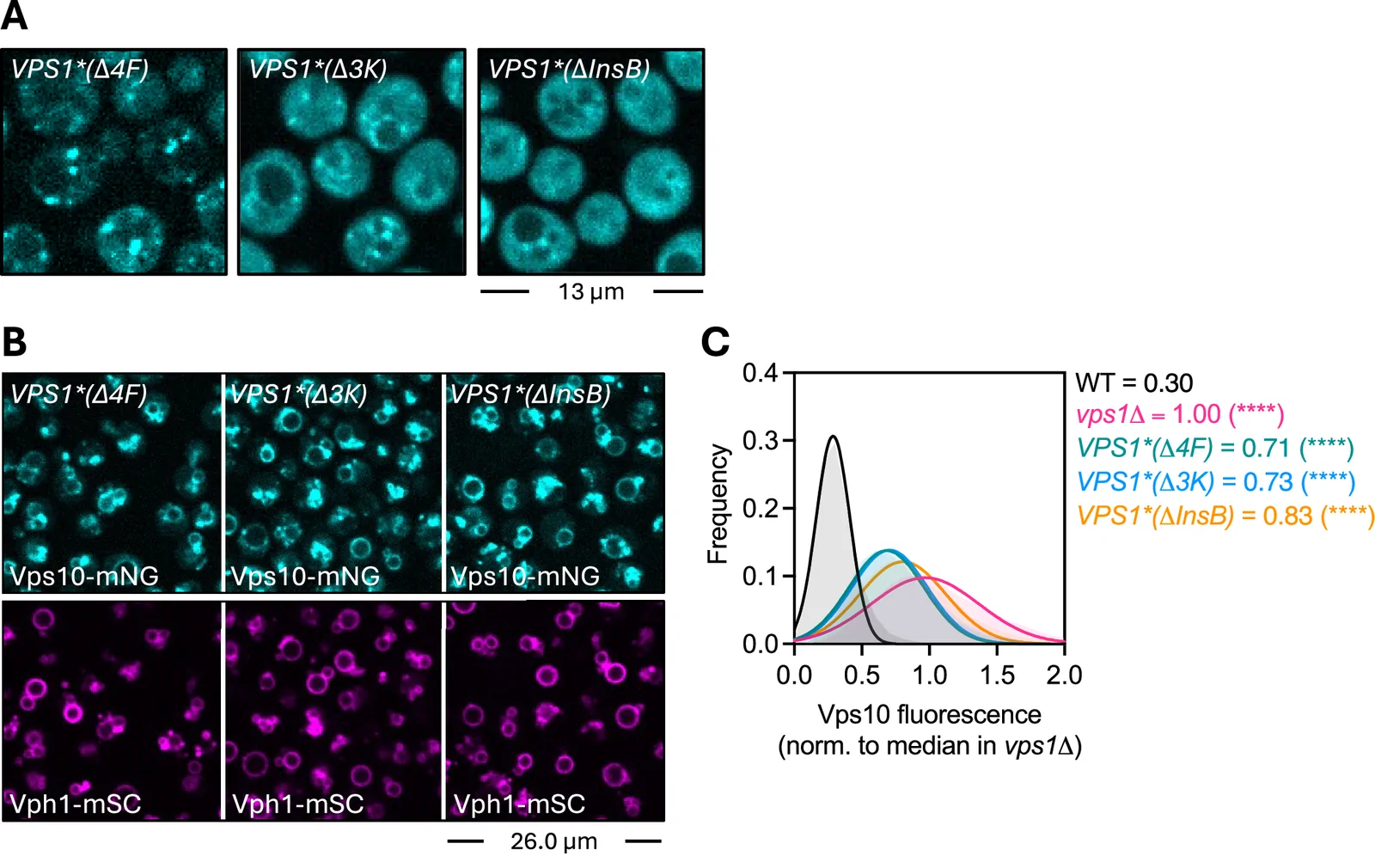
Insert B is required for Vps10 trafficking. (**A**) Cellular distribution of ALFA-Nanobody-mNG in yeast strains expressing the indicated ALFA-tagged VPS1 constructs. (**B**) Representative fluorescence images showing Vps10-mNG (cyan) and Vph1-mSc (magenta) localization in the indicated strains. (**C**) Quantitative distribution of vacuolar Vps10 in the indicated strains, determined by measuring Vps10-mNG fluorescence in Vph1-mSc-positive particles. Data are shown as frequency distributions (shaded regions) fitted to Gaussian curves (solid lines) and are normalized to Vps10-mNG levels in *vps1Δ* cells, with median values indicated. Data for each strain are derived from more than 10,000 Vph1-mSc-positive particles across three biological replicates, and statistical significance was determined against WT using an unpaired Welch’s *t*-test (****p < 0.0001). Source data are available online for this figure.

Visualizing these strains via the ALIBY imaging pipeline revealed that Vps1(Δ4F) formed fewer, less prominent dynamic foci than the wild-type control (Fig. 5A; Movie EV13). Meanwhile, both Vps1(Δ3K) (Fig. 5A; Movie EV14) and Vps1(ΔInsB) (Fig. 5A; Movie EV15) exhibited completely diffuse cytoplasmic distributions. Crucially, all three mutant strains showed a significant accumulation of Vps10-mNG within the vacuole (Fig. 5B; Movies EV16–EV18). Population-level quantification confirmed that all three mutants failed to functionally complement the *vps1Δ* cargo-sorting defect (Fig. 5C). The magnitude of this trafficking defect was considerably more severe in VPS1*(ΔInsB) than in either VPS1*(Δ4F) or VPS1*(Δ3K) cells (Fig. 5C).

Taken together, these data successfully dissect and assign distinct biophysical roles to the highly conserved modules within Insert B: the phenylalanine-rich 4F motif accelerates membrane-dependent helical self-assembly, whereas the basic 3K motif mediates generic phosphoinositide docking. Coordinated action across both modules is strictly required for Vps1-mediated membrane fission and endosomal protein sorting.

### Quantitative vacuolar proteomics reveal the repertoire of cargo trafficked via Vps1-dependent pathways

Our findings directly couple Vps1-mediated membrane fission to the endosomal retrieval of the cargo receptor Vps10. Because membrane fission is a fundamental prerequisite for transport carrier generation, we hypothesized that the loss of Vps1 would universally disrupt the trafficking of various other cargo proteins. Given that *vps1Δ* cells aberrantly mis-sort Vps10 to the vacuole, and because yeast vacuoles can be isolated with high purity, we subjected the *vps1Δ* vacuolar proteome to quantitative comparative proteomics to globally define the landscape of proteins dependent on Vps1 for their intracellular sorting.

**Figure EV4:**
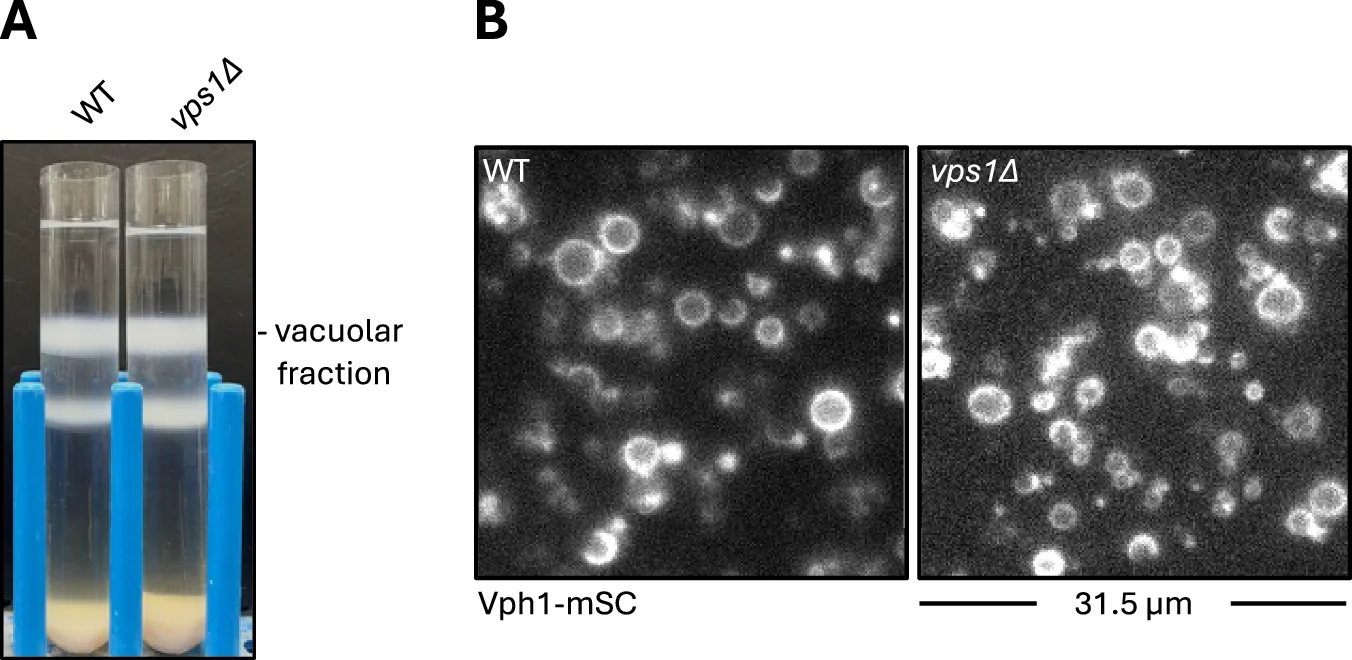
Characteristics of vacuoles isolated from WT and *vps1Δ* strains. (**A**) Density-gradient centrifugation-based separation of lysates from WT and *vps1Δ* cells, indicating the isolated vacuolar fraction. (**B**) Purified vacuoles from WT and *vps1Δ* cells expressing the vacuole marker Vph1-mSc.

**Figure 6:**
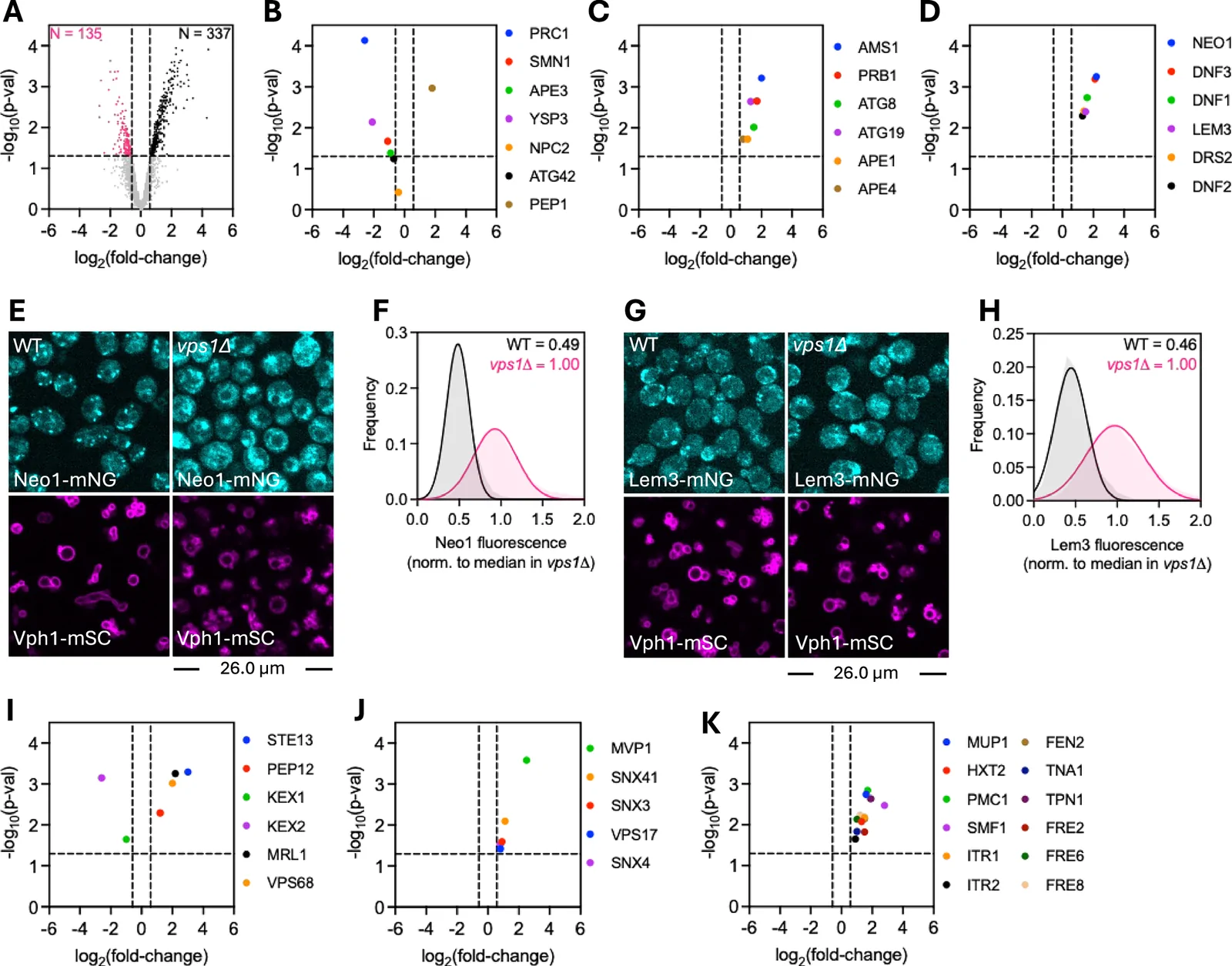
Quantitative proteomics of vacuoles from *vps1Δ* cells. (**A**) Volcano plot displaying enriched (black) and depleted (magenta) proteins in vacuoles isolated from *vps1Δ* cells compared to WT. Dashed lines denote cut-off thresholds for fold change (1.5-fold) and statistical significance (*p* = 0.05, two-tailed Student’s *t*-test). (**B**) Fold change in the levels of PEP1(VPS10) and its associated cargo in *vps1Δ* versus WT vacuoles. Dashed lines denote cut-off thresholds for fold change (1.5-fold) and statistical significance (*p* = 0.05, two-tailed Student’s *t*-test). (**C**) Fold change in the levels of proteins involved in the cytoplasm-to-vacuole targeting (CVT) pathway in *vps1Δ* versus WT vacuoles. Dashed lines denote cut-off thresholds for fold change (1.5-fold) and statistical significance (*p* = 0.05, two-tailed Student’s *t*-test). (**D**) Fold change in the levels of some proteins annotated with Golgi or endosome localization within the vacuolar fraction in *vps1Δ* versus WT vacuoles. Dashed lines denote cut-off thresholds for fold change (1.5-fold) and statistical significance (*p* = 0.05, two-tailed Student’s *t*-test). (**E**) Representative fluorescence images showing Neo1-mNG (cyan) and Vph1-mSc (magenta) in WT and *vps1Δ* cells. (**F**) Quantitative distribution of vacuolar Neo1 in WT and *vps1Δ* cells, determined by measuring Neo1-mNG fluorescence in Vph1-mSc-positive particles. Data are frequency distributions (shaded regions) fitted to Gaussian curves (solid lines) and are normalized to Neo1-mNG levels in *vps1Δ* cells, with median values indicated. Data for each strain are derived from more than 10,000 Vph1-mSc-positive particles across three biological replicates. (**G**) Representative fluorescence images showing Lem3-mNG (cyan) and Vph1-mSc (magenta) in WT and *vps1Δ* cells. (**H**) Quantitative distribution of vacuolar Lem3 in WT and *vps1Δ* cells, determined by measuring Lem3-mNG fluorescence in Vph1-mSc-positive particles. Data are frequency distributions (shaded regions) fitted to Gaussian curves (solid lines) and are normalized to Lem3-mNG levels in *vps1Δ* cells, with median values indicated. Data for each strain are derived from more than 10,000 Vph1-mSc-positive particles across three biological replicates. (**I**) Fold change in the levels of cargo trafficked by retromer-dependent and -independent pathways utilizing the SNX and SNX-BAR proteins in *vps1Δ* versus WT vacuoles. Dashed lines denote cut-off thresholds for fold change (1.5-fold) and statistical significance (*p* = 0.05, two-tailed Student’s *t*-test). (**J**) Fold change in the levels of SNX and SNX-BAR proteins in *vps1Δ* versus WT vacuoles. Dashed lines denote cut-off thresholds for fold change (1.5-fold) and statistical significance (*p* = 0.05, two-tailed Student’s *t*-test). (**K**) Fold change in the levels of vacuolar proteins in *vps1Δ* versus WT vacuoles, highlighting proteins that are also localized to the plasma membrane. Dashed lines denote cut-off thresholds for fold change (1.5-fold) and statistical significance (*p* = 0.05, two-tailed Student’s *t*-test). Source data are available online for this figure.

Vacuoles isolated via density-gradient centrifugation from *vps1Δ* cells exhibited a banding pattern and gross morphology indistinguishable from those of wild-type (WT) cells (Fig. EV4), permitting unbiased quantitative comparisons. Data-independent acquisition (DIA) mass spectrometry identified a total of 3703 proteins (Dataset EV1). Using a filtering threshold of a 1.5-fold change in abundance and a significance threshold of *p* < 0.05, we identified 337 enriched and 135 depleted proteins in *vps1Δ* vacuoles relative to WT controls (Fig. 6A; Dataset EV2). Notably, Pep1 (Vps10) was significantly enriched, whereas its soluble luminal cargo Prc1 (carboxypeptidase Y, CPY) was the most depleted protein within *vps1Δ* vacuoles (Fig. 6B; Dataset EV3). Other soluble vacuolar hydrolases whose delivery relies on Vps10-mediated cycling—such as Smn1 and Ape3—were similarly depleted (Fig. 6B; Dataset EV3), alongside other cargoes like Atg42 and Npc2 (Fig. 6B; Dataset EV3). Together, these proteomic signatures strongly corroborate classical vacuolar protein sorting models (Marcusson et al, 1994; Nothwehr et al, 1995; Cooper and Stevens, 1996; Arlt et al, 2014; Chi et al, 2014; Eising et al, 2022). Conversely, *vps1Δ* vacuoles displayed marked enrichment for components of the cytoplasm-to-vacuole targeting (CVT) pathway (Ape1, Ams1, Atg8, and Atg19) alongside the proteases Prb1 and Ape4 (Fig. 6C; Dataset EV4). This distinctive accumulation profile mirrors phenotypes observed in *vps10Δ* mutants (Eising et al, 2022), likely representing a coordinated, global adaptive response to preserve vacuolar lytic capacity amidst sorting failures. Because loss of Vps1 causes the vacuolar accumulation of the endosome- and Golgi-localized receptor Vps10, we evaluated whether other membrane proteins resident within these compartments shared a similar fate. Indeed, a large cohort of trans-Golgi network (TGN) and endosomal membrane proteins were significantly enriched in *vps1Δ* vacuoles, most notably the phospholipid flippases Neo1, Drs2, and Dnf1/2/3 (Fig. 6D; Dataset EV5). Live-cell imaging validated these findings; Neo1-mNG localized to discrete intracellular puncta in WT cells but was significantly mis-sorted to the vacuole in *vps1Δ* cells (Fig. 6E,F; Movies EV19 and EV20). Neo1 and its mammalian ortholog ATP9A require the SNX3-dependent retromer pathway for recycling (Dalton et al, 2017; McGough et al, 2018; Suzuki et al, 2021); thus, its vacuolar accumulation implies a strict mechanical requirement for Vps1 in this specific retrieval arm. Similarly, the flippases Dnf1/2 undergo retromer-mediated endosome-to-TGN trafficking as part of a functional complex with Lem3 (Bai et al, 2020; Jiménez et al, 2024). Our proteomic analysis revealed that Lem3 was concurrently enriched in *vps1Δ* vacuoles. Under microscopy, Lem3-mNG shifted from faint puncta in WT cells to a diffuse distribution in *vps1Δ* cells (Fig. 6G; Movies EV21 and EV22), with our vacuolar localization assay confirming its marked accumulation in mutant vacuoles (Fig. 6H). We also observed vacuolar enrichment of Ste13 and Pep12 (Fig. 6I; Dataset EV5), both well-characterized cargoes of the SNX3-retromer pathway (Hettema et al, 2003; Bonifacino and Hurley, 2008; Purushothaman and Ungermann, 2018). Paradoxically, other established SNX3-retromer cargoes, such as Kex1 and Kex2, were among the most severely depleted proteins in *vps1Δ* vacuoles (Fig. 6I and Dataset EV5) (Voos and Stevens, 1998; Burda et al, 2002); the mechanistic basis driving this cargo-specific differential sorting requires further investigation. Beyond retromer pathways, Vps1 interacts with Mvp1 to mediate a retromer-independent retrograde pathway targeting cargo like Vps55 (Suzuki et al, 2021). While Vps55 evaded mass spectrometry detection, its known binding partner Vps68 was robustly enriched in *vps1Δ* vacuoles (Fig. 6I; Dataset EV5), positioning Vps1 as a core component of the Mvp1 pathway (Suzuki et al, 2021). Furthermore, *vps1Δ* vacuoles were enriched for Atg27 (Fig. 6I; Dataset EV5), a cargo retrieved via the SNX4/41/42 pathway (Ma et al, 2017b), as well as Mrl1 (Fig. 6I; Dataset EV5)—a homolog of the mammalian mannose-6-phosphate receptor and cargo of the endosomal VINE sorting complex (Shortill et al, 2022a; Shortill et al, 2022b). The broad, shared accumulation profile of these Golgi and endosomal residents suggests they represent diverse retrieval cargoes that rely on Vps1-mediated fission to escape endosomes. Mechanistically, receptor retrieval from endosomes back to the Golgi requires cargo-specific adapters cooperating with membrane-tubulating SNX-BAR coats (Shortill et al, 2022a). Blocking Vps1-mediated fission should theoretically stall transport carrier release, trapping and enriching these SNX-BAR assemblies on the donor membrane. In agreement with this model, the SNX-BAR proteins Vps17, Snx4/41, and Mvp1, along with the retromer-associated PX-domain protein Snx3, were significantly enriched within the *vps1Δ* vacuolar fraction (Fig. 6J; Dataset EV6). Intriguingly, the *vps1Δ* vacuolar proteome was also heavily enriched for numerous proteins native to the plasma membrane (Fig. 6K; Dataset EV7). This cohort included nutrient transporters and permeases (Mup1, Hxt2, Pmc1, Smf1, Itr1/2, Fen2, Tna1, Tpn1, and Fre2/6/8) that undergo ubiquitination-dependent degradation via the multivesicular body (MVB) pathway under nutrient-replete conditions. Prior work on the reductive iron transporters Fet3 and Ftr1 established that degradation via the MVB pathway directly competes with retromer-mediated recycling (Strochlic et al, 2008). Thus, a failure in endosomal recycling in *vps1Δ* cells likely diverts a larger, unretrieved pool of these cell surface proteins into the default MVB pathway, driving their accumulation in the vacuole. What fundamental mechanism accounts for the vacuolar pooling of such a diverse array of Golgi, endosomal, and plasma membrane proteins in the absence of Vps1? While Vps10 is known to be re-routed through the secretory pathway to the plasma membrane in *vps1Δ* cells before being internalized via endocytosis (Nothwehr et al, 1995), this circuitous routing alone does not clarify its enrichment inside the vacuole. Vesicle fusion between the Golgi and endosomes is mediated by the SNARE complex comprising Pep12 (Qa), Vti1 (Qb), Syn8 (Qc), and Nyv1/Ykt6 (R), whereas endosome-vacuole fusion relies on Vam3 (Qa), Vti1 (Qb), Vam8 (Qc, and Nyv1/Ykt6 (R) (Burri and Lithgow, 2004). Crucially, overexpression of the endosomal SNARE Pep12 can functionally bypass the loss of Vam3, the primary SNARE driving endosome-vacuole fusion (Darsow et al, 1997). Our proteomic dataset revealed that both Pep12 and its core interactor Syn8 are significantly enriched in *vps1Δ* vacuoles (Dataset EV8) (Lewis and Pelham, 2002). Because Pep12 is an established cargo of the SNX3-retromer pathway (Hettema et al, 2003; Purushothaman and Ungermann, 2018), its impaired recycling likely stalls it at the endosome/pre-vacuolar compartment (PVC). The resulting hyper-accumulation of Pep12 on these endosomal compartments likely triggers aberrant, direct fusion with the vacuole, driving the bulk delivery of upstream Golgi and endocytic cargoes into the vacuolar lumen.

**Figure 7:**
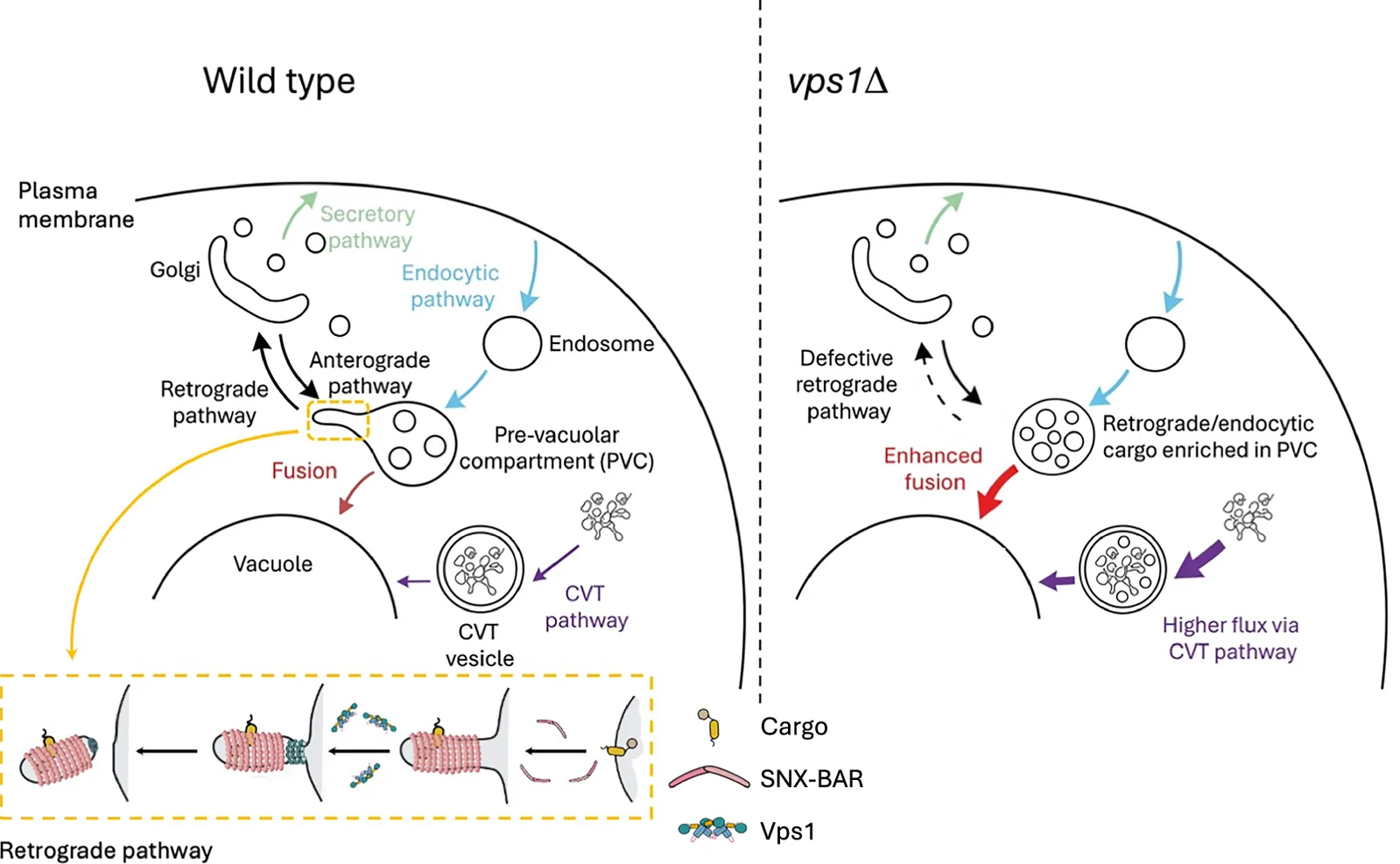
Proposed model for Vps1-mediated trafficking. In wild-type (WT) cells, the pre-vacuolar compartment (PVC; depicted to include multivesicular bodies) acts as a central sorting hub. The PVC receives anterograde cargo from the Golgi and endocytic cargo from the plasma membrane, a portion of which is recycled. Retrograde trafficking retrieves cargo from the PVC back to the Golgi via retromer-dependent and -independent pathways. This retrieval process requires cargo-specific adapters, SNX-BAR-mediated membrane tubulation, and Vps1-mediated fission. Eventually, the PVC fuses with the vacuole to deliver its remaining contents, while the cytoplasm-to-vacuole targeting (CVT) pathway transports proteins directly to the vacuole. In *vps1Δ* cells, defective retrograde fission blocks cargo retrieval, causing the accumulation of retrograde, endocytic, and SNARE cargo within the PVC. This SNARE accumulation enhances PVC-vacuole fusion, while the overall trafficking block triggers compensatory upregulation of the CVT pathway to maintain vacuolar function.

Based on these findings, we propose a model describing our experimental data (Fig. 7). The pre-vacuolar compartment (PVC)—which, for simplicity, is illustrated to include multivesicular bodies—serves as a central hub where cargo from multiple pathways converges and is sorted. Anterograde traffic delivers cargo from the Golgi to the PVC, whereas retrograde traffic operates in reverse, retrieving cargo from the PVC back to the Golgi. The PVC also receives cargo via endocytic trafficking, a portion of which can be recycled out of this compartment. Eventually, the PVC fuses with the vacuole to deliver its remaining contents. Concurrently, the yeast-specific cytoplasm-to-vacuole targeting (CVT) pathway transports proteins directly to the vacuole. Cargo retrieval from the PVC is mediated by retromer-dependent and retromer-independent pathways. These pathways rely on cargo-specific adapters for sorting, SNX-BAR proteins for membrane tubulation, and ultimately Vps1 for membrane fission. Consequently, the loss of Vps1 disrupts retrograde trafficking from the PVC to the Golgi, leading to an accumulation of the SNX-BAR proteins and both retrograde and endocytic cargo within the PVC. In response to these trafficking defects, the CVT pathway is upregulated. This upregulation likely reflects an adaptive mechanism to maintain vacuolar function, though its direct links to Vps1 remain unclear. The enrichment of endocytic cargo is explained by the failure of the defective retrograde arm to recycle these proteins out of the PVC. Furthermore, the accumulation of SNARE proteins in the vacuole—resulting from a failure in retrograde retrieval—likely triggers enhanced fusion between the PVC and the vacuole.

## Discussion

Using an integrated approach combining biochemistry, cell-free reconstitution, and live-cell imaging, we have comprehensively analyzed the mechanochemical functions of the yeast dynamin Vps1. Our findings demonstrate that Vps1 is fully sufficient to drive the constriction and subsequent fission of tubular lipid membranes. Given that Vps1 has been genetically and physically linked to other cellular processes requiring membrane remodeling—such as endocytosis, autophagy, and peroxisomal division (Kuravi et al, 2006; Rooij et al, 2010; Palmer et al, 2015; Arlt et al, 2023)—it is highly probable that Vps1 leverages this conserved, intrinsic fission activity across these diverse pathways.

From a membrane perspective, fission is tightly regulated by lipid composition and membrane topology, whereas on the protein level, it strictly requires the higher-order self-assembly of Vps1 into structural scaffolds. Our findings demonstrate that phosphoinositides stabilize Vps1 recruitment to the membrane. Prior structural and biochemical analyses on lipid nanotubes reported a strong propensity for Vps1 to self-assemble into scaffolds but unexpectedly revealed no significant assembly-stimulated GTPase activity (Varlakhanova et al, 2018). Interestingly, those reported rates are comparable to the moderate turnover we observe for *S. cerevisiae* Vps1 on PC:PS vesicles. In contrast, our current results uncover a robust ~30-fold stimulation in GTPase activity upon phosphoinositide engagement. While the precise cause for this discrepancy remains to be determined, it may reflect distinct intrinsic biophysical properties of the *C. thermophilum* Vps1 ortholog tested previously compared to the *S. cerevisiae* enzyme analyzed here. Mechanistically, Vps1 docks onto phosphoinositides through a conserved, lysine-rich (3K) motif located within Insert B (InsB). It then polymerizes into helical scaffolds around membrane tubules; the structural determinants for this self-assembly reside primarily within the stalk but are surprisingly also encoded within a conserved, phenylalanine-rich (4F) motif in InsB. GTP-bound Vps1 scaffolds are intrinsically membrane-active and constrict underlying tubules down to a fixed radius of ~20 nm. Cryo-EM reconstructions of soluble, nucleotide-bound Vps1 scaffolds revealed an inner lumen radius of ~10 nm (Varlakhanova et al, 2018); accounting for typical bilayer thickness, this inner dimension is significantly narrower than the ~20 nm outer tubule radius captured in our assays. This geometric mismatch implies that the lipid bilayer exerts mechanical resistance that acts as a barrier, limiting the full intrinsic curvature of the Vps1 scaffold. Subsequent GTP hydrolysis drives further mechanical constriction of the Vps1 scaffold, forcing the tubule into a super-constricted intermediate state. We observe distinct cycles of thinning and expansion within this intermediate, with the tubule lumen frequently reaching extremely narrow dimensions before stochastically undergoing fission. These continuous oscillations likely capture a steady state governed by concurrent cycles of GTPase-induced constriction and local scaffold disassembly—a mechanical pathway that closely mirrors the fission intermediates observed for mammalian endocytic dynamin (Shnyrova et al, 2013). Furthermore, membrane topology serves as a strict gatekeeper for Vps1 function by restricting its fission activity to tubules with a starting radius below ~30 nm. Based on comparative biophysical data, this rigid 30 nm cutoff positions the mechanical constraints of Vps1 closer to mammalian endocytic dynamin than to mitochondrial Drp1 (Kamerkar et al, 2018; Khurana et al, 2023; Sarkar & Pucadyil, 2025). This restriction arises because Vps1 cannot form organized, constricting scaffolds on tubules wider than this ~30 nm threshold. In a cellular context, Vps1 operates in tandem with membrane-tubulating SNX-BAR proteins on endosomes. Given that native SNX-BAR-coated membrane tubules typically exhibit a radius of ~15 nm (Kovtun et al, 2018), they fall perfectly within the permissive size range required for Vps1 to successfully capitalize on its curvature-sensitive fission activity. Our results further demonstrate that InsB harbors critical, dual determinants for both membrane binding and higher-order self-assembly. Previous bulk vesicle sedimentation assays reported that mutating the 3K motif compromises binding to PI(4,5)P₂ but not to PI(3)P, and concluded that the 3K cluster is dispensable for Vps1 function during endosomal protein sorting (Smaczynska-de Rooij et al, 2019). By leveraging the heightened sensitivity of our PLiMAP assay, our findings challenge this model and indicate otherwise. We show that the 3K motif directly coordinates binding to PI(3)P alongside several other phosphoinositide species. A similar lysine-rich motif is evolutionarily conserved in mammalian mitochondrial Drp1, where it selectively facilitates membrane docking to cardiolipin (Bustillo-Zabalbeitia et al, 2014). Interestingly, we still detect residual membrane binding with the ΔInsB variant, suggesting the existence of auxiliary membrane-binding interfaces situated outside the InsB. In addition to lipid docking, we identified a novel 4 F motif within InsB that actively coordinates membrane-bound self-assembly. Deletion of this motif caused a catastrophic loss of Vps1 fission activity in vitro, a phenotype highly consistent with a fundamental polymerizing defect. Intriguingly, however, the Vps1(Δ4F) variant still organized into smaller focal assemblies within living cells. This apparent discrepancy likely reflects the localized recruitment of the mutant protein via endogenous adapter- or coat-based interactions that are not recapitulated in our purified cell-free assays. Crucially, because the Δ4F, Δ3K, and ΔInsB mutants are uniformly defective in membrane fission and endosomal cargo retrieval, our data underscore that the tight orchestration of both lipid binding and structural self-assembly within InsB is an absolute requirement to license Vps1 functions in vivo.

Quantitative organelle proteomics of isolated vacuoles and lysosomes has previously proven powerful in mapping intracellular trafficking networks (Blondeau et al, 2004; Borner et al, 2006, Borner et al, 2012; Sarry et al, 2007; Eising et al, 2022; Daly et al, 2023). However, to the best of our knowledge, such an approach has not yet been extended to a genetic scenario involving the loss of a primary membrane fission protein. Our proteomic analysis provides novel, system-wide insights into the global alterations that accrue upon the ablation of VPS1. The genetic loss of Vps1 results in the depletion of several soluble luminal enzymes from the yeast vacuole, which in turn triggers a compensatory upregulation of the cytoplasm-to-vacuole targeting (CVT) pathway. This upregulation likely reflects an adaptive mechanism to sustain vacuolar hydrolytic capacity. Classically, the AP-3 pathway delivers the core machinery necessary for vacuolar biogenesis (Eising et al, 2022), and disruption of AP-3 causes the aberrant re-routing of vacuolar membrane proteins to the plasma membrane via the default secretory pathway (Dell’Angelica et al, 1999). A remarkably similar surface re-routing phenotype is observed upon the loss of Vps1 (Nothwehr et al, 1995). Interestingly, the combined deletion of AP-3 and Vps1 is synthetic lethal (Stepp et al, 1997), indicating that the alternative trafficking routes reconfigured in these individual mutant backgrounds must ultimately converge to maintain cell viability. Our findings indicate that a major cohort of trans-Golgi network (TGN) and endosome-localized proteins becomes heavily enriched within the *vps1Δ* vacuolar fraction. The marked increase in their vacuolar abundance in the absence of Vps1 lends robust in vivo credence to current retromer-dependent retrograde trafficking models. The delivery of such a diverse set of non-vacuolar membrane proteins can be parsimoniously explained by the mis-sorting of critical SNARE complexes (such as Pep12), which are themselves established retromer cargoes. In addition to Golgi-resident machinery, VPS1 deletion significantly increases the vacuolar abundance of several nutrient transporters and permeases that are normally targeted for multivesicular body (MVB)-mediated degradation under nutrient-replete conditions. The fact that their levels further escalate in *vps1Δ* vacuoles supports a model wherein these surface transporters continuously cycle out of endosomes via Vps1-dependent retrieval to be recycled back to the plasma membrane. Such continuous, active recycling loop circuits are essential for cells to dynamically monitor the nutritional status of their extracellular environment. While we have rationalized these proteomic alterations using specific candidate cargoes with well-defined trafficking itineraries, our dataset uncovers a much broader repertoire of mis-sorted proteins. This expanded cohort likely contains novel, uncharacterized cargo species that rely on Vps1-dependent pathways. Future systematic cell biology experiments will be required to track the trafficking of these candidates and fully validate the mechanistic models proposed here.

Here, we establish a robust, quantitative imaging assay adaptable to large cell populations to systematically monitor the vacuolar mis-sorting of intracellular cargo. This platform enabled us to rigorously dissect Vps1 functional domains in vivo and cross-validate the trafficking itineraries of diverse retromer-dependent cargoes. Crucially, defects in retromer-mediated retrieval pathways are directly implicated in a wide range of human metabolic and neurodegenerative diseases (Lane et al, 2012; Sebastian et al, 2012; Small and Petsko, 2015; Vagnozzi and Praticò, 2019; Cullen et al, 2024). Our high-throughput yeast-based screening framework offers a powerful, scalable model system to investigate the molecular mechanisms underlying these human pathologies, as well as to identify small-molecule modulators capable of therapeutically restoring retromer pathway function—representing a promising and exciting avenue for future biomedical research.

## Methods

**Table:** Reagents and tools table

Reagent/resource	Reference or source	Identifier or catalog number
**Experimental models**		
*S. cerevisiae*, S288C strain	Saravanan Palani, Indian Institute of Science, Bengaluru	None
**Recombinant DNA**		
pET15B	This study	NA
**Antibodies**		
anti-ALFA-HRP	NanoTag Biotechnologies	N1505-HRP
anti-GAPDH	Abcam	EPR16891
**Oligonucleotides and other sequence-based reagents**		
Primers	Sigma	NA
**Chemicals, enzymes and other reagents**		
All details listed in the materials and methods		
**Software**		
GraphPad Prism	GraphPad	
**Other**		

### DNA constructs

The Vps1 gene was amplified from *S. cerevisiae* genomic DNA and cloned into a pET15b vector with an N-terminal 6xHis tag followed by a TEV cleavage site and a C-terminal StrepII tag. Site-directed mutagenesis was carried out using PCR and confirmed by sequencing. Vps1 and mutants were cloned into pYM16 with an AlFA tag at the C-terminus to generate the VPS1* strains. Datasets EV9 and EV10 list the constructs and strains used in this study, respectively.

### Protein expression and purification

Vps1 was expressed in T7 Express (NEB) using IPTG induction. Transformants were grown to an OD of 0.6–0.8 at 37 °C and induced with 0.1 mM IPTG overnight at 22 °C. Cultures were pelleted and stored at −40 °C. Frozen pellets were thawed in 20 mM HEPES pH 7.4, 500 mM NaCl with 1% Triton X-100 and 1% PMSF and lysed by sonication. Lysates were spun at 30,000×*g* for 20 min, and the supernatant was loaded onto a 5 ml Strep-trap HP or Streptactin XT column (GE Lifesciences). The column was first washed with 20 mM HEPES, pH 7.4, 500 mM NaCl, then with 20 mM HEPES, pH 7.4, 500 mM NaCl containing 100 mM EDTA to remove any bound nucleotides and then with 20 mM HEPES, pH 7.4, and 500 mM NaCl. Buffer was exchanged with 20 mM HEPES, pH 7.4, 150 mM NaCl, and the bound protein was eluted either using 2.5 mM desthiobiotin (Sigma) or 5 mM biotin (HiMedia) in 20 mM HEPES, pH 7.4, 150 mM NaCl. Proteins were spun at 100,000×*g* to remove aggregates before use in assays.

### SEC-MALS analysis

Purified Vps1 (~0.32 mg/ml) was subjected to SEC using a Superose 6 10/300 GL column equilibrated with 20 mM HEPES, pH 7.5, and 150 mM NaCl at room temperature. SEC was coupled with a multi-angle light scattering (MALS) instrument (Wyatt technology) comprising a Wyatt Dawn Helios-II light scattering detector and Wyatt Optilab T-rex refractive index detectors for simultaneous MALS measurements. BSA (2 mg/mL; Pierce) was used as a standard. The data were analyzed using Astra software (Wyatt Technologies).

### Vesicle preparation

Dioleoyl phosphatidylcholine (DOPC), dioleoyl phosphatidylserine (DOPS), and all phosphoinositides were obtained from Avanti Polar Lipids. We aliquoted the lipids in specific proportions into a clean glass tube and removed the solvent under vacuum. After drying, we hydrated the lipids with deionized water to create vesicles and extruded them through a 100-nm pore size filter.

### PLiMAP assays

Proximity-based labeling of membrane-associated proteins (PLiMAP) assays were conducted as described earlier (Jose and Pucadyil, 2020; Jose et al, 2020). The fluorescent crosslinker lipid TMR Diazirine PE (TDPE) was prepared by reacting TopFluor TMR PE (Avanti Polar Lipids) with SDA NHS Diazirine (Thermo Scientific) as previously detailed. We incubated proteins and vesicles at a 1:100 protein-to-lipid molar ratio in 20 mM HEPES (pH 7.4) and 150 mM NaCl for 30 min at room temperature in the dark. For photo-crosslinking, we exposed the mixtures to 365 nm UV light in a UVP CL-1000L crosslinker at an intensity of 200 mJ/cm² for 1 min. Following resolution by SDS-PAGE, we captured TDPE fluorescence using a Typhoon scanner. We then stained the gels with Coomassie Brilliant Blue (CBB) and imaged them on an iBright 1500 (Invitrogen). We processed both images using default particle analysis mask routines, defining membrane binding as the ratio of the fluorescent intensity to the CBB intensity.

### GTPase assays

We assayed GTP hydrolysis by mixing 1 μM of protein with 1 mM GTP (Jena Biosciences) in the presence or absence of 100 μM vesicles. The reactions were carried out at 30 °C in a buffer composed of 20 mM HEPES (pH 7.4), 150 mM NaCl, and 1 mM MgCl₂. At regular intervals, we withdrew 20 μl aliquots and quenched them with 10 μl of 0.5 mM EDTA (pH 8.0). We determined the concentration of released inorganic phosphate using a Malachite Green colorimetric assay (Leonard et al, 2005).

### Fluorescence imaging of supported membrane templates (SMrT) and image analysis

We generated supported membrane templates (SMrT) using previously described protocols (Dar et al, 2017). Lipids and Texas Red DHPE (Thermo Fisher Scientific) were combined in a glass vial to reach a final concentration of 1 mM. We deposited 1–2 μl of the lipid mix onto a covalently PEGylated glass coverslip and allowed it to dry. After assembling the coverslip into an FCS2 flow cell (Bioptechs), we flushed the chamber with a buffer of 20 mM HEPES (pH 7.4) and 150 mM NaCl. This flow generated a supported bilayer at the original spot and produced membrane tubes downstream that settled onto the passivated glass surface. We then introduced 0.5 μM of protein prepared in the same buffer supplemented with 1 mM nucleotides (Jena Biosciences) and 1 mM MgCl₂. All time-lapse imaging was conducted in a buffer containing an oxygen scavenger cocktail (Roy and Pucadyil, 2022; Dar et al, 2017). For fluorescence visualization of Vps1 on the membrane tubes, we incubated 0.5 μM Vps1 with a tenfold excess of Alexa Fluor 488-C2 maleimide (Thermo Scientific) for 5 min before introducing it to the templates. We incubated the system for 10 min, washed out excess unbound protein and dye, and then captured the images. We performed all imaging using a motorized Olympus IX73 microscope equipped with a 100x/1.4 NA oil-immersion objective, a pE4000 (CoolLED) illumination system, and an Evolve EMCCD camera (Photometrics) operated by µManager software. We calculated the membrane tube radii by utilizing the supported bilayer as an internal calibration standard. Briefly, we subtracted the background and measured the integrated fluorescence per unit area of the supported bilayer. We calculated the area of 1-μm tube segments from their integrated fluorescence and derived the radius assuming a cylindrical shape. Linear regression of the peak tube fluorescence against these calculated radii yielded a calibration slope. We divided all pixel values in the tube images by this calibration constant to generate radius maps. We analyzed and rendered images using Fiji and conducted all data plotting and regression analysis in GraphPad Prism (version 10.0).

### Yeast strain generation

Gene deletion, replacement and tagging with fluorescent proteins were carried out according to Janke et al, (Janke et al, 2004). Primers were of HPLC-purified grade (Sigma). PCR amplicons generated using primers that are complementary to 50 bp upstream and downstream of the VPS1 locus were used to transform *S. cerevisiae* (strain S288C) cells according to Gietz et al, (Gietz et al, 1995). The VPS1* strain with mutants was generated in the *vps1Δ* background. Yeast strains used here are listed in Dataset EV10.

### Fluorescence imaging of yeast cells and image analysis

Primary cultures were grown overnight to saturation in appropriate media and diluted to an initial optical density of 0.1–0.2 to start secondary cultures. Upon reaching an optical density of 0.6–0.8, 1.5 ml of the culture was centrifuged and resuspended in 0.2 ml of synthetic complete medium. Eight-well Lab-Tek chamber slides were etched with 5 N sodium hydroxide for 30 min, washed with deionized water, and coated with 0.5 mg/ml of Concanavalin A (Sigma) in DPBS for 30 min. After washing the chambers with DPBS, 0.2 ml of the yeast cell suspension was incubated on the coated surface for 30 min, washed three to five times, and maintained in synthetic complete medium for imaging. Live-cell imaging was conducted on an Evident SpinSR spinning-disk confocal microscope using a 100X, 1.45 NA oil-immersion objective. To quantify vacuolar localization, 1024 × 1024 pixel single-plane images were acquired from several fields of cells expressing cargo-mNG and Vph1-mSc across three independent experiments. Image analysis was performed by stacking the channels separately and generating a binary mask from the Vph1-mSc channel using default threshold settings. This mask was applied to the cargo-mNG images to measure the fluorescence intensity of particles with an area ≥1.69 μm^2^ and circularity between 0 and 1. To correct for background autofluorescence in the mNG channel, a strain expressing Vph1-mSc alone was imaged under identical conditions, and the median background signal was subtracted from the cargo-mNG intensities. The resulting data were plotted as a frequency distribution and fitted to a Gaussian curve to determine the median, with all values normalized to the median obtained for vps1Δ cells. Photobleaching in live-cell movies was corrected using the histogram matching option in Fiji.

### Spot growth assay

Cells were cultured to an OD of 0.6–0.7. They were pelleted and resuspended in deionized water. Five sequential tenfold serial dilutions were prepared in a 96-well plate. Aliquots of 5 μl were spotted onto YPD agar plates. The plates were incubated at 30 and 37 °C for 2 days and then imaged.

### Western blotting

Cells were grown to an OD of 0.6–0.8 and pelleted at 3200×*g* for 5 min at 4 °C. Cell pellets were resuspended in 0.8 ml of ice-cold, autoclaved deionized water and immediately lysed by the addition of 0.15 ml of 1.85 N NaOH. Following vortexing, the mixtures were incubated on ice for 5 min. Proteins were then precipitated by adding 0.15 ml of 55% (w/w) trichloroacetic acid (TCA), followed by vortexing and a 10-min incubation on ice. The precipitated proteins were collected by centrifugation at 18,000×*g* for 20 min at 4 °C. The supernatant was discarded, and a subsequent 5-min spin at 18,000×*g* was performed to remove any residual acid. The resulting pellets were resuspended in HU-DTT buffer (8 M urea, 5% SDS, 200 mM Tris-HCl at pH 6.8, 0.1 mM EDTA, bromophenol blue, and 10 mM DTT) and heated at 99 °C for 20 min. Equal volumes of the prepared samples were resolved on a 10% SDS-PAGE gel and transferred onto a PVDF membrane. The membranes were blocked using 5% skimmed milk in 1X TBST. For immunodetection, the blot was cut and incubated with an anti-ALFA-HRP antibody (NanoTag Biotechnologies, catalog number N1505-HRP) or an anti-GAPDH antibody (Abcam, catalog number EPR16891) for 3 h in 5% skimmed milk, washed three times with 1X TBST, and analyzed for chemiluminescence on an iBright 1500 system (Invitrogen). All images were processed and rendered using Fiji.

### Sequence alignment

The *S. cerevisiae* InsB sequence was checked for homology with other fungal sequences using the Fungal Blast algorithm on the Saccharomyces Genome Database (Engel et al, 2025). Sequences showing significant similarities were collated and run through Clustal Omega (Madeira et al, 2024). Results in FASTA format converted by Seqret were rendered using SnapGene.

### Vacuole isolation

Vacuoles were isolated according to previously described protocols (Cabrera and Ungermann, 2008). Cells were cultured to an OD of 0.6–0.8 and harvested by centrifugation at 3200×*g* for 5 min. The resulting pellets were resuspended in a DTT solution (0.1 mM Tris, pH 9.4, 10 mM DTT) and incubated in a water bath at 30 °C for 20 min. The cells were then pelleted again at 3200×*g* for 5 min. Following supernatant removal, the cells were resuspended in spheroplasting buffer (0.1X YPD, 0.6 M sorbitol, and 50 mM potassium phosphate at pH 7.5) containing 1 mg/mL of lyticase (Sigma, L4025-25KU) and incubated at 30 °C for 30 min. Spheroplasts were collected at 5300×*g* for 5 min and resuspended in 15% (w/v) Ficoll prepared in PS buffer (10 mM Pipes/KOH, pH 6.8, and 200 mM sorbitol). This suspension was mixed with 0.4 mg/mL DEAE dextran (Sigma, 93556) and transferred to a Beckman Coulter SW40 tube. The mixture was kept on ice for 5 min and then shifted to 30 °C for 1.5 min. A step gradient of 8, 4, and 0% (w/v) Ficoll in PS buffer was layered on top. The tubes were centrifuged at 120,000×*g* for 90 min at 4 °C in an SW40 rotor. The vacuole fraction at the 0% and 4% Ficoll interface was carefully collected. Finally, the fraction was solubilized using 1% Triton X-100, and the total protein concentration was measured by BCA assay using BSA as a standard.

### Proteomic analysis

Proteomic analysis was carried out by Valerian Chem Pvt. Ltd. The sample (100 μg of total protein) was first reduced with 5 mM TCEP and subsequently alkylated with 50 mM iodoacetamide. Samples were digested with trypsin at a 1:50 (trypsin:protein ratio) for 16 h at 37 °C. Peptides were purified using a C18 silica cartridge and then concentrated by drying in a SpeedVac. The dried pellet was resuspended in buffer A (2% acetonitrile and 0.1% formic acid). Mass spectrometric analysis of peptides was performed on an Easy-nlc-1000 system coupled with an Orbitrap Exploris 480 mass spectrometer. About 1 μg of peptide sample was loaded on a PicoFrit column (1.9-micron resin, 15 cm length) and separated with a 0–38% gradient of buffer B (80% acetonitrile and 0.1% formic acid) at a flow rate of 500 nl/min and injected for MS analysis. The runtime for LC-MS acquisition was 60 min. MS1 spectra were acquired in the Orbitrap at a resolution of 60,000 with Max IT = 60 ms, AGC target = 300%; RF lens = 50% and mass range = 375−1500. Dynamic exclusion was employed for 30 s, excluding all charge states for a given precursor. MS2 spectra were collected for the top 13 peptides. MS/MS data were acquired at a resolution of 15,000 with Max IT = 60 ms, AGC target =  100%. Raw files containing m/z values were generated for each sample. Raw files were analyzed on DIA-NN 2.3.1 Academia against the *S. cerevisiae* Uniprot reference database. The protease used to generate peptides was set to trypsin/P (cleavage at the C terminus of K/R unless followed by P), along with a maximum missed cleavages value of two. Carbamidomethylation on C residues as a fixed modification, methionine oxidation, and N-terminal acetylation were considered variable modifications for the database search. Both peptide spectrum match and protein false discovery rate were set to 0.01. Significance levels were calculated using an unpaired, two-tailed Student’s *t*-test, assuming a parametric distribution of the data.

### Data analysis

Data were plotted and analyzed using GraphPad Prism (version 10.0).

## Supplementary information


Peer Review File
Dataset EV1
Dataset EV2
Dataset EV3
Dataset EV4
Dataset EV5
Dataset EV6
Dataset EV7
Dataset EV8
Dataset EV9
Dataset EV10
Movie EV1
Movie EV2
Movie EV3
Movie EV4
Movie EV5
Movie EV6
Movie EV7
Movie EV8
Movie EV9
Movie EV10
Movie EV11
Movie EV12
Movie EV13
Movie EV14
Movie EV15
Movie EV16
Movie EV17
Movie EV18
Movie EV19
Movie EV20
Movie EV21
Movie EV22
Source data Fig. 1
Source data Fig. 2
Source data Fig. 3
Source data Fig. 4
Source data Fig. 5
Source data Fig. 6
Figure EV1 Source Data
Figure EV2 Source Data
Expanded View Figures


## Data Availability

The mass spectrometry data from this publication have been deposited in the ProteomeXchange Consortium via the PRIDE partner repository database [https://www.ebi.ac.uk/pride/] (Perez-Riverol et al, 2025) and assigned the identifier [PXD077915] with the access token 0njVcOCxx833. The source data of this paper are collected in the following database record: biostudies:S-SCDT-10_1038-S44318-026-00855-4.

## References

[CR1] Akhuli D, Dhar A, Viji AS, Bhojappa B, Palani S (2022) ALIBY: ALFA nanobody-based toolkit for imaging and biochemistry in yeast. mSphere 7:e00333–22 36190134 10.1128/msphere.00333-22 PMC9599267

[CR2] Antonny B, Burd C, De Camilli P, Chen E, Daumke O, Faelber K, Ford M, Frolov VA, Frost A, Hinshaw JE et al (2016) Membrane fission by dynamin: what we know and what we need to know. EMBO J 35:2270–2284 27670760 10.15252/embj.201694613 PMC5090216

[CR3] Arlt H, Raman B, Filali-Mouncef Y, Hu Y, Leytens A, Hardenberg R, Guimarães R, Kriegenburg F, Mari M, Smaczynska-de Rooij II et al (2023) The dynamin Vps1 mediates Atg9 transport to the sites of autophagosome formation. J Biol Chem 299:104712 37060997 10.1016/j.jbc.2023.104712 PMC10196871

[CR4] Arlt H, Reggiori F, Ungermann C (2014) Retromer and the dynamin Vps1 cooperate in the retrieval of transmembrane proteins from vacuoles. J Cell Sci 128:645–655 10.1242/jcs.132720 25512334

[CR5] Bai L, You Q, Jain BK, Duan HD, Kovach A, Graham TR, Li H (2020) Transport mechanism of P4 ATPase phosphatidylcholine flippases. eLife 9:e62163 33320091 10.7554/eLife.62163 PMC7773333

[CR6] Bankaitis VA, Johnson LM, Emr SD (1986) Isolation of yeast mutants defective in protein targeting to the vacuole. Proc Natl Acad Sci USA 83:9075–9079 3538017 10.1073/pnas.83.23.9075 PMC387077

[CR7] Baratam K, Jha K, Srivastava A (2021) Flexible pivoting of dynamin pleckstrin homology domain catalyzes fission: insights into molecular degrees of freedom. Mol Biol Cell 32:1306–1319 33979205 10.1091/mbc.E20-12-0794 PMC8351549

[CR8] Blondeau F, Ritter B, Allaire PD, Wasiak S, Girard M, Hussain NK, Angers A, Legendre-Guillemin V, Roy L, Boismenu D et al (2004) Tandem MS analysis of brain clathrin-coated vesicles reveals their critical involvement in synaptic vesicle recycling. Proc Natl Acad Sci USA 101:3833–3838 15007177 10.1073/pnas.0308186101 PMC374330

[CR9] Bonifacino JS, Hurley JH (2008) Retromer. Curr Opin Cell Biol 20:427–436 18472259 10.1016/j.ceb.2008.03.009 PMC2833274

[CR10] Borner GHH, Antrobus R, Hirst J, Bhumbra GS, Kozik P, Jackson LP, Sahlender DA, Robinson MS (2012) Multivariate proteomic profiling identifies novel accessory proteins of coated vesicles. J Cell Biol 197:141–160 22472443 10.1083/jcb.201111049 PMC3317806

[CR11] Borner GHH, Harbour M, Hester S, Lilley KS, Robinson MS (2006) Comparative proteomics of clathrin-coated vesicles. J Cell Biol 175:571–578 17116749 10.1083/jcb.200607164 PMC2064594

[CR12] Burd C, Cullen PJ (2014) Retromer: a master conductor of endosome sorting. Cold Spring Harb Perspect Biol 6:a016774–a016774 24492709 10.1101/cshperspect.a016774 PMC3941235

[CR13] Burda P, Padilla SM, Sarkar S, Emr SD (2002) Retromer function in endosome-to-Golgi retrograde transport is regulated by the yeast Vps34 PtdIns 3-kinase. J Cell Sci 115:3889–3900 12244127 10.1242/jcs.00090

[CR14] Burri L, Lithgow T (2004) A complete set of SNAREs in yeast. Traffic 5:45–52 14675424 10.1046/j.1600-0854.2003.00151.x

[CR15] Bustillo-Zabalbeitia I, Montessuit S, Raemy E, Basañez G, Terrones O, Martinou J-C (2014) Specific interaction with cardiolipin triggers functional activation of dynamin-related protein 1. PLoS ONE 9:e102738 25036098 10.1371/journal.pone.0102738 PMC4103857

[CR16] Cabrera M, Ungermann C (2008) Purification and in vitro analysis of yeast vacuoles. Methods Enzymol 451:177–196 10.1016/S0076-6879(08)03213-8 19185721

[CR17] Chi RJ, Liu J, West M, Wang J, Odorizzi G, Burd CG (2014) Fission of SNX-BAR–coated endosomal retrograde transport carriers is promoted by the dynamin-related protein Vps1. J Cell Biol 204:793–806 24567361 10.1083/jcb.201309084 PMC3941054

[CR18] Clinton RW, Francy CA, Ramachandran R, Qi X, Mears JA (2016) Dynamin-related protein 1 oligomerization in solution impairs functional interactions with membrane-anchored mitochondrial fission factor. J Biol Chem 291:478–492 26578514 10.1074/jbc.M115.680025 PMC4697186

[CR19] Cooper AA, Stevens TH (1996) Vps10p cycles between the late-Golgi and prevacuolar compartments in its function as the sorting receptor for multiple yeast vacuolar hydrolases. J Cell Biol 133:529–541 8636229 10.1083/jcb.133.3.529 PMC2120820

[CR20] Cullen PJ (2008) Endosomal sorting and signalling: an emerging role for sorting nexins. Nat Rev Mol Cell Biol 9:574–582 18523436 10.1038/nrm2427

[CR21] Cullen PJ, Holstege H, Small SA, St George-Hyslop P (2024) Understanding the endo-lysosomal network in neurodegeneration. Philos Trans R Soc B Biol Sci 379:20220372 10.1098/rstb.2022.0372 PMC10874696 38368931

[CR22] Dalton LE, Bean BDM, Davey M, Conibear E (2017) Quantitative high-content imaging identifies novel regulators of Neo1 trafficking at endosomes. Mol Biol Cell 28:1539–1550 28404745 10.1091/mbc.E16-11-0772 PMC5449152

[CR23] Daly JL, Danson CM, Lewis PA, Zhao L, Riccardo S, Di Filippo L, Cacchiarelli D, Lee D, Cross SJ, Heesom KJ et al (2023) Multi-omic approach characterises the neuroprotective role of retromer in regulating lysosomal health. Nat Commun 14:3086 37248224 10.1038/s41467-023-38719-8 PMC10227043

[CR24] Dar S, Kamerkar SC, Pucadyil TJ (2017) Use of the supported membrane tube assay system for real-time analysis of membrane fission reactions. Nat Protoc 12:390–400 28125102 10.1038/nprot.2016.173

[CR25] Dar S, Pucadyil TJ (2017) The pleckstrin-homology domain of dynamin is dispensable for membrane constriction and fission. Mol Biol Cell 28:152–160 28035046 10.1091/mbc.E16-09-0640 PMC5221619

[CR26] Darsow T, Rieder SE, Emr SD (1997) A multispecificity syntaxin homologue, Vam3p, essential for autophagic and biosynthetic protein transport to the vacuole. J Cell Biol 138:517–529 9245783 10.1083/jcb.138.3.517 PMC2141632

[CR27] Day KJ, Casler JC, Glick BS (2018) Budding yeast has a minimal endomembrane system. Dev Cell 44:56–72.e4 29316441 10.1016/j.devcel.2017.12.014 PMC5765772

[CR28] Dell’Angelica EC, Shotelersuk V, Aguilar RC, Gahl WA, Bonifacino JS (1999) Altered trafficking of lysosomal proteins in Hermansky-Pudlak syndrome due to mutations in the β3A subunit of the AP-3 adaptor. Mol Cell 3:11–21 10024875 10.1016/s1097-2765(00)80170-7

[CR29] Eising S, Esch B, Wälte M, Vargas Duarte P, Walter S, Ungermann C, Bohnert M, Fröhlich F (2022) A lysosomal biogenesis map reveals the cargo spectrum of yeast vacuolar protein targeting pathways. J Cell Biol 221:e202107148 35175277 10.1083/jcb.202107148 PMC8859911

[CR30] Ekal L, Alqahtani AMS, Hettema EH (2023) The dynamin-related protein Vps1 and the peroxisomal membrane protein Pex27 function together during peroxisome fission. J Cell Sci 136:jcs246348 36825558 10.1242/jcs.246348 PMC10112978

[CR31] Engel SR, Aleksander S, Nash RS, Wong ED, Weng S, Miyasato SR, Sherlock G, Cherry JM (2025) *Saccharomyces* Genome Database: advances in genome annotation, expanded biochemical pathways, and other key enhancements. Genetics 229:iyae185 39530598 10.1093/genetics/iyae185 PMC11912841

[CR32] Ford MGJ, Chappie JS (2019) The structural biology of the dynamin-related proteins: new insights into a diverse, multitalented family. Traffic 20:717–740 31298797 10.1111/tra.12676 PMC6876869

[CR33] Gietz RD, Schiestl RH, Willems AR, Woods RA (1995) Studies on the transformation of intact yeast cells by the LiAc/SS-DNA/PEG procedure. Yeast 11:355–360 7785336 10.1002/yea.320110408

[CR34] Götzke H, Kilisch M, Martínez-Carranza M, Sograte-Idrissi S, Rajavel A, Schlichthaerle T, Engels N, Jungmann R, Stenmark P, Opazo F et al (2019) The ALFA-tag is a highly versatile tool for nanobody-based bioscience applications. Nat Commun 10:4403 31562305 10.1038/s41467-019-12301-7 PMC6764986

[CR35] Hayden J, Williams M, Granich A, Ahn H, Tenay B, Lukehart J, Highfill C, Dobard S, Kim K (2013) Vps1 in the late endosome-to-vacuole traffic. J Biosci 38:73–83 23385815 10.1007/s12038-012-9295-2

[CR36] Hettema EH, Lewis MJ, Black MW, Pelham HRB (2003) Retromer and the sorting nexins Snx4/41/42 mediate distinct retrieval pathways from yeast endosomes. EMBO J 22:548–557 12554655 10.1093/emboj/cdg062 PMC140746

[CR37] Horazdovsky BF, Davies BA, Seaman MN, McLaughlin SA, Yoon S, Emr SD (1997) A sorting nexin-1 homologue, Vps5p, forms a complex with Vps17p and is required for recycling the vacuolar protein-sorting receptor. Mol Biol Cell 8:1529–1541 9285823 10.1091/mbc.8.8.1529 PMC276174

[CR38] Hu Y, Reggiori F (2023) The yeast dynamin-like GTPase Vps1 mediates Atg9 transport to the phagophore assembly site in *Saccharomyces cerevisiae*. Autophagy Rep 2:2247309 38107506 10.1080/27694127.2023.2247309 PMC7615383

[CR39] Janke C, Magiera MM, Rathfelder N, Taxis C, Reber S, Maekawa H, Moreno-Borchart A, Doenges G, Schwob E, Schiebel E et al (2004) A versatile toolbox for PCR-based tagging of yeast genes: new fluorescent proteins, more markers and promoter substitution cassettes. Yeast 21:947–962 15334558 10.1002/yea.1142

[CR40] Jimah JR, Hinshaw JE (2019) Structural insights into the mechanism of dynamin superfamily proteins. Trends Cell Biol 29:257–273 30527453 10.1016/j.tcb.2018.11.003 PMC9623552

[CR41] Jiménez M, Kyoung CK, Nabukhotna K, Watkins D, Jain BK, Best JT, Graham TR (2024) P4-ATPase endosomal recycling relies on multiple retromer-dependent localization signals. Mol Biol Cell 35:ar125 39110530 10.1091/mbc.E24-05-0209 PMC11481694

[CR42] Jose GP, Gopan S, Bhattacharyya S, Pucadyil TJ (2020) A facile, sensitive and quantitative membrane-binding assay for proteins. Traffic 21:297–305 31846132 10.1111/tra.12719

[CR43] Jose GP, Pucadyil TJ (2020) PLiMAP: proximity-based labeling of membrane-associated proteins. Curr Protoc Protein Sci 101:e110 32603530 10.1002/cpps.110

[CR44] Kamerkar SC, Kraus F, Sharpe AJ, Pucadyil TJ, Ryan MT (2018) Dynamin-related protein 1 has membrane constricting and severing abilities sufficient for mitochondrial and peroxisomal fission. Nat Commun 9:5239 30531964 10.1038/s41467-018-07543-w PMC6286342

[CR45] Khurana H, Baratam K, Bhattacharyya S, Srivastava A, Pucadyil TJ (2023) Mechanistic analysis of a novel membrane-interacting variable loop in the pleckstrin-homology domain critical for dynamin function. Proc Natl Acad Sci USA 120:e2215250120 36888655 10.1073/pnas.2215250120 PMC10089193

[CR46] Khurana H, Pucadyil TJ (2023) “Gearing” up for dynamin-catalyzed membrane fission. Curr Opin Cell Biol 83:102204 37451176 10.1016/j.ceb.2023.102204

[CR47] Kovtun O, Leneva N, Bykov YS, Ariotti N, Teasdale RD, Schaffer M, Engel BD, Owen DJ, Briggs JAG, Collins BM (2018) Structure of the membrane-assembled retromer coat determined by cryo-electron tomography. Nature 561:561–564 30224749 10.1038/s41586-018-0526-z PMC6173284

[CR48] Kuravi K, Nagotu S, Krikken AM, Sjollema K, Deckers M, Erdmann R, Veenhuis M, Van Der Klei IJ (2006) Dynamin-related proteins Vps1p and Dnm1p control peroxisome abundance in *Saccharomyces cerevisiae*. J Cell Sci 119:3994–4001 16968746 10.1242/jcs.03166

[CR49] Lane RF, St George-Hyslop P, Hempstead BL, Small SA, Strittmatter SM, Gandy S (2012) Vps10 family proteins and the retromer complex in aging-related neurodegeneration and diabetes. J Neurosci 32:14080–14086 23055476 10.1523/JNEUROSCI.3359-12.2012 PMC3576841

[CR50] Leonard M, Doo Song B, Ramachandran R, Schmid SL (2005) Robust colorimetric assays for dynamin’s basal and stimulated GTPase activities. Methods Enzymol 404:490–503 10.1016/S0076-6879(05)04043-7 16413294

[CR51] Lewis MJ, Pelham HRB (2002) A new yeast endosomal SNARE related to mammalian syntaxin 8. Traffic 3:922–929 12453154 10.1034/j.1600-0854.2002.31207.x

[CR52] Ma M, Burd CG, Chi RJ (2017a) Distinct complexes of yeast Snx4 family SNX-BARs mediate retrograde trafficking of Snc1 and Atg27. Traffic 18:134–144 28026081 10.1111/tra.12462 PMC5262529

[CR53] Ma M, Burd CG, RJ Chi (2017b) Distinct complexes of yeast Snx4 family SNX-BARs mediate retrograde trafficking of Snc1 and Atg27. Traffic 18:134–144 28026081 10.1111/tra.12462 PMC5262529

[CR54] Madeira F, Madhusoodanan N, Lee J, Eusebi A, Niewielska A, Tivey ARN, Lopez R, Butcher S (2024) The EMBL-EBI Job Dispatcher sequence analysis tools framework in 2024. Nucleic Acids Res 52:W521–W525 38597606 10.1093/nar/gkae241 PMC11223882

[CR55] Marat AL, Haucke V (2016) Phosphatidylinositol 3-phosphates—at the interface between cell signalling and membrane traffic. EMBO J 35:561–579 26888746 10.15252/embj.201593564 PMC4801949

[CR56] Marcusson EG, Horazdovsky BF, Cereghino JL, Gharakhanian E, Emr SD (1994) The sorting receptor for yeast vacuolar carboxypeptidase Y is encoded by the VPS10 gene. Cell 77:579–586 8187177 10.1016/0092-8674(94)90219-4

[CR57] McGough IJ, De Groot REA, Jellett AP, Betist MC, Varandas KC, Danson CM, Heesom KJ, Korswagen HC, Cullen PJ (2018) SNX3-retromer requires an evolutionary conserved MON2:DOPEY2:ATP9A complex to mediate Wntless sorting and Wnt secretion. Nat Commun 9:3737 30213940 10.1038/s41467-018-06114-3 PMC6137200

[CR58] Nannapaneni S, Wang D, Jain S, Schroeder B, Highfill C, Reustle L, Pittsley D, Maysent A, Moulder S, McDowell R et al (2010) The yeast dynamin-like protein Vps1:vps1 mutations perturb the internalization and the motility of endocytic vesicles and endosomes via disorganization of the actin cytoskeleton. Eur J Cell Biol 89:499–508 20189679 10.1016/j.ejcb.2010.02.002

[CR59] Nothwehr SF, Conibear E, Stevens TH (1995) Golgi and vacuolar membrane proteins reach the vacuole in vps1 mutant yeast cells via the plasma membrane. J Cell Biol 129:35–46 7698993 10.1083/jcb.129.1.35 PMC2120360

[CR60] Palmer SE, Smaczynska-de Rooij II, Marklew CJ, Allwood EG, Mishra R, Johnson S, Goldberg MW, Ayscough KR (2015) A Dynamin-actin interaction is required for vesicle scission during endocytosis in yeast. Curr Biol 25:868–878 25772449 10.1016/j.cub.2015.01.061 PMC4386032

[CR61] Pemberton JG, Roy K, Kim YJ, Fischer TD, Joshi V, Ferrer E, Youle RJ, Pucadyil TJ, Balla T (2025) Acute diacylglycerol production activates critical membrane-shaping proteins leading to mitochondrial tubulation and fission. Nat Commun 16:2685 40102394 10.1038/s41467-025-57439-9 PMC11920102

[CR62] Perez-Riverol Y, Bandla C, Kundu DJ, Kamatchinathan S, Bai J, Hewapathirana S, John NS, Prakash A, Walzer M, Wang S et al (2025) The PRIDE database at 20 years: 2025 update. Nucleic Acids Res 53:D543–D553 39494541 10.1093/nar/gkae1011 PMC11701690

[CR63] Peters C, Baars TL, Bühler S, Mayer A (2004) Mutual control of membrane fission and fusion proteins. Cell 119:667–678 15550248 10.1016/j.cell.2004.11.023

[CR64] Posor Y, Jang W, Haucke V (2022) Phosphoinositides as membrane organizers. Nat Rev Mol Cell Biol 23:797–816 35589852 10.1038/s41580-022-00490-x PMC9117997

[CR65] Purushothaman LK, Ungermann C (2018) Cargo induces retromer-mediated membrane remodeling on membranes. Mol Biol Cell 29:2709–2719 30188774 10.1091/mbc.E18-06-0339 PMC6249844

[CR66] Raymond CK, Howald-Stevenson I, Vater CA, Stevens TH (1992) Morphological classification of the yeast vacuolar protein sorting mutants: evidence for a prevacuolar compartment in class E vps mutants. Mol Biol Cell 3:1389–1402 1493335 10.1091/mbc.3.12.1389 PMC275707

[CR67] Robinson JS, Klionsky DJ, Banta LM, Emr SD (1988) Protein sorting in *Saccharomyces cerevisiae*: isolation of mutants defective in the delivery and processing of multiple vacuolar hydrolases. Mol Cell Biol 8:4936–4948 3062374 10.1128/mcb.8.11.4936-4948.1988 PMC365587

[CR68] Rooij IIS, Allwood EG, Aghamohammadzadeh S, Hettema EH, Goldberg MW, Ayscough KR (2010) A role for the dynamin-like protein Vps1 during endocytosis in yeast. J Cell Sci 123:3496–3506 20841380 10.1242/jcs.070508 PMC2951468

[CR69] Röthlisberger S, Jourdain I, Johnson C, Takegawa K, Hyams JS (2009) The dynamin-related protein Vps1 regulates vacuole fission, fusion and tubulation in the fission yeast, *Schizosaccharomyces pombe*. Fungal Genet Biol 46:927–935 19643199 10.1016/j.fgb.2009.07.008

[CR70] Rothman JH, Raymond CK, Gilbert T, O’Hara PJ, Stevens TH (1990) A putative GTP binding protein homologous to interferon-inducible Mx proteins performs an essential function in yeast protein sorting. Cell 61:1063–1074 2112425 10.1016/0092-8674(90)90070-u

[CR71] Rothman JH, Stevens TH (1986) Protein sorting in yeast: mutants defective in vacuole biogenesis mislocalize vacuolar proteins into the late secretory pathway. Cell 47:1041–1051 3536126 10.1016/0092-8674(86)90819-6

[CR72] Roy K, Pucadyil TJ (2022) Is Drp1 sufficient to catalyze membrane fission? Proc Natl Acad Sci USA 119:e2201709119 35727965 10.1073/pnas.2201709119 PMC9271209

[CR73] Sarkar M & Pucadyil TJ (2025) Division of labor among fission dynamins based on substrate size. Biochemistry 64:2117–2122 10.1021/acs.biochem.4c00862 40305847

[CR74] Sarry J, Chen S, Collum RP, Liang S, Peng M, Lang A, Naumann B, Dzierszinski F, Yuan C, Hippler M et al (2007) Analysis of the vacuolar luminal proteome of *Saccharomyces cerevisiae*. FEBS J 274:4287–4305 17651441 10.1111/j.1742-4658.2007.05959.x

[CR75] Seaman MN, McCaffery JM, Emr SD (1998) A membrane coat complex essential for endosome-to-Golgi retrograde transport in yeast. J Cell Biol 142:665–681 9700157 10.1083/jcb.142.3.665 PMC2148169

[CR76] Sebastian TT, Baldridge RD, Xu P, Graham TR (2012) Phospholipid flippases: building asymmetric membranes and transport vesicles. Biochim Biophys Acta 1821:1068–1077 22234261 10.1016/j.bbalip.2011.12.007 PMC3368091

[CR77] Shnyrova AV, Bashkirov PV, Akimov SA, Pucadyil TJ, Zimmerberg J, Schmid SL, Frolov VA (2013) Geometric catalysis of membrane fission driven by flexible dynamin rings. Science 339:1433–1436 23520112 10.1126/science.1233920 PMC3980720

[CR78] Shortill SP, Frier MS, Conibear E (2022a) You can go your own way: SNX-BAR coat complexes direct traffic at late endosomes. Curr Opin Cell Biol 76:102087 35569261 10.1016/j.ceb.2022.102087

[CR79] Shortill SP, Frier MS, Wongsangaroonsri P, Davey M, Conibear E (2022b) The VINE complex is an endosomal VPS9-domain GEF and SNX-BAR coat. eLife 11:e77035 35938928 10.7554/eLife.77035 PMC9507130

[CR80] Smaczynska-de Rooij II, Marklew CJ, Allwood EG, Palmer SE, Booth WI, Mishra R, Goldberg MW, Ayscough KR (2016) Phosphorylation regulates the endocytic function of the yeast dynamin-related protein Vps1. Mol Cell Biol 36:742–755 10.1128/MCB.00833-15 PMC4760221 26711254

[CR81] Smaczynska-de Rooij II, Marklew CJ, Palmer SE, Allwood EG, Ayscough KR (2019) Mutation of key lysine residues in the Insert B region of the yeast dynamin Vps1 disrupts lipid binding and causes defects in endocytosis. PLoS ONE 14:e0215102 31009484 10.1371/journal.pone.0215102 PMC6476499

[CR82] Small SA, Petsko GA (2015) Retromer in Alzheimer disease, Parkinson disease and other neurological disorders. Nat Rev Neurosci 16:126–132 25669742 10.1038/nrn3896

[CR83] Stepp JD, Huang K, Lemmon SK (1997) The yeast adaptor protein complex, AP-3, is essential for the efficient delivery of alkaline phosphatase by the alternate pathway to the vacuole. J Cell Biol 139:1761–1774 9412470 10.1083/jcb.139.7.1761 PMC2132655

[CR84] Strochlic TI, Schmiedekamp BC, Lee J, Katzmann DJ, Burd CG (2008) Opposing activities of the Snx3-retromer complex and ESCRT proteins mediate regulated cargo sorting at a common endosome. Mol Biol Cell 19:4694–4706 18768754 10.1091/mbc.E08-03-0296 PMC2575174

[CR85] Suzuki SW, Oishi A, Nikulin N, Jorgensen JR, Baile MG, Emr SD (2021) A PX-BAR protein Mvp1/SNX8 and a dynamin-like GTPase Vps1 drive endosomal recycling. eLife 10:e69883 34524084 10.7554/eLife.69883 PMC8504969

[CR86] Swaminathan U, Pucadyil TJ (2024) Reconstituting membrane fission using a high content and throughput assay. Biochem Soc Trans 52:1449–1457 38747723 10.1042/BST20231325

[CR87] Vagnozzi AN, Praticò D (2019) Endosomal sorting and trafficking, the retromer complex and neurodegeneration. Mol Psychiatry 24:857–868 30120416 10.1038/s41380-018-0221-3 PMC6378136

[CR88] Varlakhanova NV, Alvarez FJD, Brady TM, Tornabene BA, Hosford CJ, Chappie JS, Zhang P, Ford MGJ (2018) Structures of the fungal dynamin-related protein Vps1 reveal a unique, open helical architecture. J Cell Biol 217:3608–3624 30087125 10.1083/jcb.201712021 PMC6168280

[CR89] Vizeacoumar FJ, Vreden WN, Fagarasanu M, Eitzen GA, Aitchison JD, Rachubinski RA (2006) The Dynamin-like protein Vps1p of the yeast *Saccharomyces cerevisiae* associates with peroxisomes in a Pex19p-dependent manner. J Biol Chem 281:12817–12823 16520372 10.1074/jbc.M600365200

[CR90] Voos W, Stevens TH (1998) Retrieval of resident late-Golgi membrane proteins from the prevacuolar compartment of *Saccharomyces cerevisiae* is dependent on the function of Grd19p. J Cell Biol 140:577–590 9456318 10.1083/jcb.140.3.577 PMC2140161

[CR91] Wendland B, Emr SD, Riezman H (1998) Protein traffic in the yeast endocytic and vacuolar protein sorting pathways. Curr Opin Cell Biol 10:513–522 9719873 10.1016/s0955-0674(98)80067-7

[CR92] Yeung T, Gilbert GE, Shi J, Silvius J, Kapus A, Grinstein S (2008) Membrane phosphatidylserine regulates surface charge and protein localization. Science 319:210–213 18187657 10.1126/science.1152066

