# Supplementary material for: Vps1 drives membrane constriction and fission necessary for endosomal protein sorting
Source: EMBO J. 2026 Jul 20;45(17):6115–38. doi: 10.1038/s44318-026-00855-4 (PMC13534707; doi:10.1038/s44318-026-00855-4)
Supplement: Supplementary file 1 [file 44318_2026_855_MOESM1_ESM.pdf]

THE EMBO JOURNAL

Vps1 drives membrane constriction and fission necessary for endosomal protein sorting

Shilpa Gopan, Uma Swaminathan, Gurmail Singh, and Thomas Pucadyil

Corresponding author(s): Thomas Pucadyil (pucadyil@iiserpune.ac.in)

Review Timeline:

Submission Date:	29th Jul 25
Editorial Decision:	1st Aug 25
Revision Received:	3rd May 26
Editorial Decision:	16th Jun 26
Revision Received:	23rd Jun 26
Accepted:	26th Jun 26

Editor: William Teale

Transaction Report:

(Note: With the exception of the correction of typographical or spelling errors that could be a source of ambiguity, letters and reports are not edited. Depending on transfer agreements, referee reports obtained elsewhere may or may not be included in this compilation. Referee reports are anonymous unless the Referee chooses to sign their reports.)

The reviewers' comments and authors' responses are not available with this article, as the initial review process took place with another journal.

1st Editorial Decision 1st Aug 2025

Dear Thomas,

Thank you for transferring your manuscript entitled 'Vacuolar protein sorting-associated protein 1 (Vps1) has membrane constricting and severing1 abilities required for endosomal protein trafficking' to the EMBO Journal.

I would like formally to invite you to submit a revised version of the manuscript, addressing the comments of all three reviewers. I think it would be a good idea for us to discuss the planned revisions over Zoom next week; please let me know when you are available. I should add that it is EMBO Journal policy to allow only a single round of revision, and acceptance of your manuscript will therefore depend on the completeness of your responses in this revised version.

When preparing your letter of response to the referees' comments, please bear in mind that this will form part of the Review Process File, and will therefore be available online to the community. For more details on our Transparent Editorial Process, please visit our website: https://www.embopress.org/page/journal/14602075/authorguide#transparentprocess

We generally allow three months as standard revision time. As a matter of policy, competing manuscripts published during this period will not negatively impact on our assessment of the conceptual advance presented by your study. However, we request that you contact the editor as soon as possible upon publication of any related work, to discuss how to proceed. Should you foresee a problem in meeting this three-month deadline, please let us know in advance and we may be able to grant an extension.

Thank you for the opportunity to consider your work for publication. I look forward to your revision.

Best wishes,

William

William Teale, PhD
Editor
The EMBO Journal
w.teale@embojournal.org

When submitting your revised manuscript, please carefully review the instructions below and include the following items:

1) a .docx formatted version of the manuscript text (including legends for main figures, EV figures and tables). Please make sure that the changes are highlighted to be clearly visible.

2) individual production quality figure files as .eps, .tif, .jpg (one file per figure).

3) a .docx formatted letter INCLUDING the reviewers' reports and your detailed point-by-point response to their comments. As part of the EMBO Press transparent editorial process, the point-by-point response is part of the Review Process File (RPF), which will be published alongside your paper.

4) a complete author checklist, which you can download from our author guidelines (https://wol-prod-cdn.literatumonline.com/pb-assets/embo-site/Author Checklist%20-%20EMBO%20J-1561436015657.xlsx). Please insert information in the checklist that is also reflected in the manuscript. The completed author checklist will also be part of the RPF.

5) Please note that all corresponding authors are required to supply an ORCID ID for their name upon submission of a revised manuscript.

6) We require a 'Data Availability' section after the Materials and Methods. Before submitting your revision, primary datasets produced in this study need to be deposited in an appropriate public database, and the accession numbers and database listed under 'Data Availability'. Please remember to provide a reviewer password if the datasets are not yet public (see https://www.embopress.org/page/journal/14602075/authorguide#datadeposition). If no data deposition in external databases is needed for this paper, please then state in this section: This study includes no data deposited in external repositories. Note that the Data Availability Section is restricted to new primary data that are part of this study.

Note - All links should resolve to a page where the data can be accessed.

7) When assembling figures, please refer to our figure preparation guideline in order to ensure proper formatting and readability

in print as well as on screen:
http://bit.ly/EMBOPressFigurePreparationGuideline

Please remember: Digital image enhancement is acceptable practice, as long as it accurately represents the original data and conforms to community standards. If a figure has been subjected to significant electronic manipulation, this must be noted in the figure legend or in the 'Materials and Methods' section. The editors reserve the right to request original versions of figures and the original images that were used to assemble the figure.

8) For data quantification: please specify the name of the statistical test used to generate error bars and P values, the number (n) of independent experiments (specify technical or biological replicates) underlying each data point and the test used to calculate p-values in each figure legend. The figure legends should contain a basic description of n, P and the test applied. Graphs must include a description of the bars and the error bars (s.d., s.e.m.).

9) We would also encourage you to include the source data for figure panels that show essential data. Numerical data can be provided as individual .xls or .csv files (including a tab describing the data). For 'blots' or microscopy, uncropped images should be submitted (using a zip archive or a single pdf per main figure if multiple images need to be supplied for one panel). Additional information on source data and instruction on how to label the files are available at .

10) We replaced Supplementary Information with Expanded View (EV) Figures and Tables that are collapsible/expandable online (see examples in https://www.embopress.org/doi/10.15252/embj.201695874). A maximum of 5 EV Figures can be typeset. EV Figures should be cited as 'Figure EV1, Figure EV2" etc. in the text and their respective legends should be included in the main text after the legends of regular figures.

- For the figures that you do NOT wish to display as Expanded View figures, they should be bundled together with their legends in a single PDF file called *Appendix*, which should start with a short Table of Content. Appendix figures should be referred to in the main text as: "Appendix Figure S1, Appendix Figure S2" etc. See detailed instructions regarding expanded view here: .

- Additional Tables/Datasets should be labelled and referred to as Table EV1, Dataset EV1, etc. Legends have to be provided in a separate tab in case of .xls files. Alternatively, the legend can be supplied as a separate text file (README) and zipped together with the Table/Dataset file.

11) At EMBO Press we ask authors to provide source data for the main manuscript figures. Our source data coordinator will contact you to discuss which figure panels we would need source data for and will also provide you with helpful tips on how to upload and organize the files.

12) Our journal encourages inclusion of *data citations in the reference list* to directly cite datasets that were re-used and obtained from public databases. Data citations in the article text are distinct from normal bibliographical citations and should directly link to the database records from which the data can be accessed. In the main text, data citations are formatted as follows: "Data ref: Smith et al, 2001" or "Data ref: NCBI Sequence Read Archive PRJNA342805, 2017". In the Reference list, data citations must be labeled with "[DATASET]". A data reference must provide the database name, accession number/identifiers and a resolvable link to the landing page from which the data can be accessed at the end of the reference. Further instructions are available at .

13) In order to increase the reproducibility and reach of your work, The EMBO Journal includes a table of reagents that were used in the study. Please provide this along with your revisions.

Further instructions for preparing your revised manuscript:

Please make sure you upload a letter of response to the referees' comments together with the revised manuscript.

Please also check that the title and abstract of the manuscript are brief, yet explicit, even to non-specialists.

When assembling figures, please refer to our figure preparation guideline in order to ensure proper formatting and readability in print as well as on screen:
https://bit.ly/EMBOPressFigurePreparationGuideline
See also guidelines for figure legends: https://www.embopress.org/page/journal/14602075/authorguide#figureformat

At EMBO Press we ask authors to provide source data for the main manuscript figures. You will receive a separate email with instructions for providing source data with your revised manuscript, including how to upload and organize the files.

IMPORTANT: When you send the revision we will require
- a point-by-point response to the referees' comments, with a detailed description of the changes made (as a word file).
- a word file of the manuscript text.
- individual production quality figure files (one file per figure)

- a complete author checklist, which you can download from our author guidelines (https://www.embopress.org/page/journal/14602075/authorguide).
- Expanded View files (replacing Supplementary Information)
Please see out instructions to authors
https://www.embopress.org/page/journal/14602075/authorguide#expandedview
- a Reagents and Tools Table as part of the Methods section, which can be downloaded from our author guidelines (https://www.embopress.org/page/journal/14602075/authorguide#structuredmethods)

Please remember: Digital image enhancement is acceptable practice, as long as it accurately represents the original data and conforms to community standards. If a figure has been subjected to significant electronic manipulation, this must be noted in the figure legend or in the 'Materials and Methods' section. The editors reserve the right to request original versions of figures and the original images that were used to assemble the figure.

Further information is available in our Guide For Authors: https://www.embopress.org/page/journal/14602075/authorguide

We realize that it is difficult to revise to a specific deadline. In the interest of protecting the conceptual advance provided by the work, we recommend a revision within 3 months (30th Oct 2025). Please discuss the revision progress ahead of this time with the editor if you require more time to complete the revisions. Use the link below to submit your revision:

https://emboj.msubmit.net/cgi-bin/main.plex

Please see below for our response to the Reviewer's comments.

Response to Reviewer #1:

Overall, the paper is well written, the individual experiments are described with sufficient detail, and the text is easily readable. The characterization of VPS1 fission dynamics is novel, although VPS1 has been long characterized using different methods (e.g. Varlakhanova et al, Smaczynska-de Rooij et al, Suzuki et al and several others). The 3K patch has been described before (Smaczynska-de Rooij et al), but the phenylalanine-based interaction motif is novel. The in vitro assay system is elegant and highly functional to analyse membrane fission. However, it would be good to check other lipids that have been described in the literature (see specific comments). The proteomic analysis is interesting and has lots of potential, but the findings are not explored further. Overall, the manuscript does not use a lot of statistical analysis, instead, the authors show individual data points. This is nice as it shows the distribution of individual measurements. No statistics are used, in most cases, the differences between samples are massive and do not require formal statistics, as all data points are shown. It would be good to support the proteomics data with a volcano plot depicting enrichment and statistical robustness, right now it is only a long list of protein hits. In general, the in vitro characterization of VPS1 function on liposomes is robust and is the heart of the paper. While there are some minor issues with some of the conclusions, the experiments are robust and well-performed (see comments below). The rest of the paper is not as well developed, especially with the proteomics appearing to be an add-on. The proteomics appear only in the last figure, and while several proteins are identified, the authors do not really explore the function of VPS1 on the sorting of these proteins.

Phosphoinositide specificity of binding and membrane scission activity. I am wondering about the phosphoinositide specificity described, especially the selectivity for PtdIns3P. Endosomal tubules often undergo phosphoinositide conversion, e.g. from PtdIns3P to PtdIns4P (e.g. Ketel el al). Moreover, significant pools of PtdIns4,5P2 have been described at endosomes. In addition, Smaczynska-de Rooij et al described that Vps1 preferable binds to PtdIns4,5P2, with rather moderate binding to PtdIns3P. The authors cite this paper, but do not discuss the altered phosphoinositide preference, apart from stating that their assay is more sensitive. However, they never test PtdIns4,5P2. This is even more important, as they point to the 3K motif, which presumably drives charge-based binding, something that would also be provided by PtdIns4,5P. If binding by VPS1 to endosomes / vacuoles is indeed driven by the presence of both PS and PtdIns3P, it would be important to investigate if PtdIns4,5P2 is indeed not the major recruiting factor, e.g. by running both the binding and scission assays also with PtdIns4,5P2.

We thank the reviewer for this suggestion. We now report data for Vps1 functions on membranes containing other phosphoinositides (PI(4)P, PI(3,5)P_2 and PI(4,5,)P_2). These are described in the following text in the revised manuscript.

"Like mentioned earlier, endosomes also contain other phosphoinositides like PI(4)P, PI(3,5)P_2 and PI(4,5)P_2. We tested Vps1 functions on membranes containing 5 mol% of each of these phosphoinositides along with 15 mol% PS. PLiMAP showed similar levels of binding of Vps1 to these vesicles and robust fission on templates with these phosphoinositides (Fig. 1J-L). Thus, while phosphoinositides are important, Vps1 displays little specificity for phosphoinositides."

As to the phosphoinositide binding specificity and how does the 3K motif influence binding specificity, we also tested this mutants on templates with other phosphoinositides. These are described in the following text in the revised manuscript.

"Previous results from vesicle sedimentation assays have shown that the 3K motif is required for binding anionic lipids and certain phosphoinositides (Smaczynska-de Rooij *et al*, 2019). Thus, replacing the 3K motif with 3A reduced binding to anionic lipids and PI(4,5)P_2, while it caused no change in binding to PI(4)P. But surprisingly, this mutant showed better binding to PI(3)P than WT. Our results with PLiMAP indicate that loss of the 3K motif reduces binding to PI(3)P (Fig. 4D). Vps1 displays little specificity for phosphoinositides (Fig. 1K). Consistently, Δ3K was defective in binding other phosphoinositides (Fig. 4H). Thus, the 3K represents a generic phosphoinositide-binding motif (Fig. 4H)."

As to how our data on the 3K motif compares to previous work, we have re-worded the text to make it more explicit in the revised manuscript. These are described in the following text in the revised manuscript.

"Membrane binding of Vps1 was previously analyzed using vesicle sedimentation assays (Smaczynska-de Rooij *et al*, 2019). But these assays suffer from low sensitivity, necessitating the use of high concentrations (20 mol%) concentrations of phosphoinositides to monitor binding."

Scission assays: The authors use very elegant and highly controlled scission assays to determine the activity of VPS1 in vitro. The authors report a clear size dependence of membrane scission, with smaller tubules (~15-20 nm) being the preferred substrate. Is scission not working since these diameters are less effective in recruiting VPS1 to the tubules? Does it bind at all? It would be important to show this, as figure 2 only shows tubules with 20 nm diameter or smaller.

This is indeed an important mechanistic point and we have carried out additional experiments to address this comment. These are described in the following text in the revised manuscript.

"To understand why fission is so sensitive to the starting size of the tube, we analyzed membrane binding and scaffolding activities of Vps1 on tubes of different sizes. From data acquired while imaging GppNHp-bound Ax488-labeled Vps1 (Fig. 2D), we estimated the membrane density of Vps1 (ratio of Vps1 fluorescence to membrane fluorescence) and plotted this against the starting tube size. This plot shows a shallow decline in Vps1 density with increasing tube size (Fig. 2K), indicating that binding itself is partially sensitive to membrane curvature. The ability to constrict the tube should report the protein's tendency to self-assemble into a scaffold. To test if self-assembly was also sensitivity to membrane curvature, we estimated the size of the same tube before and after addition of Vps1 with GppNHp. Interestingly, tubes below ~30 nm radius were constricted to a defined size of ~20 nm radius while tubes wider than ~30 nm radius resisted constriction (Fig. 2L). Tubes above ~30 nm radius displayed sufficient protein density (Fig. 2K) but failed to undergo constriction (Fig. 2L), indicating that self-assembly is acutely sensitive to membrane curvature. The reason for fission to display sensitivity to tube size is therefore because of the inability of Vps1 to self-assemble on tubes wider than the ~30 nm critical limiting radius."

Determination of membrane constriction: This reviewer is not convinced that the authors can accurately and robustly determine a constriction from 20 to 7 nm using only fluorescence as read-out (Figure 2F,G). The observed reduced fluorescence at sites of VPS1 accumulation is robust and convincing. It is also clear that there is less fluorescence at areas of VPS1 accumulation, which would be in line with a constriction of the tubules. However, this reviewer is highly sceptical that fluorescence intensity can report diameters as accurately as claimed by the authors. The reported radius of the prefission intermediate (7.1 ± 1.8 nm) is far below the diffraction limit and inferred from fluorescence intensity measurements in a widefield setup.

These are all very good points and we thank the reviewer for taking the time. This concern is indeed valid if one were trying to estimate the distance between two diffraction-limited objects, which is not the case here. Our method estimates the evolution in size of already diffraction-limited objects. In fact, this prescription only works for objects below the diffraction limit and is based on a sound basis that corelates the brightness of a diffraction-limited object to its size using a calibration standard (DOI: 10.1038/nprot.2016.173). This has also been used by the Stamou group to estimate the size of diffraction-limited liposomes (DOI: 10.1529/biophysj.108.128819). Details of this methods are now described in the materials and methods section of the revised manuscript, which states the following.

"We calculated the membrane tube radii by utilizing the supported bilayer as an internal calibration standard. Briefly, we subtracted the background and measured the integrated fluorescence per unit area of the supported bilayer. We calculated the area of 1-µm tube segments from their integrated fluorescence and derived the radius assuming a cylindrical shape. Linear regression of the peak tube fluorescence against these calculated radii yielded a calibration slope. We divided all pixel values in the tube images by this calibration constant to generate radius maps."

The widefield set-up is in fact better as it collects maximum photons and improves signal-to-noise.

While the relative fluorescence intensity can reflect differences in membrane area – assuming strict labelling and imaging conditions

Tube sizes are estimated from membranes containing the lipid Texas Red-DHPE added to 1 mol%. Inadvertent errors in estimating the dye concentration and therefore in their brightness is taken care by estimating the calibration constant for every SMrT template preparation.

– the authors use maximum pixel intensity (at least according to Dar et al, 2017). This induces a significant variance, especially given that EM-CCDs have significant noise and EM-CCD gain is not always linear. The PSF integrates light over a volume much larger than the structure itself, and peak pixel values are subject to noise, focus, and subpixel alignment effects. The claimed precision (±1.8 nm SD) appears optimistic in the absence of an independent validation method such as electron microscopy or super-resolution imaging.

The calibration constant is arrived at by plotting the maximum pixel intensity against the estimated tube radius across a range of tubes of different sizes and linear regression analysis of this data . So we don't rely on a single point measurement but a trend which improves confidence.

The findings are as such robust, and therefore I recommend weakening this statement, and to rather emphasize the relative differences and trends supported by the data. If the authors want to accurately state and determine the prefission intermediate, additional methods (e.g. EM, SMLM) would need to be used.

But we appreciate the reviewer's apprehension and have accordingly toned down emphasis on absolute estimates of tube sizes. Instead, we emphasize that, (a) there is indeed a super-constricted intermediate that precedes fission, and (b) the super-constricted intermediate displays cycles of thinning and constriction, with amplitudes that are much larger than the error inherent in these measurements. These results are now reported in the Fig. 2 of the revised manuscript.

Figure 4: The identification of the 4F motif is exciting and important. It would be important to further validate that this is indeed a self-assembly motif, e.g. by gel filtrations or other assays. Alternatively, it would be nice to analyse interacting amino acids (based on the structural data (Varlakhanova et al)) and generate the corresponding mutations to corroborate the effect of the 4F motif for multimerization.

This is indeed an exciting mutant. We have now carried out SEC-MALS analysis of the Vps1 mutants. These are reported in Figs. 3 and 4 of the revised manuscript. These are described in the following text in the revised manuscript.

"SEC-MALS revealed that both Vps1(Δ4F) and Vps1(Δ3K) were largely tetramers (Fig. 4B). Vps1(Δ4F) and Vps1(Δ3K) eluted slightly later than Vps1 and displayed narrower profiles. This implies that Vps1 exists in a rapidly interconverting equilibrium between higher order oligomers and tetramers and that the 4F and 3K motifs partially contribute towards the formation of higher order oligomers. Vps1(ΔInsB) was however a dimer (Fig. 4B). This indicates that regions outside the 4F and 3K motifs in InsB engage in interactions that maintain Vps1 as tetramers. Deletion of the equivalent VD also converts the mitochondrial dynamin Drp1 into dimers (Clinton *et al*, 2016), indicating a shared role between InsB and VD in maintaining the quaternary structure of dynamins."

Unfortunately, the Vps1 structure does not resolve the Insert B region so the precise reasons why the 4F mutant is defective in self-assembly remains unclear.

Figure 5: The authors propose enhances vacuolar localization of Lem3 and Neo1 in response to VPS1 deletion. It is not clear how this value is derived – the images show a clear cytosolic labelling, and no labelling of the limiting membrane. The increased vacuolar localization could be derived from more cytosolic background. This is not a very robust phenotype, and a more detailed analysis is necessary. In the case of Neo1, the loss of vesicular localization could provide a more robust measurement.

The earlier data was acquired with widefield imaging, which we agree was difficult to interpret because of the cytosolic haze. We have now imaged all the yeast strains with a confocal spinning disk. These data are now included in the revised manuscript. The phenotype of a reduction in cytosolic punctate and an increase in

vacuolar localization of these puncta in the absence of Vps1 is now more apparent. This is even more clearer in the associated supplementary movies. Lem3 is still relatively hard to visualize because of its low copy number but the change in localization is clearly apparent with Neo1. Furthermore, the quantitative vacuolar localization assay brings out these differences clearly.

Response to Reviewer#2:

This manuscript focuses on the functional analysis of the yeast dynamin-related protein Vps1 essential for vacuolar protein sorting. The initial breakthrough described here is to identify the requirement of the lipid PI3P for the membrane binding, GTPase activity and membrane scission of Vps1. It is somewhat surprising to me that PI3P had not been tested previously in structural studies of Vps1-coated membrane tubules, but this insight now allows the authors to fully explore the importance of Vps1 in membrane scission of PVC-derived tubules. I thought the paper was well written and had no major changes to suggest. I have some minor queries and edits suggested below. For context I have expertise in structural biology of membrane trafficking, but I am not specifically an expert in the dynamin-related proteins or yeast cell biology.

Line 61. The manuscript refers to the "cargo-sorting trimer" of Vps35, Vps26 and Vps29? For the sake of accuracy, I think this should be rephrased as the core trimer is NOT the cargo selective part of the sorting machinery, or at least not on its own. It only binds to cargo via SNX-family adaptors like Snx3 (in yeast and humans) or possibly the Snx-BAR proteins Vps5-Vps17 in yeast, but it has never been explicitly shown.

Thanks for the clarification. We have accordingly changed sentence this to the following.

"This comprises of the Vps26/Vps29/Vps35 that manages cargo-sorting when in complex with the Vps5/Vps17 SNX-BARs that tubulate the membrane."

In the first results section, where the Vps1 membrane scission assays are established, it is noted that a bacterially expressed Vps1 is used. It would be good to have the full Coomassie-stained gel as a supplementary figure to show the purity of this protein. Ideally a gel filtration profile or other assay such as MALS would also be good to show the protein is pure and homogeneous.

We now show the purification gel in Fig. 1B. We also show SEC-MALS data for WT and the other mutants. As stated in the manuscript, we were unable to accurately size WT, likely because of its tendency to form higher-order self-assemblies, but have done so for the other mutants. These are now reported in Figs. 3 and 4 of the revised manuscript.

The ability of the endosomal PI3P lipid to promote Vps1 activity and membrane scission on supported membrane tubules is of course very striking, but if possible it would be good to see one or two more controls to assess if this is specific to PI3P. e.g. to perform the assays with PI4P, or PI(3,5)P2.

We thank the reviewer for this suggestion. We now report data for Vps1 functions on membranes containing other phosphoinositides (PI(4)P, PI(3,5)P_2 and PI(4,5,)P_2). These are described in the following text in the revised manuscript.

"Like mentioned earlier, endosomes also contain other phosphoinositides like PI(4)P, PI(3,5)P_2 and PI(4,5)P_2. We tested Vps1 functions on membranes containing 5 mol% of each of these phosphoinositides along with 15 mol% PS. PLiMAP showed similar levels of binding of Vps1 to these vesicles and robust fission on templates with these phosphoinositides (Fig. 1J-L). Thus, while phosphoinositides are important, Vps1 displays little specificity for phosphoinositides."

I may have missed this, but I couldn't find information on how the tubule radius was measured for Fig. 2? This would be useful to know considering that it is mentioned that the tubule widths are diffraction limited.

We have now include this in the materials and methods section of the revised manuscript, and it reads as follows.

"We calculated the membrane tube radii by utilizing the supported bilayer as an internal calibration standard. Briefly, we subtracted the background and measured the integrated fluorescence per unit area of the supported bilayer. We calculated the area of 1-µm tube segments from their integrated fluorescence and derived the radius assuming a cylindrical shape. Linear regression of the peak tube fluorescence against these calculated radii yielded a calibration slope. We divided all pixel values in the tube images by this calibration constant to generate radius maps."

Relatively minor comment, but visually it would be good to plot the vacuolar Vps10-mNG quantitation (Figs. 3D and 3G) on the same graph for direct comparison.

We have done so in Figs. 3B and 5C of the revised manuscript.

Minor points:
6. Line 26. "harbor" should be "harbors".
7. Line 39. "is" should be "are".
8. Line 39. "in the yeast" should be "in yeast" or possibly "in the yeast Saccharomyces cerevisiae".
9. Line 291. Fig. 4I should reference Fig. 4J.

Thank you for pointing these out. We have now rectified these.

10. Line 414. I would add the following refernece to the Daly et al., reference for the role of Retromer with Rab7 enrichment. Kvainickas A, Nägele H, Qi W, Dokládal L, Jimenez-Orgaz A, Stehl L, Gangurde D, Zhao Q, Hu Z, Dengjel J, De Virgilio C, Baumeister R, Steinberg F. Retromer and TBC1D5 maintain late endosomal RAB7 domains to enable amino acid-induced mTORC1 signaling. J Cell Biol. 2019 Sep 2;218(9):3019-3038. doi: 10.1083/jcb.201812110. Epub 2019 Aug 20. PMID: 31431476; PMCID: PMC6719456.

With the new and improved mass spec data reported in the revised manuscript, we have modified our interpretation and don't cite this reference for interpreting the Rab7 data. The raw mass spec data has been deposited to the PRIDE Archive under project accession PXD077915 (Token: 0njVcOCxx833).

Response to Reviewer #3:

The manuscript of Gopan et al. dissects the function of the yeast dynamin vacuolar protein sorting-associated protein 1 (Vps1) in membrane fission processes along the endosomal retromer pathway in molecular detail. The authors show that Vps1 binds to tubular membranes containing the endosomal lipids PS and PI(3)P and furthermore mediates their fission, which depends on Vps1 scaffolding and GTPase activity and on the radius of the membrane tube. Furthermore, the authors find that different motifs of the Insert B domain mediate PI(3)P binding and self-assembly of Vps1, which in turn is required for membrane fission. Finally, the authors demonstrate that Vps1 functions in endosomal sorting, as loss of Vps1 results in changes of the overall vacuole proteome. This is an insightful study addressing the detailed molecular function of Vps1 in endosomal sorting and membrane fission processes. The authors study purified Vps1 regarding membrane binding (PLiMAP), GTPase activity (GTPase assays) and membrane constriction/fission ability (Supported Membrane Templates). Furthermore, they use these assays to analyse previously identified as well as novel Vps1 mutants, revealing novel insights into PI(3)P binding and self-assembly of Vps1. In addition, the authors follow the localization of the retromer cargo Vps10 and Vps1 itself in VPS1 mutant strains, confirming their in vitro findings in vivo. By comparison of the vacuolar proteome of wildtype and vps1Δ cells, the authors confirm that Vps1 is required for correct endosomal sorting. Together, this study describes novel insights into the molecular function of Vps1 in endosomal sorting and fission processes. While I see the important mechanistic insights on Vps1 (Figures 1-4), I am not sure about the novelty of the endosomal in vivo data (Figure 5), which appears to be rather an add on. This is also apparent from the abstract, where this part is not well integrated. Which model do the authors then develop out of their data? Apart from this, the authors need to better control the PI(3)P-specific membrane binding of Vps1 and refine their study regarding statistical analysis, sample size and biological replicates. Furthermore, the authors need to improve the visualization of the proteomics data, clarify the contradictory results and underline the value of the last part (Figure 5) for the overall manuscript. My specific

points are listed below:

Major points:

In Figure 1, the authors use PliMAP assays to convincingly show that Vps1 binds PC:PS and more extensively PC:PS:PI(3)P vesicles, while binding to simple PC vesicles appears rather low. However, did the authors also test binding to PC:PI(3)P vesicles not containing PS? And thus, does the presence of PS and PI(3)P have an additive effect on the lipid binding property of Vps1? Furthermore, is the presence of PI(3)P alone sufficient for stimulating membrane binding, GTPase activity and fission capacity (also in regard to the binding analysis of Vps1 mutants in Figure 4B-C)?

Thanks for pointing this out. We describe the importance of PS in the revise manuscript. The text reads as follows.

"To evaluate the contribution of PS in the membrane, we analyzed binding and fission activities of Vps1 on PI(3)P-containing membranes without PS. Vps1 bound PC:PI(3)P vesicles to a similar extent as PC:PS vesicles (Fig. 1H). But fission was only observed on PI(3)P-containing membranes (Fig. 1I). This emphasizes the role of phosphoinositides in activating Vps1 functions. Furthermore, inclusion of PS in PI(3)P-containing membranes improves binding (Fig. 1H). Thus, phosphoinositides displayed on PS-containing membranes optimally activate Vps1 functions in membrane binding and fission."

In addition to PC:PI(3)P vesicles, the authors should further include liposomes containing another phospholipid than PI(3)P, e.g. PI(4,5)P2, to ensure the specificity of the observed effects for Vps1, in particular since previous sedimentation assays showed that mutation of the Vps1 "3K motif affects binding to PI(4,5)P2 but not PI(3)P lipids" (line 395-396) (Smaczynska-de Rooji et al., 2019).

We thank the reviewer for this suggestion. We now report data for Vps1 functions on membranes containing other phosphoinositides (PI(4)P, PI(3,5)P_2 and PI(4,5,)P_2). These are described in the following text in the revised manuscript.

"Like mentioned earlier, endosomes also contain other phosphoinositides like PI(4)P, PI(3,5)P_2 and PI(4,5)P_2. We tested Vps1 functions on membranes containing 5 mol% of each of these phosphoinositides along with 15 mol% PS. PLiMAP showed similar levels of binding of Vps1 to these vesicles and robust fission on templates with these phosphoinositides (Fig. 1J-L). Thus, while phosphoinositides are important, Vps1 displays little specificity for phosphoinositides."

As to the phosphoinositide binding specificity and how does the 3K motif influence binding specificity, we also tested this mutants on templates with other phosphoinositides. These are described in the following text in the revised manuscript.

"Previous results from vesicle sedimentation assays have shown that the 3K motif is required for binding anionic lipids and certain phosphoinositides (Smaczynska-de Rooij *et al*, 2019). Thus, replacing the 3K motif with 3A reduced binding to anionic lipids and PI(4,5)P_2, while it caused no change in binding to PI(4)P. But surprisingly, this mutant showed better binding to PI(3)P than WT. Our results with PLiMAP indicate that loss of the 3K motif reduces binding to PI(3)P (Fig. 4D). Vps1 displays little specificity for phosphoinositides (Fig. 1K). Consistently, Δ3K was defective in binding other phosphoinositides (Fig. 4H). Thus, the 3K represents a generic phosphoinositide-binding motif (Fig. 4H)."

As to how our data on the 3K motif compares to previous work, we have re-worded the text to make it more explicit in the revised manuscript. These are described in the following text in the revised manuscript.

"Membrane binding of Vps1 was previously analyzed using vesicle sedimentation assays (Smaczynska-de Rooij *et al*, 2019). But these assays suffer from low sensitivity, necessitating the use of high concentrations (20 mol%) of phosphoinositides to monitor binding."

The authors show that Vps1 scaffolds thin (15 nm radius) and thick (40 nm radius) tubules but mediates membrane fission only on the thinner tubes (Figure 2A-C). However, the sample size between the thin and

thick tubules varies significantly (from 2 to more than 70 analyzed tubes, see Figure legend). I guess the varying sample size is due to technical reasons (preparation of tubes with a defined radius). If this is the reason, the authors should at least mention this in the main text. Otherwise, they should equalize the sample size for thin and thick tubes.

This was indeed the case for the data set analyzed and reported in the original manuscript. We have now carried out additional analysis and extended the range of tube sizes analyzed (Fig. 2B). Stats on the numbers of tubes analyzed is reported in the legend to Fig. 2B.

Furthermore: What determines tube radius in vivo?

This is likely decided by the inherent property of the SNX-BAR proteins that tubulate the membrane to reach dimensions that are compliant for Vps1 to sever them. We state this in the text of the revised manuscript as follows.

"Fission dynamins form curved helical scaffolds around tubular membranes and their ability to do so is expected to be influenced by the topology or curvature of the tube (Kamerkar *et al*, 2018; Ford & Chappie, 2019; Jimah & Hinshaw, 2019; Khurana *et al*, 2023; Sarkar & Pucadyil, 2025). SNX-BAR proteins in the retromer pathway tubulate membranes and their ability to define membrane curvature could regulate the binding and self-assembly of Vps1, which in turn could determine the success of fission reactions. We therefore systematically analyzed how Vps1 functions are regulated by membrane curvature."

And later in Figure 2F: Why does Vps1 behave unlike mammalian dynamins and does not cause immediate fission as one would expect for an active enzyme? The authors should discuss these points.

We do so now in the revise manuscript. These are described in the following text in the revised manuscript.

"Remarkably, the super-constricted intermediate seen on thin tubes persisted for a long time before the tube underwent fission (Movie S3). This is quite unlike how the mammalian endocytic and mitochondrial dynamins function, where the GTPase-induced constriction leads to immediate fission (Dar & Pucadyil, 2017; Pemberton *et al*, 2025). The long-lived super-constricted intermediate is reminiscent of our earlier observations with a mutant of the mammalian endocytic dynamin where the membrane-binding Pleckstrin-homology domain (PHD) was replaced with an unstructured lipid-binding linker (Dar & Pucadyil, 2017). Residues in the PHD insert into the membrane and facilitate fission (Baratam *et al*, 2021; Khurana *et al*, 2023). Instead of the PHD, Vps1 contains an unstructured B-insert (see below) and the slower snapping of the tube could reflect the nature of interactions between residues in the B-insert and the membrane."

None of the graphs in Figure 1 and 2 contains statistical analysis (p-values). For Figure 3, 4 and 5, $p<0.0001$ is generally mentioned in the figure legends. However, significant differences should be indicated in the graphs. Furthermore, all in vitro experiments should be done in three independent replicates.

We have now carried out additional analyses. Data from all in vitro experiments is from 3 or more independent repeats. These details are reported in the respective figure legends.

For the mutant analysis in Figure 3E-L and Figure 4, the reader would benefit from a schematic overview of the Vps1 domain organization. I suggest adding a scheme after Figure 3D.

Thanks for this suggestion. We have now included this schematic in Fig. 1A of the revised manuscript.

In Figure 5, the authors compare the vacuolar proteome of wildtype and vps1Δ cells in a quantitative mass spectrometry approach and conclude that Vps1 is critical for maintaining the vacuolar proteome. First, the authors need to visualize the proteomic data in a plot that resembles an established standard plot in the field (e.g. volcano plot). Based on the plot shown in Figure 5A, the reader cannot comprehend later explanations of enrichment/de-enrichment of proteins in/at the vacuole. In addition, the supplementary tables (Table S1-S3) need to be shown in a way that the reader can follow the enrichment/de-enrichment of single proteins.

We have re-done the mass spec analysis. Please see the data reported in Fig. 6 in the revise manuscript.

The raw mass spec data has been deposited to the PRIDE Archive under project accession PXD077915 (Token: 0njVcOCxx833).

However, my main point regarding Figure 5 is that the shown microscopy images in Figure 5B, D, F and H do not fit the proteomics data and the corresponding quantifications. One example: The authors report that Ybt1 is depleted in the vacuolar proteome of vps1Δ cells. Based on the shown images in Figure 5B, Ybt1 similarly localizes to vacuoles in wt and vps1Δ cells, while the quantification in Figure 5C shows a slight reduction in the vacuolar localization of Ybt1. This contradiction does not only apply for Ybt1 but also for the shown Zrc1, Neo1 and Lem3 localization. I assume that either the authors did not choose representative microscopy images or, more likely, the observed enrichments/de-enrichments of Ybt1, Zrc1, Neo1 and Lem3 in the vacuolar proteome are only minor and not of biological significance. The authors should clarify this point. Furthermore, it is not clear why the authors choose Zrc1 for their microscopy analysis because this protein is not mentioned to be depleted in the text.

These concerns are valid and could have arisen from our selection of representative images. But independently, we have now replaced the entire yeast cell imaging data, which was earlier acquired using widefield microscopy, with those acquired on a spinning disk confocal. We have also substantially changed our interpretation of the mass spec data. We request the reviewer to see these changes, which are highlighted in the revised manuscript.

One more general point regarding Figure 5: I am wondering about the contribution of this figure to the overall manuscript if the authors later do not follow up on their candidate hits. Right now, they can only state that loss of VPS1 leads to enrichment of retromer cargo, which was known before, and depletion of many other proteins at the vacuole (which they cannot explain).

Again, our initial assessment of the mass spec data and its significance was quite preliminary. However, we believe this data is an important contribution to the manuscript and have carried out extensive new experiments to emphasize how the mass spec data serves as an important starting point to understand the repertoire of cargo that become mis-sorted in the absence of Vps1. The reviewer may re-assess after going over the entire revised Fig. 6. The raw mass spec data has been deposited to the PRIDE Archive under project accession PXD077915 (Token: 0njVcOCxx833).

Minor issues:

1. In Figure 3A-C, the authors show that loss of VPS1 results in vacuolar localization of Vps10, which can be rescued by re-introducing a VPS1* transgene. In addition to the quantification of vacuolar Vps10-mNG, the authors could include the number/fluorescence intensity of Vps1 dots per cell since Vps1 dot localization in the VPS1* strain looks slightly different compared to wildtype cells.

This again has to do with the choice of our representative images and the method of imaging live yeast cells. The new spinning disk images clearly bring out distinct phenotypes in the absence of Vps1. Estimating the number of fluorescent puncta would have been an option, except that it's quite difficult to achieve this in a high-throughput and unbiased format manner.

We also acknowledge that the VPS1* does not completely rescue the vps1delta phenotype with respect to VPS10 localization to the vacuole. These are described as follows in the revised manuscript.

"Vps10-mNG distribution appeared punctate and largely distinct from the vacuole in *VPS1** cells like that seen in WT, implying rescue of the Vps10 mis-sorting defect (Fig. 3A, Movie S6). To quantitatively analyze mis-sorting, we used an image analysis routine that estimates Vps10-mNG signals within a mask generated from the Vph1-mSc signal. When applied to a large population of cells, this analysis revealed high vacuolar levels of Vps10-mNG in *vps1Δ* than WT. Vps10-mNG levels were closer to WT than *vps1Δ* in *VPS1** (Fig. 3B). The lack of complete rescue could be because of the additional ALFA tag at the C-terminus in the *VPS1** transgene. Together, these results establish a quantitative vacuolar localization assay to monitor Vps1 functions."

2. In Figure 3H, the authors should add a quantification of the membrane binding similar to Figure 1B.

This is now reported in Fig. 3E of the revised manuscript.

In Figure 3J and 4E, the authors should add fluorescence plots similar to Figure 2E to better visualize the constriction mediated by Vps1(K42A) and Vps1(Δ4F).

This is now shown in Figs. 3H-K and Figs. 4F in the revised manuscript.

In addition, did the authors check the protein stability of all purified Vps1 mutants, e.g. by SEC?

We have now carried out SEC-MALS analysis of the mutants and the data is shown in Figs. 3 and 4 of the revised manuscript. Please see the associated text in the results section that explains these results.

3. In the Methods section, the authors describe that they wash and image yeast cells in DPBS (line 544ff). Can the authors be sure that treatment and imaging of yeast cells in a salt-containing buffer does not affect the localization of any of their investigated proteins as well as vacuole morphology?

This is a valid concern and our new spinning disk imaging data has been acquired in nutrient-containing synthetic complete medium (SCM).

4. The authors should add a molecular model for the proposed Vps1 function in endosomal protein sorting. They could e.g. visualize their novel findings regarding the contribution of the Insert B domain towards Vps1 function.

We will pass on this suggestion. Showing these details across molecular to macroscopic length scales is not easy. Plus several papers, like Suzuki et al. (DOI 10: e69883), which we cite here have a cartoon figure of this kind.

Response to Reviewer #4:

In this manuscript by Gopan et al., the authors biochemically assessed the capacity of Vps1 to bind and sever membrane tubules. Using the PLiMAP methodology, they demonstrate that Vps1 can strongly bind to PI(3)P containing membranes and show that these membranes stimulate Vps1 GTPase activity. Using pre-formed membrane tubules, the authors demonstrate that Vps1 is sufficient to cause tubule fission. Through biochemical structure-function and in vivo analyses, they identify specific regions required for membrane binding or fission and characterize their impact on cargo sorting. Finally, they perform quantitative mass spectrometry on WT and Vps1 mutant vacuoles, identifying a large spectrum of improperly localized proteins. Altogether, the biochemical findings are of high quality, and the presented data nicely support the conclusions. The use of SMrTs allowed for a detailed definition of Vps1 recruitment and activity determinants. The validation of biochemical findings in vivo also effectively demonstrates the importance of the observed biochemical effects, broadening the scope of the study. However, it was felt that the proteomic dataset was suboptimal and that statistical determinants were not sufficiently included in data interpretation.

Major points

To this reviewer, the weaker aspect of the manuscript resides in the MS analysis of vacuolar content in WT and ΔVps1 yeast. Here are the aspects that should be improved or modified to adequately support the claims made based on that data. The biggest concern for the reviewer is that, from the presented data, it looks like the statistical significance of differentially enriched proteins was not considered in the analysis. Fig. 5A and the text focus on fold changes, but not on the statistical differences seen. According to Table S1, 130 proteins were significantly modulated. To the reviewer, these should be the ones analysed. While Ybt1 showed statistical enrichment, Zrc1, Neo1, and Lem3 did not show statistical differences, which makes it difficult to appreciate the data presented in Fig. 5D to I. The lack of statistical significance could result from the fact that only two biological replicates were done. Thus, performing the experiment in triplicate could strengthen the data. However, if all six technical replicates were incorporated into the statistical analysis, it might suggest a significant amount of variability between the two biological replicates and between MS runs, thus reducing the confidence in the generated data. To the reviewer, showing the correlation between the biological repeats, along with a detailed description of how the statistical analyses were performed, would be necessary. Currently, there is too much uncertainty in the provided data to support the conclusions drawn from Figure 5

with confidence. Another means to show the robustness of their quantitative proteome would be to swap labels between biological repeats. Hence, performing a third repeat and labeling the proteins with the other label would strengthen the established ratios and the robustness of their MS data.

We thank the reviewer for these comments and acknowledge all the limitations of the previous mass spec analysis. But this discovery aspect is quite important since it provides a repertoire of cargo that utilize Vps1 for their trafficking. We have therefore carried out an altogether new mass spec analysis using the DIA method. All of this data constitutes the new Fig. 6 of the revised manuscript. We request the reviewer to go over this and let us know how we fared, both in terms of conducting the experiments, the mass spec analysis and our interpretation of the results. The raw mass spec data has been deposited to the PRIDE Archive under project accession PXD077915 (Token: 0njVcOCxx833).

Vacuoles from Vps1 mutant yeasts are quite different from those of WT cells. Thus, it is possible that they were not efficiently purified for the proteomic analyses. While I understand that a similar quantity of proteins was used for the MS, other organelles or contaminants may have been present in the ΔVps1 vacuole fraction, potentially causing the observed large number of proteins with low abundance. While the increase in Vps10 argues against this, the authors should provide some data demonstrating that their vacuole purification protocol works equally well in WT and mutant yeasts.

We have also wondered about this. To address this concern, we provide additional data regarding vacuoles isolated from WT and Vps1 delta cells. We state this in the text of the revised manuscript as follows.

"Vacuoles isolated on a density gradient from *vps1Δ* cells showed a similar banding pattern and gross morphology as those isolated from WT cells (Fig. 6A,B). This encouraged us to go forward with their processing for quantitative comparative proteomics."

Another unrelated point that would broaden the scope of the study would be to increase the number of lipids tested in Figure 1. The retromer interacts with Rab7/Ypt7 in both metazoans and yeast. Since Rab7/Ypt7 is mainly associated with late endosomal compartments, one would think that PI(3,5)P2 would be the major lipid present at sites of retromer recruitment. Hence, comparing PI(3)P and PI(3,5)P2 for Vps1 binding could reveal different kinetics for Vps1 and would improve its mechanistic understanding.

We thank the reviewer for this suggestion. We now report data for Vps1 functions on membranes containing other phosphoinositides (PI(4)P, PI(3,5)P_2 and PI(4,5,)P_2). These are described in the following text in the revised manuscript.

"Like mentioned earlier, endosomes also contain other phosphoinositides like PI(4)P, PI(3,5)P_2 and PI(4,5)P_2. We tested Vps1 functions on membranes containing 5 mol% of each of these phosphoinositides along with 15 mol% PS. PLiMAP showed similar levels of binding of Vps1 to these vesicles and robust fission on templates with these phosphoinositides (Fig. 1J-L). Thus, while phosphoinositides are important, Vps1 displays little specificity for phosphoinositides."

Minor points

From the methodology, it seems that the number of peptides was used as a filtering tool. While I understand that a minimum of two independent peptides was used to identify proteins in this study, it is unclear if the number of peptides from a given protein was also used for the differential comparisons and statistical analyses performed. Smaller proteins will have a lower number of peptides compared to larger proteins; thus, the ratio of heavy to light peptides for each protein is, to the reviewer, more appropriate than using the number of peptides for statistical analyses. This should be clarified in the methodology.

Again, please go over the new mass spec results where we have taken in account all of these procedural and statistical aspects. The raw mass spec data has been deposited to the PRIDE Archive under project accession PXD077915 (Token: 0njVcOCxx833).

A limitation of the MS approach used is that the MS analysis was performed in Information-Dependent acquisition (or DDA) rather than in Data-Independent Acquisition. This limits the number of spectra that can be

characterized. Hence, it is likely that proteins with low expression levels were missed. This limitation should be acknowledged and briefly discussed.

We thank the reviewer for this suggestion and accordingly performed a whole new mass spec data analysis based on DIA. These results constitute Fig. 6 of the revise manuscript. The raw mass spec data has been deposited to the PRIDE Archive under project accession PXD077915 (Token: 0njVcOCxx833).

1st Revision - Editorial Decision 16th Jun 2026

Dear Dr. Pucadyil,

We have now received re-review reports from three referees, which I have included below. As you will see, you have addressed their concerns satisfactorily; however, I would like you to consider addressing their remaining points raised by reviewer #3; these suggestions centre on crystalising your data into a model and improving the format in which some of the data are presented. Before I can finally accept the manuscript, there are some remaining editorial points which need to be addressed. In this regard would you please:

- rename the conflict of interest statement as the 'Disclosure and competing interests statement',
- acknowledge employment in a biotech compant in this statement,
- remove the AC/CrediT section from the manuscript text,
- include a callout for Fig. 6H,
- rename EV figures as Figure EV1-EV3 instead of Figure S1-S3 (along with the appropriate callouts in the manuscript text),
- update source file names, titles, legends and manuscript callouts to Dataset EV1-EV8 instead of Table S1-S8, legends should be removed from the main manuscript text and uploaded as a separate tab/sheet in each Excel file,
- provide exact p values in the legends of figures 1D, E; 3B, F; 4D, E, H,
- indicate the statistical test used for data analysis in the legends of figures 6C, D-F, K, L,
- correct mismatch between the annotated p values in the figure legend and the annotated p values in the figure file in figures 3E,
- rename the "Materials and Methods" to "Methods"
- rename Tables S9-S10 to Table EV1-EV2 with the appropriate callouts. Table legends should be removed from the main manuscript and placed above the tables in each Excel file,
- rename movies to Movie EV1-EV22 with the corresponding callouts, and the legends should be removed from the manuscript and zipped with the appropriate movie file, and
- use the following section order: Title page - Abstract - Introduction - Results - Discussion - Methods - Data Availability - Acknowledgements - Disclosure and Competing Interests Statement - References - Figure Legends - Table(s) - Expanded View Figure Legends.

We include a synopsis of the paper (see http://emboj.embopress.org/). Please provide me with a general summary image of exactly 550x300 pixels (in a jpeg/png/tiff format) along with a two sentence statement and 3-5 bullet points that capture the key findings of the paper.

I am looking forward to receiving your revised manuscript.

EMBO Press is an editorially independent publishing platform for the development of EMBO scientific publications.

Best wishes,

William

William Teale, PhD
Editor
The EMBO Journal
w.teale@embojournal.org

Instructions for preparing your revised manuscript:

Read our guidance for manuscript revisions and related editorial policies: https://link.springer.com/journal/44318/submission-guidelines#cms-Revised-submissions

Please also check that the title and abstract of the manuscript are brief, yet explicit, even to non-specialists.

When assembling figures, please refer to our figure preparation guideline in order to ensure proper formatting and readability in print as well as on screen:
https://media.springernature.com/original/springer-cms/rest/v1/content/27825798/data/v1

IMPORTANT: When you send the revision we will require
- a point-by-point response to the referees' comments, with a detailed description of the changes made (as a word file).

- a word file of the manuscript text.
- individual production quality figure files (one file per figure)
- a complete author checklist
- Expanded View files (replacing Supplementary Information)
- a Reagents and Tools Table as part of the Methods section

Please remember: Digital image enhancement is acceptable practice, as long as it accurately represents the original data and conforms to community standards. If a figure has been subjected to significant electronic manipulation, this must be noted in the figure legend or in the 'Methods' section. The editors reserve the right to request original versions of figures and the original images that were used to assemble the figure.

We realize that it is difficult to revise to a specific deadline. In the interest of protecting the conceptual advance provided by the work, we recommend a revision within 3 months (14th Sep 2026). Please discuss the revision progress ahead of this time with the editor if you require more time to complete the revisions. Use the link below to submit your revision:

https://emboj.msubmit.net/cgi-bin/main.plex

Referee #1:

This manuscript by Gopan et al. directly tested the role of Vps1 in membrane fission and its impact on cargo trafficking in vivo. The authors used in vitro reconstitution assays to identify specific residues/mechanisms involved in Vps1 membrane binding and fission. They also established a catalogue of vacuolar proteins affected in Vps1 mutant cells. Thus, by using novel approaches, this study describes a new in vitro mechanism for Vps1 function, with further support from in vivo imaging and functional data. These findings are significant and pertinent to a wide range of researchers.

In this revised version, following a previously unsuccessful submission at another journal, the authors added a significant and impressive number of new experiments to address all of the reviewers' comments. My concerns were primarily focused on the mass spectrometry aspects of the manuscript, and I commend the authors for performing a completely new quantitative mass spectrometry analysis. They also expanded the functional characterization of Vps1 by performing assays using a broader range of phosphoinositides. Through these new experiments, the authors have addressed all of my concerns, and I am happy to recommend the manuscript for publication.

Referee #2:

The authors have thoroughly revised the manuscript and addressed all my comments convincingly. The additional experiments, in particular the additional analysis of phosphoinositide specificity, as well as the curvature-dependent constriction, and SEC to test Vps1 oligomerization, all substantially strengthen the manuscript. I also appreciate the more cautious presentation of the fluorescence-based measurements of tubule constriction, as well as the thorough discussion of this in the rebuttal.
I have no further comments and support publication of the manuscript in its present form.

Referee #3:

The manuscript of Gopan et al. Has been transferred and has been revised based on my previous comments. I asked in particular an improvement of the presentation, mutant analysis and proteomics. This has all been done thoroughly, though I find the discussion of the data too extensive as they basically observe many proteins enriched and analyze only the vps1 deletion mutant in their data set. Here a presentation similar to Eising et al. would be recommended to guide the reader through the data on what is enriched on vacuoles from the vps1 deletion compared to wild-type. I guess this is the fold-change difference they describe here.

What I am still missing is a final model, which would help to guide the reader through the study. I recommend that the authors include this in their final submission. Overall, the manuscript has been strongly improved. I have no further experimental suggestions.

Response to Referee #1:

This manuscript by Gopan et al. directly tested the role of Vps1 in membrane fission and its impact on cargo trafficking in vivo. The authors used in vitro reconstitution assays to identify specific residues/mechanisms involved in Vps1 membrane binding and fission. They also established a catalogue of vacuolar proteins affected in Vps1 mutant cells. Thus, by using novel approaches, this study describes a new in vitro mechanism for Vps1 function, with further support from in vivo imaging and functional data. These findings are significant and pertinent to a wide range of researchers.

In this revised version, following a previously unsuccessful submission at another journal, the authors added a significant and impressive number of new experiments to address all of the reviewers' comments. My concerns were primarily focused on the mass spectrometry aspects of the manuscript, and I commend the authors for performing a completely new quantitative mass spectrometry analysis. They also expanded the functional characterization of Vps1 by performing assays using a broader range of phosphoinositides. Through these new experiments, the authors have addressed all of my concerns, and I am happy to recommend the manuscript for publication.

Thank you for your time and helpful comments in reviewing our manuscript. Your feedback has greatly enhanced its scope and content.

Response to Referee #2:

The authors have thoroughly revised the manuscript and addressed all my comments convincingly. The additional experiments, in particular the additional analysis of phosphoinositide specificity, as well as the curvature-dependent constriction, and SEC to test Vps1 oligomerization, all substantially strengthen the manuscript. I also appreciate the more cautious presentation of the fluorescence-based measurements of tubule constriction, as well as the thorough discussion of this in the rebuttal.
I have no further comments and support publication of the manuscript in its present form.

Thank you for your time and helpful comments in reviewing our manuscript. Your feedback has greatly enhanced its scope and content.

Response to Referee #3:

The manuscript of Gopan et al. Has been transferred and has been revised based on my previous comments. I asked in particular an improvement of the presentation, mutant analysis and proteomics. This has all been done thoroughly, though I find the discussion of the data too extensive as they basically observe many proteins enriched and analyze only the vps1 deletion mutant in their data set. Here a presentation similar to Eising et al. would be recommended to guide the reader through the data on what is enriched on vacuoles from the vps1 deletion compared to wild-type. I guess this is the fold-change difference they describe here.

What I am still missing is a final model, which would help to guide the reader through the study. I recommend that the authors include this in their final submission. Overall, the manuscript has been strongly improved. I have no further experimental suggestions.

We appreciate your thorough review and helpful comments, which have greatly enhanced the manuscript's scope. We have modified the text and synthesized the mass spectrometry results into a new model, making our findings much more accessible to the reader.

Dear Prof. Pucadyil,

I am pleased to inform you that your manuscript has been accepted for publication in the EMBO Journal.

Many congratulations to you and your team!

Your manuscript will be processed for publication by EMBO Press. It will be copy edited and you will receive page proofs prior to publication. Please note that you will be contacted by Springer Nature Author Services to complete licensing and payment information.

You may qualify for financial assistance for your publication charges - either via a Springer Nature fully open access agreement or an EMBO initiative. Check your eligibility: https://link.springer.com/journal/44318/how-to-publish-with-us

Should you be planning a Press Release on your article, please notify the production team via this form in order to coordinate publication and release dates: https://forms.cloud.microsoft/e/eH4A8JVP6g

If you have any questions, please do not hesitate to contact the Editorial Office. Thank you for your contribution to The EMBO Journal.

Yours sincerely,

William Teale

William Teale, PhD
Editor
The EMBO Journal
w.teale@embojournal.org

*****
Please note that it is The EMBO Journal policy for the transcript of the editorial process (containing referee reports and your response letters) to be published as an online supplement to each paper. If you should prefer removal of any referee-only figures included in the point-by-point response(s), e.g. because they may still be used for future publication or because they have been reproduced from published work by others, please do let us know immediately via response email.
More information is available here: https://link.springer.com/partners/embo-press/editorial-policies#Peer%20review

** Click here to be directed to your login page: https://emboj.msubmit.net

EMBO Press Author Checklist

Corresponding Author Name: Thomas Pucadyil	
Journal Submitted to: The EMBO Journal	
Manuscript Number: EMBOJ-2025-122009	

USEFUL LINKS FOR COMPLETING THIS FORM
The EMBO Journal - Author Guidelines
EMBO Reports - Author Guidelines
Molecular Systems Biology - Author Guidelines
EMBO Molecular Medicine - Author Guidelines

Reporting Checklist for Life Science Articles (updated January
This checklist is adapted from Materials Design Analysis Reporting (MDAR) Checklist for Authors. MDAR establishes a minimum set of requirements in transparent reporting in the life sciences (see Statement of Task: 10.31222/osf.io/9sm4x). Please follow the journal's guidelines in preparing your
Please note that a copy of this checklist will be published alongside your article.

Abridged guidelines for figures
1. Data
The data shown in figures should satisfy the following conditions:
- the data were obtained and processed according to the field's best practice and are presented to reflect the results of the experiments in an accurate and unbiased manner.
- ideally, figure panels should include only measurements that are directly comparable to each other and obtained with the same assay.
- plots include clearly labeled error bars for independent experiments and sample sizes. Unless justified, error bars should not be shown for technical
- if n<5, the individual data points from each experiment should be plotted. Any statistical test employed should be justified.
- Source Data should be included to report the data underlying figures according to the guidelines set out in the authorship guidelines on Data

2. Captions
Each figure caption should contain the following information, for each panel where they are relevant:
- a specification of the experimental system investigated (eg cell line, species name).
- the assay(s) and method(s) used to carry out the reported observations and measurements.
- an explicit mention of the biological and chemical entity(ies) that are being measured.
- an explicit mention of the biological and chemical entity(ies) that are altered/varied/perturbed in a controlled manner.
- the exact sample size (n) for each experimental group/condition, given as a number, not a range;
- a description of the sample collection allowing the reader to understand whether the samples represent technical or biological replicates (including how many animals, litters, cultures, etc.).
- a statement of how many times the experiment shown was independently replicated in the laboratory.
- definitions of statistical methods and measures:
  - common tests, such as t-test (please specify whether paired vs. unpaired), simple χ2 tests, Wilcoxon and Mann-Whitney tests, can be unambiguously identified by name only, but more complex techniques should be described in the methods section;
  - are tests one-sided or two-sided?
  - are there adjustments for multiple comparisons?
  - exact statistical test results, e.g., P values = x but not P values < x;
  - definition of 'center values' as median or average;
  - definition of error bars as s.d. or s.e.m.

Please complete ALL of the questions below.
Select "Not Applicable" only when the requested information is not relevant for your study.

Materials

Newly Created Materials	Information included in the manuscript?	In which section is the information available? (Reagents and Tools Table, Materials and Methods, Figures, Data Availability Section)
New materials and reagents need to be available; do any restrictions apply?	Yes	Reagents and Tools Table

Antibodies	Information included in the manuscript?	In which section is the information available? (Reagents and Tools Table, Materials and Methods, Figures, Data Availability Section)
For **antibodies** provide the following information:<br>- Commercial antibodies: RRID (if possible) or supplier name, catalogue number and or/clone number<br>- Non-commercial: RRID or citation	Yes	Materials and Methods

DNA and RNA sequences	Information included in the manuscript?	In which section is the information available? (Reagents and Tools Table, Materials and Methods, Figures, Data Availability Section)
**Short novel DNA or RNA including primers, probes**: provide the sequences.	Not Applicable	

Cell materials	Information included in the manuscript?	In which section is the information available? (Reagents and Tools Table, Materials and Methods, Figures, Data Availability Section)
**Cell lines:** Provide species information, strain. Provide accession number in repository **OR** supplier name, catalog number, clone number, and/**OR** RRID.	Yes	Reagent and Tools, Materials and Methods
**Primary cultures:** Provide species, strain, sex of origin, genetic modification status.	Not Applicable	
Report if the cell lines were recently **authenticated** (e.g., by STR profiling) and tested for mycoplasma contamination.	Not Applicable	

Experimental animals	Information included in the manuscript?	In which section is the information available? (Reagents and Tools Table, Materials and Methods, Figures, Data Availability Section)
**Laboratory animals or Model organisms:** Provide species, strain, sex, age, genetic modification status. Provide accession number in repository **OR** supplier name, catalog number, clone number, **OR** RRID.	Yes	Reagent and Tools, Materials and Methods, Acknowledgements
**Animal observed in or captured from the field:** Provide species, sex, and age where possible.	Not Applicable	
Please detail **housing and husbandry conditions**.	Not Applicable	

Plants and microbes	Information included in the manuscript?	In which section is the information available? (Reagents and Tools Table, Materials and Methods, Figures, Data Availability Section)
**Plants:** provide species and strain, ecotype and cultivar where relevant, unique accession number if available, and source (including location for collected wild specimens).	Not Applicable	
**Microbes:** provide species and strain, unique accession number if available, and source.	Not Applicable	

Human research participants	Information included in the manuscript?	In which section is the information available? (Reagents and Tools Table, Materials and Methods, Figures, Data Availability Section)
If collected and within the bounds of privacy constraints report on age, sex and gender or ethnicity for all study participants.	Not Applicable	

Core facilities	Information included in the manuscript?	In which section is the information available? (Reagents and Tools Table, Materials and Methods, Figures, Data Availability Section)
If your work benefited from core facilities, was their service mentioned in the acknowledgments section?	Yes	Acknowledgements

Design

Study protocol	Information included in the manuscript?	In which section is the information available? (Reagents and Tools Table, Materials and Methods, Figures, Data Availability Section)
If study protocol has been **pre-registered, provide DOI in the manuscript**. For clinical trials, provide the trial registration number **OR** cite DOI.	Not Applicable	
Report the **clinical trial registration number** (at ClinicalTrials.gov or equivalent), where applicable.	Not Applicable	

Laboratory protocol	Information included in the manuscript?	In which section is the information available? (Reagents and Tools Table, Materials and Methods, Figures, Data Availability Section)
Provide DOI OR other citation details if **external detailed step-by-step protocols** are available.	Yes	Materials and Methods (DOI:10.1038/nprot.2016.173, DOI: 10.1111/tra.12719, DOI: 10.1002/cpps.110.)

Experimental study design and statistics	Information included in the manuscript?	In which section is the information available? (Reagents and Tools Table, Materials and Methods, Figures, Data Availability Section)
Include a statement about **sample size** estimate even if no statistical methods were used.	Yes	Figure legends
Were any steps taken to minimize the effects of subjective bias when allocating animals/samples to treatment (e.g. **randomization procedure**)? If yes, have they been described?	Not Applicable	
Include a statement about **blinding** even if no blinding was done.	Not Applicable	
Describe **inclusion/exclusion criteria** if samples or animals were excluded from the analysis. Were the criteria pre-established?<br><br>If sample or data points were omitted from analysis, report if this was due to attrition or intentional exclusion and provide justification.	Not Applicable	
For every figure, are **statistical tests** justified as appropriate? Do the data meet the assumptions of the tests (e.g., normal distribution)? Describe any methods used to assess it. Is there an estimate of variation within each group of data? Is the variance similar between the groups that are being statistically compared?	Yes	Figure legends

Sample definition and in-laboratory replication	Information included in the manuscript?	In which section is the information available? (Reagents and Tools Table, Materials and Methods, Figures, Data Availability Section)
In the figure legends: state number of times the experiment was **replicated** in laboratory.	Yes	Figure legends
In the figure legends: define whether data describe **technical or biological replicates**.	Yes	Figure legends

Ethics

Ethics	Information included in the manuscript?	In which section is the information available? (Reagents and Tools Table, Materials and Methods, Figures, Data Availability Section)
Studies involving **human participants**: State details of **authority granting ethics approval** (IRB or equivalent committee(s), provide reference number for approval.	Not Applicable	
Studies involving **human participants**: Include a statement confirming that **informed consent** was obtained from all subjects and that the experiments conformed to the principles set out in the WMA Declaration of Helsinki and the Department of Health and Human Services Belmont Report.	Not Applicable	
Studies involving **human participants:** For publication of **patient photos**, include a statement confirming that consent to publish was obtained.	Not Applicable	
Studies involving experimental **animals**: State details of **authority granting ethics approval** (IRB or equivalent committee(s), provide reference number for approval. Include a statement of compliance with ethical regulations.	Not Applicable	
Studies involving **specimen and field samples:** State if relevant **permits** obtained, provide details of authority approving study; if none were required, explain why.	Not Applicable	

Dual Use Research of Concern (DURC)	Information included in the manuscript?	In which section is the information available? (Reagents and Tools Table, Materials and Methods, Figures, Data Availability Section)
Could your study fall under dual use research restrictions? Please check biosecurity documents and list of **select agents and toxins** (CDC): https://www.selectagents.gov/sat/list.htm	Not Applicable	
If you used a select agent, is the security level of the lab appropriate and reported in the manuscript?	Not Applicable	
If a study is subject to dual use research of concern regulations, is the name of the **authority granting approval and reference number** for the regulatory approval provided in the manuscript?	Not Applicable	

Reporting

The MDAR framework recommends adoption of discipline-specific guidelines, established and endorsed through community initiatives. Journals have their own policy about requiring specific guidelines and recommendations to complement MDAR.

Adherence to community standards	Information included in the manuscript?	In which section is the information available? (Reagents and Tools Table, Materials and Methods, Figures, Data Availability Section)
State if relevant guidelines or checklists (e.g., **ICMJE, MIBBI, ARRIVE, PRISMA**) have been followed or provided.	Not Applicable	
For **tumor marker prognostic studies,** we recommend that you follow the **REMARK** reporting guidelines (see link list at top right). See author guidelines, under 'Reporting Guidelines'. Please confirm you have followed these guidelines.	Not Applicable	
For **phase II and III randomized controlled trials**, please refer to the **CONSORT** flow diagram (see link list at top right) and submit the CONSORT checklist (see link list at top right) with your submission. See author guidelines, under 'Reporting Guidelines'. Please confirm you have submitted this list.	Not Applicable	

Data Availability

Data availability	Information included in the manuscript?	In which section is the information available? (Reagents and Tools Table, Materials and Methods, Figures, Data Availability Section)
Have **primary datasets** been deposited according to the journal's guidelines (see 'Data Deposition' section) and the respective accession numbers provided in the Data Availability Section?	Yes	Data Availability Section, Source Data
Were **human clinical and genomic datasets** deposited in a public access-controlled repository in accordance to ethical obligations to the patients and to the applicable consent agreement?	Not Applicable	
Are **computational models** that are central and integral to a study available without restrictions in a machine-readable form? Were the relevant accession numbers or links provided?	Not Applicable	
If publicly available data were reused, provide the respective **data citations in the reference list**.	Not Applicable	
