# Supplementary material for: Vps1 drives membrane constriction and fission necessary for endosomal protein sorting
Source: EMBO J. 2026 Jul 20;45(17):6115–38. doi: 10.1038/s44318-026-00855-4 (PMC13534707; doi:10.1038/s44318-026-00855-4)
Supplement: Supplementary file 2 [file 44318_2026_855_MOESM42_ESM.pdf]

Expanded View Figures

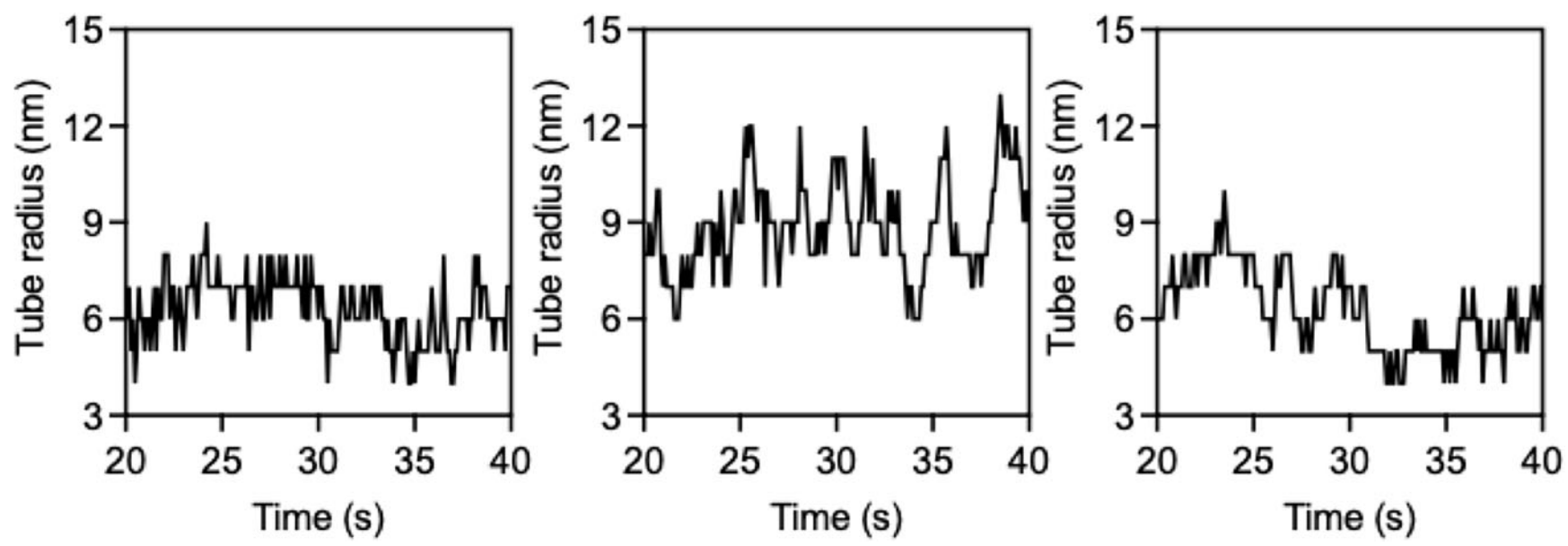

Figure EV1. Pathway to Vps1-catalyzed membrane fission.

Plots showing cycles of thinning and relaxation of the super-constricted intermediate on three tubes. Source data are available online for this figure.

© The Author(s)

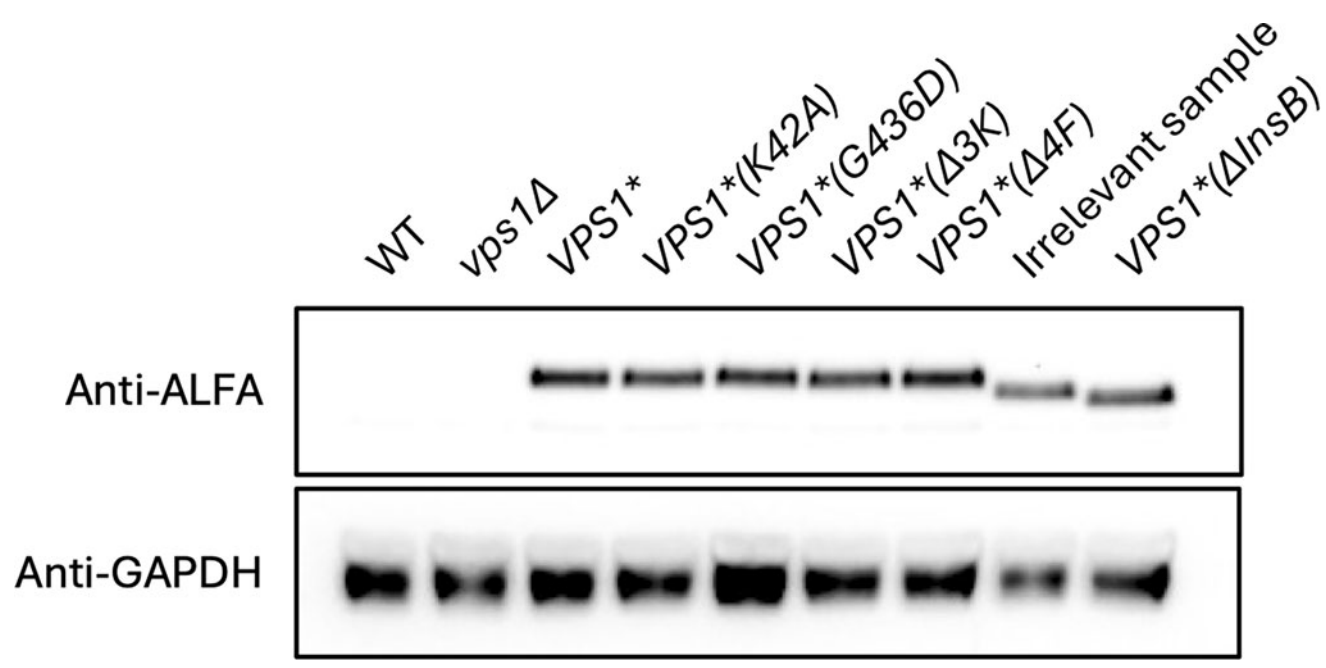

Figure EV2. Western blots of Vps1 and its mutants.

Lysates from the indicated strains were blotted with the anti-ALFA antibody. GAPDH served as the loading control. Source data are available online for this figure.

 © The Author(s)

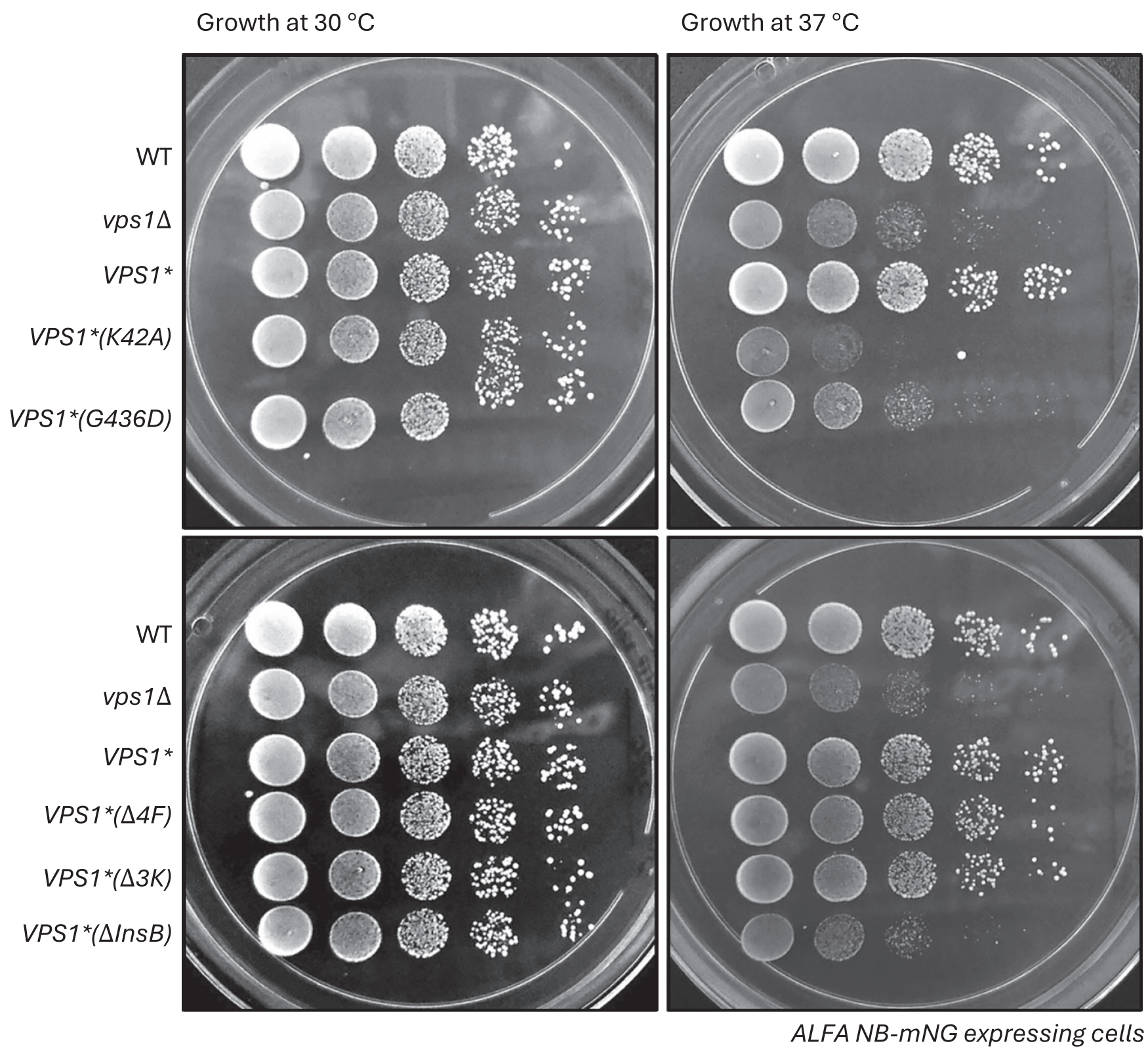

Figure EV3. Growth characteristics of *VPS1 strains.**

Spot assay comparing the growth of the indicated strains expressing the ALFA-NB-mNG at 30 and 37 °C.

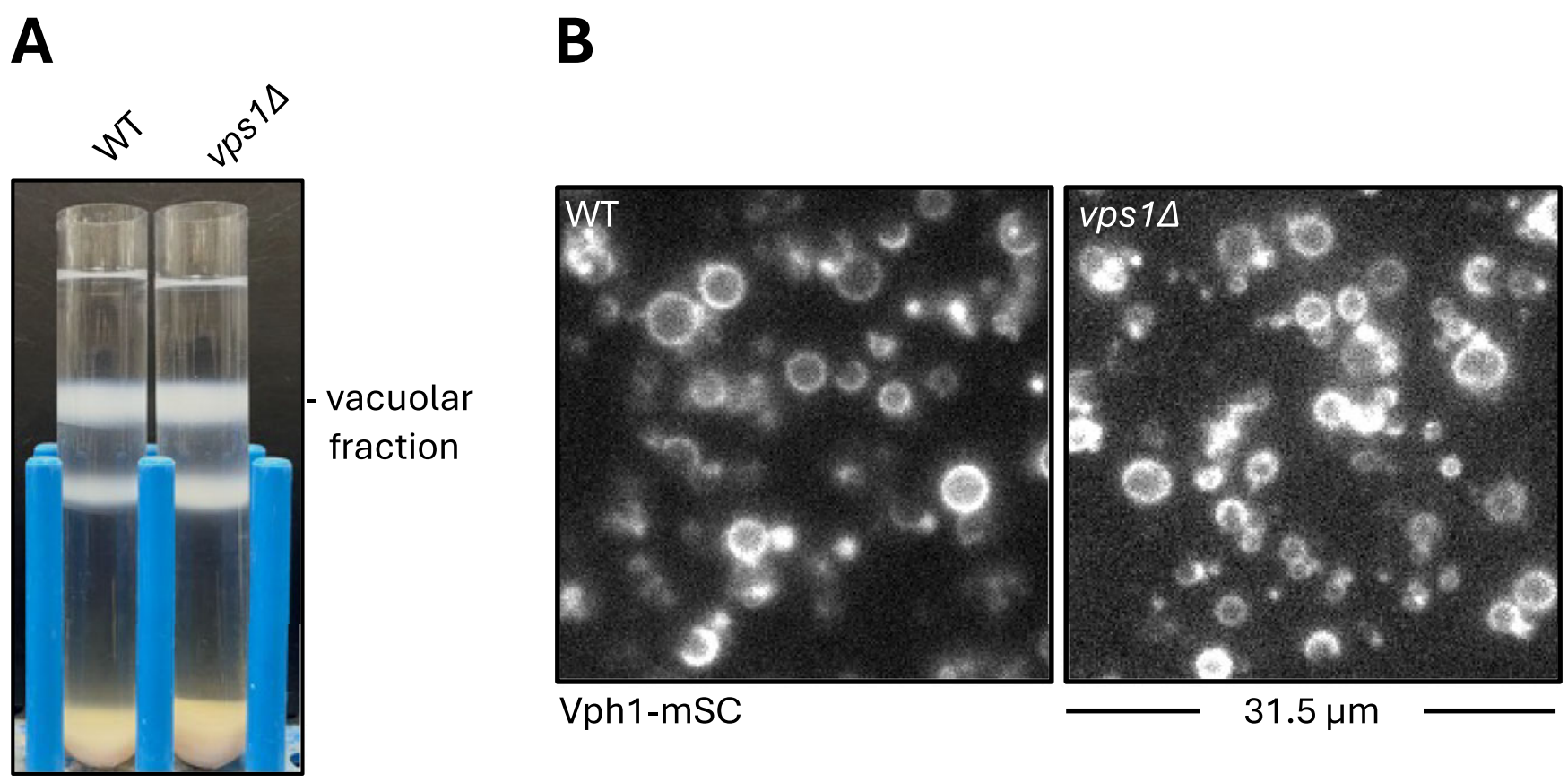

Figure EV4. Characteristics of vacuoles isolated from WT and *vps1Δ* strains.

(**A**) Density-gradient centrifugation-based separation of lysates from WT and *vps1Δ* cells, indicating the isolated vacuolar fraction. (**B**) Purified vacuoles from WT and *vps1Δ* cells expressing the vacuole marker Vph1-mSc.

 © The Author(s)
