# Supplementary material for: Vps1 drives membrane constriction and fission necessary for endosomal protein sorting
Source: EMBO J. 2026 Jul 20;45(17):6115–38. doi: 10.1038/s44318-026-00855-4 (PMC13534707; doi:10.1038/s44318-026-00855-4)
Supplement: Supplementary file 3 [file 44318_2026_855_MOESM12_ESM.zip › Movie EV1.docx]

**Movie EV1. Vps1-catalyzed membrane fission.** Movie showing the effect of flowing Vps1 with GTP on PC:PS:PI(3)P templates.
