# Supplementary material for: Vps1 drives membrane constriction and fission necessary for endosomal protein sorting
Source: EMBO J. 2026 Jul 20;45(17):6115–38. doi: 10.1038/s44318-026-00855-4 (PMC13534707; doi:10.1038/s44318-026-00855-4)
Supplement: Supplementary file 5 [file 44318_2026_855_MOESM14_ESM.zip › Movie EV3.docx]

**Movie EV3. Pathway to Vps1-catalyzed membrane fission.** Movie showing the effect of adding GTP to preassembled, GppNHp-bound Vps1 scaffolds on a membrane tube.
