# Supplementary material for: Vps1 drives membrane constriction and fission necessary for endosomal protein sorting
Source: EMBO J. 2026 Jul 20;45(17):6115–38. doi: 10.1038/s44318-026-00855-4 (PMC13534707; doi:10.1038/s44318-026-00855-4)
Supplement: Supplementary file 16 [file 44318_2026_855_MOESM25_ESM.zip › Movie EV14.docx]

**Movie EV14. Vps1(*Δ*3K) distribution in cells.** Movie showing the distribution of ALFA-NB-mNG in *VPS1*(Δ3K)* cells.
