# Supplementary material for: Vps1 drives membrane constriction and fission necessary for endosomal protein sorting
Source: EMBO J. 2026 Jul 20;45(17):6115–38. doi: 10.1038/s44318-026-00855-4 (PMC13534707; doi:10.1038/s44318-026-00855-4)
Supplement: Supplementary file 18 [file 44318_2026_855_MOESM27_ESM.zip › Movie EV16.docx]

**Movie EV16. Vps10 distribution in *VPS1(Δ4F)** cells.** Movie showing the distribution of Vps10-mNG (cyan) and Vph1-mSc (magenta) in *VPS1(Δ4F)** cells.
