# Supplementary material for: Vps1 drives membrane constriction and fission necessary for endosomal protein sorting
Source: EMBO J. 2026 Jul 20;45(17):6115–38. doi: 10.1038/s44318-026-00855-4 (PMC13534707; doi:10.1038/s44318-026-00855-4)
Supplement: Supplementary file 19 [file 44318_2026_855_MOESM28_ESM.zip › Movie EV17.docx]

**Movie EV17. Vps10 distribution in *VPS1(Δ3K)** cells.** Movie showing the distribution of Vps10-mNG (cyan) and Vph1-mSc (magenta) in *VPS1(Δ3K)** cells.
