# Supplementary material for: Vps1 drives membrane constriction and fission necessary for endosomal protein sorting
Source: EMBO J. 2026 Jul 20;45(17):6115–38. doi: 10.1038/s44318-026-00855-4 (PMC13534707; doi:10.1038/s44318-026-00855-4)
Supplement: Supplementary file 21 [file 44318_2026_855_MOESM30_ESM.zip › Movie EV19/Movie EV19.docx]

**Movie EV19. Neo1 distribution in WT cells.** Movie showing the distribution of Neo1-mNG (cyan) and Vph1-mSc (magenta) in wild-type cells.
