# Supplementary material for: Vps1 drives membrane constriction and fission necessary for endosomal protein sorting
Source: EMBO J. 2026 Jul 20;45(17):6115–38. doi: 10.1038/s44318-026-00855-4 (PMC13534707; doi:10.1038/s44318-026-00855-4)
Supplement: Supplementary file 26 [file 44318_2026_855_MOESM35_ESM.zip › Fig. 2/2B/README.rtf]

1. SMrTs with PC:PS:PI(3)P were prepared 
2. The bilayer and tubes were imaged for generating the calibration standard.
3. Vps1 was flowed with GTP and incubated for 10 mins.
4. Tubes were imaged.
5. Maximum membrane intensity was converted to tube radius using the calibration data for each set.
6. No. of tubes cut were scored and plotted against the tube radius.
