# Supplementary material for: Vps1 drives membrane constriction and fission necessary for endosomal protein sorting
Source: EMBO J. 2026 Jul 20;45(17):6115–38. doi: 10.1038/s44318-026-00855-4 (PMC13534707; doi:10.1038/s44318-026-00855-4)
Supplement: Supplementary file 26 [file 44318_2026_855_MOESM35_ESM.zip › Fig. 2/2K/README.rtf]

Estimating binding vs tube radius

	1	Tubes exposed to Ax488-labeled Vps1 were selected for analysis.
	2	50x512 pixel ROIs were cropped out. 
	3	The membrane channel was thresholded using Otsu to generate a mask. 
	4	Average protein and average membrane intensity as well as the maximum membrane intensity was estimated.
	5	Average protein intensity was divided by the average membrane intensity to get protein density.
	6	Maximum membrane intensity was converted to radius using the calibration.
	7	Protein density was plotted against tube radius
