# Supplementary material for: Vps1 drives membrane constriction and fission necessary for endosomal protein sorting
Source: EMBO J. 2026 Jul 20;45(17):6115–38. doi: 10.1038/s44318-026-00855-4 (PMC13534707; doi:10.1038/s44318-026-00855-4)
Supplement: Supplementary file 26 [file 44318_2026_855_MOESM35_ESM.zip › Fig. 2/2L/README.rtf]

Estimating constriction vs tube radius

	1	Before and after images of tubes exposed to Vps1 with GppNHp were acquired in the membrane channel.
	2	Images were converted to tube radius by applying the calibration.
	3	Final tube radius was plotted against the starting tube radius
