# Supplementary material for: Vps1 drives membrane constriction and fission necessary for endosomal protein sorting
Source: EMBO J. 2026 Jul 20;45(17):6115–38. doi: 10.1038/s44318-026-00855-4 (PMC13534707; doi:10.1038/s44318-026-00855-4)
Supplement: Supplementary file 30 [file 44318_2026_855_MOESM39_ESM.zip › Fig. 6/6B/README.rtf]

See Dataset EV3
